# E3 ligase FBXO2-mediated protein stability of insulin receptor regulates adipogenesis and metabolic health in obesity

**DOI:** 10.1038/s41419-026-09096-z

**Published:** 2026-07-08

**Authors:** Yue Xin, Jiaxin Liu, Zhaoyi Zhai, Haofeng Wu, Muchen Wu, Xueqin Wu, Ruiping Wang, Qi Wang, Xiaowei Jia, Lulu Wang, Hongwei Shang, Xin Lu, Meng Zhou, Tao Lu, Chun Yang, Dongliang Fang, Yan Gao

**Affiliations:** 1 Department of Human Anatomy, School of Basic Medical Sciences, Capital Medical University, Beijing, China; 2 Department of Cardiology, Beijing Luhe Hospital, Capital Medical University, Beijing, China; 3 Laboratory for Clinical Medicine, Beijing Key Laboratory for Tumor Invasion and Metastasis, Capital Medical University, Beijing, China; 4 Department of Experimental Center for Basic Medical Teaching, School of Basic Medical Sciences, Capital Medical University, Beijing, China; 5 Sports Medicine Department, Beijing Jishuitan Hospital, Capital Medical University, Beijing, China; 6 Key Laboratory of Major Diseases in Children, Ministry of Education, Beijing, China

**Keywords:** Cell biology, Biochemistry

## Abstract

Adipogenesis, a crucial physiological process, serves to safely sequester lipids, thereby preventing lipotoxicity in peripheral organs and preserving metabolic health during obesity. While insulin signaling plays a pivotal role in adipogenesis, regulating factors, especially the braking mechanism governing this process, warrant further investigation. Our study identified proteasome-dependent degradation of the insulin receptor (IR) during the early stages of adipogenesis as a critical event for the mitotic clonal expansion phase of the adipocyte differentiation program. A series of studies confirmed that the ubiquitinated modification of IR is regulated by E3 ligase FBXO2, and this is based on IR phosphorylation. We further elucidated that the FBD domain of FBXO2 is indispensable for its function in catalyzing p-IR ubiquitination. Gain or loss of function of Fbxo2 inhibited or promoted SVF or 3T3L1 cells proliferation and adipogenesis both in vitro and in vivo, which regulated adipose hyperplasia and plasticity of adipose tissue. Moreover, FBXO2 played an important role in regulating the metabolic health of mice when subjected to caloric excess. Collectively, our findings unveil FBXO2 as a negative regulator of adipogenesis by impairing the insulin signaling pathway.

**Figure:**
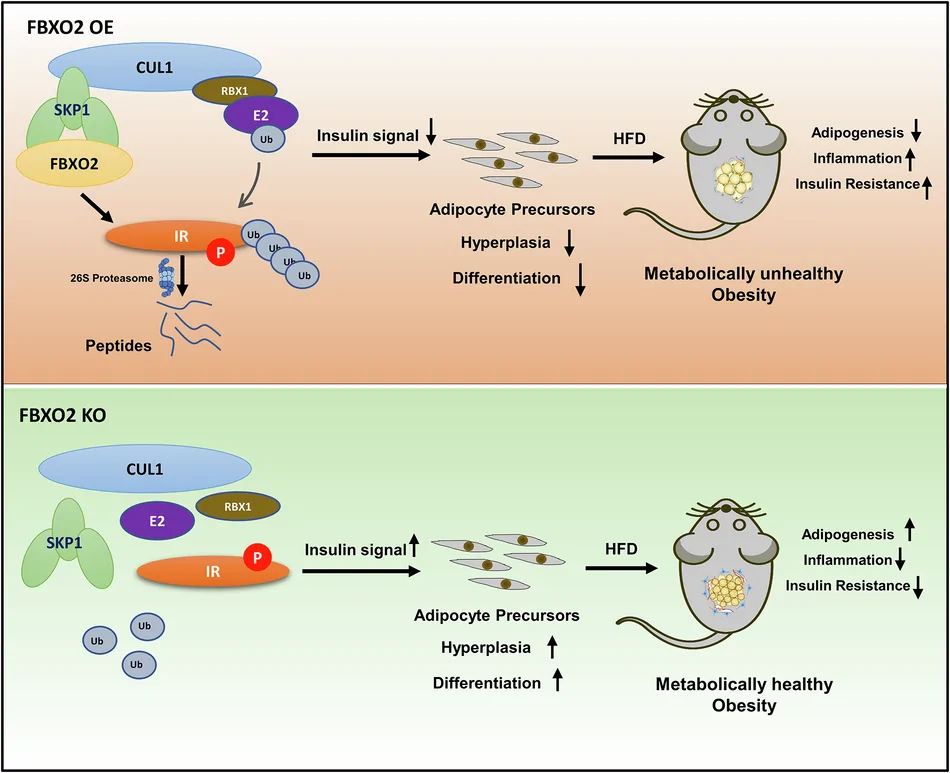

## Introduction

Obesity is characterized by the expansion of white adipose tissue (WAT), which primarily occurs in one of two ways: adipocyte size increase (hypertrophy) or adipocyte number rise (hyperplasia) [1–3]. Hypertrophy involves the enlargement of existing mature adipocytes, accompanied by lipid accumulation, necrosis, inflammatory cell infiltration, and tissue fibrosis, leading to adipose tissue dysfunction and complications such as insulin resistance. Conversely, hyperplasia refers to the formation of new adipocytes through precursor differentiation during adipogenesis. Shifting adipose tissue expansion from hypertrophy to hyperplasia could potentially prevent pathological remodeling and dysfunction of adipose tissue in response to excess caloric intake. Adipogenesis plays a crucial role in maintaining the normal physiological function of adipose tissue and overall metabolic health [4–6]. Therefore, research aimed at identifying new targets that promote adipogenesis holds significant potential for clinical interventions aimed at addressing obesity-related complications.

Numerous signaling hormones and ligands, including insulin, glucocorticoids, BMP, WNT, and hedgehog signaling, modulate the process of adipogenesis [1, 7]. Insulin, in particular, is a crucial component of in vitro adipogenic differentiation medium [8, 9]. Upon binding to its receptor, insulin initiates a signaling cascade involving IR, IRS1/2, PI3K, AKT1 or AKT2, mTOR, and the FOXO family. Activation of insulin signaling leads to the transcription of peroxisome proliferator-activated receptor-γ (PPARγ) and/or CCAAT/enhancer-binding protein-α (C/EBPα), facilitating preadipocyte differentiation [7, 10]. Given the physiological role of insulin in glucose metabolism, an interesting implication is that insulin serves as a permissive signal to adipogenesis under the condition of overnutrition [11–13]. It is of great significance to explore new approaches that regulate adipogenesis induced by insulin signaling. It has been reported that post-translational modifications of IR and IRS play crucial roles in regulating insulin signal transduction, particularly through ubiquitination and degradation [14]. For instance, the E3 ligase CHIP could catalyze IR ubiquitination modification to reduce insulin signaling and show an anti-aging effect in Drosophila and C. elegans [15]. Neddylation inhibition enhances insulin signaling by stabilizing IRS protein in hepatocytes and reduces hepatic glucose production [16]. Therefore, the stability of IR and/or IRS is closely linked to the activity of insulin signaling, making them pivotal targets for insulin signaling regulation.

E3 ligases play a pivotal role as they confer substrate specificity in the ubiquitination pathway [17–19]. They are involved in various cellular processes, including protein degradation, cell cycle, DNA repair, and signal transduction [20–22]. It was reported that E3 ligase APPBP2 degrades the browning key regulator PRDM16 by ubiquitination and inhibits the regeneration of beige fat, which plays an important role in obesity development and dyslipidaemia [23]. The deubiquitinating enzyme OTUD3 prevents obesity and diabetes by stabilizing PPARδ [24]. Additionally, E3 ligase CUL4B in adipocytes negatively regulates PPARγ-mediated adipose tissue expansion for combating obesity and metabolic syndromes [25]. Therefore, exploring the roles of E3 ubiquitin ligases in regulating adipogenesis holds significant promise for improving metabolic health.

This study aimed to identify novel factors associated with insulin signaling that modulate adipogenesis. We discovered that ubiquitination-dependent degradation of the insulin receptor occurs during the early stage of adipogenic differentiation. Through a series of ubiquitination experiments, we confirmed that this process is regulated by the E3 ligase FBXO2. Gene ectopic studies conducted both in vitro and in vivo validated FBXO2 as an inhibitor of adipogenesis. FBXO2 emerged as a negative regulator of metabolically beneficial WAT plasticity by ubiquitinating the insulin receptor and reducing insulin signaling activity. Our findings suggest FBXO2 as a potential therapeutic target for inhibiting metabolic complications such as insulin resistance and fatty liver.

## Research design and methods

### Animal models

In this study, Fbxo2-Flox mice, Fabp4-Cre mice, and Adipoq-Cre mice were purchased from Gempharmatech Co., Ltd (Nanjing, China). Pdgfrα-Cre mice were kindly provided by Dr. Tongjin Zhao in the Chinese Academy of Sciences, Shanghai Institute of Materia Medica (58). Fbxo2^flox/flox^ mice were bred to Pdgfrα-Cre, Fabp4-Cre and Adipoq-Cre mice to generate *Fbxo2*^Pdgfrα–/–^mice, *Fbxo2*^Fabp4–/–^mice and *Fbxo2*^Adipoq–/–^mice, respectively. All mice used in this study were on a C57BL/6 J genetic background. Male mice at 6–8 weeks of age were used for experiments. Mice were housed in a temperature (22 ± 2°C) and humidity (40–60%) controlled facility with regular 12:12 light/dark cycle and fed a rodent chow with free access to water. All animal experiments were performed in the animal facility of Capital Medical University (Beijing, China) and approved by the Animal Use and Care Committee of Capital Medical University. *Fbxo2*^Pdgfrα–/–^mice, *Fbxo2*^Fabp4–/–^mice and *Fbxo2*^Adipoq–/–^mice developed as normally as wildtype control mice and did not suffer from any spontaneous abnormalities when fed NCD. For HFD feeding experiments, 6–8 weeks old male mice were fed with HFD (60 kcal%, Research Diets, D12492) diets. Mouse genotyping was performed using genomic DNA isolated from mouse tails.

### Cell culture and adipocyte differentiation

HEK 293T cells and 3T3-L1 cells were cultured in high-glucose DMEM supplemented with 10% FBS and 1% penicillin/streptomycin at 37 °C in the presence of 5% CO2 and 95% air. For differentiation, the day when cells reached confluence was designated as day -2. For 3T3-L1 cells, two days after confluence (day 0), they were induced to differentiate with DMEM containing 10% FBS and 1% penicillin/streptomycin, 5 µg/mL insulin, 0.5 mmol/L 3-isobutyl-1-methylxanthine, and 1 mmol/L dexamethasone for 2 days (day 2); and maintained in the culture medium supplemented with 5 µg/mL insulin until the end (the medium was changed every 2 days).

### Primary SVF isolation and differentiation

SVF were isolated from the epididymal and subdermal fat pads of male mice, respectively (denoted as SVF eWAT and SVF sWAT, respectively). The epididymal or subdermal fat pads were washed with Krebs-Ringer solution and minced in Krebs-Ringer solution containing 0.8 mg/mL collagenase type I and 1% free fatty acid-free bovine serum albumin. The fat pads were then digested on a horizontal shaking at 37 °C for 50 min. The digestive mixture was filtered through 80 steel mesh and then 40 mesh cell strainers. After centrifugation at 1000 × *g* for 10 min, cell pellets were collected, suspended, and counted. The isolated cells were maintained in DMEM/F12 medium containing 10% FBS at 37 °C under an atmosphere of 5% CO_2_ and 95% air. For adipogenic differentiation, cells were incubated with differentiation medium I (DMI, DMEM/F12 supplemented with 5 µg/mL insulin, 0.5 mmol/L 3-isobutyl-1- methylxanthine, 1 mmol/L dexamethasone, 3 mmol/L indomethacin, 1% penicillin/streptomycin and 10% FBS) for 2 days and differentiation medium II (DMII, DMEM/F12 supplemented with 5 µg/mL insulin, 1% penicillin/streptomycin and 10% FBS) for another 2 days. After the induction of adipogenic differentiation, cells were cultured in maintenance media with 5 µg/mL insulin for another 4 days. The day of adding DMI was defined as the first day (day 0). In addition, cells were pretreated with MG132 at 20 µM for 2 h.

### Administration of adeno-associated viral (AAV) vectors

AAV-FBXO2 was used to overexpress FBXO2 in WAT, while AAV-GFP was used as the control. Inguinal WAT (iWAT) and epididymal WAT (eWAT) pads on both sides of mice were injected with the AAV. Mice were anesthetized with ketamine (100 mg/kg) and xylazine (10 mg/kg). For AAV delivery in iWAT, longitudinal incisions were made on the skin around the inguinal area, followed by exposition of the fat pad using tweezers. AAV was injected into each fat pad in multiple spots (8–10 spots per fat pad). The total volume was 50 μl, and the total virus titer was 1 × 10^11^ viral genomes (vg) for each pad. For AAV delivery in eWAT, laparotomy was performed to expose the eWAT, and the AAV was injected as described above. Then, the abdomen was rinsed with sterile saline solution and closed with a two-layer suture.

### Plasmid construction and cell transfection

For stable RNA interference, shCON and shFBXO2 were purchased from Genechem Co., Ltd (Shanghai, China). Plasmid encoding IR-Flag, FBXO2-Myc, FBXO2-Flag, Del1 (1-124), Del2 (1-95), Del3 (1-54), FBD (55-95), IR (Y1179A), IR (Y1180A), and IR (Y1179, 1180A) were purchased from Sangon Co., Ltd (Shanghai, China). Plasmid encoding HA-UB was kindly provided by Dr. Ping Xie at Capital Medical University. siRNA for mouse FBXO2 and negative control siRNA (siNC) were purchased from Sangon Co., Ltd (Shanghai, China). For cell transfection, the transient expression of genes was achieved using Lipofectamine with Plus reagents according to the instructions from the manufacturer. Transient siRNA transfection into SVF was performed in vitro using Lip2000 according to the manufacturer’s instructions. 48 h after transfection, SVF were used for experiments.

### LC-MS analyses

HEK 293T cells were transfected with NC, IR-Flag or FBXO2-Myc. Before harvest, cells were treated with 20 mM MG132 for 6 h. Cell pellets were lysed in NP-40 lysis buffer (50 mM HEPES, pH 8.0, 150 mM NaCl, 2.5 mM EGTA, 1 mM EDTA, and 0.1% Tween 20 with protease and phosphatase inhibitors). After centrifugation, the supernatant was applied for immunoprecipitation with anti-FLAG® M2 affinity gel (B23102, Bimake) overnight. The following day, beads were washed, and pulled-down proteins were eluted using Flag peptides (F3290, Sigma-Aldrich). Eluted proteins were separated by SDS PAGE gel and visualized with Pierce™ Silver Stain for Mass Spectrometry (24612, Thermo Fisher Scientific Inc). Excised bands were destained and sent out for LC-MS/MS at Capital Medical University. The data were analysed and proteins with at least two unique peptides were retained for further analysis.

### Cell viability assay

Cell viability was determined by the Cell Counting Kit-8 (CCK-8) assay. Briefly, cells were plated at a density of 10^4^ cells/well in 96-well plates. Cell viability was analyzed by using the CCK-8 kit according to the manufacturer’s protocol.

### Flow cytometry analysis

Adherent cells were trypsin-digested into single-cell suspensions, terminated with complete medium, and centrifuged at 1000 rpm for 5 min. Pellets were resuspended in pre-cooled 1×PBS for counting, then re-centrifuged. Cells were fixed in 0.5 mL pre-cooled 70% ethanol at 4 °C overnight, centrifuged, and washed with PBS to remove fixative. PI staining solution (per manufacturer’s instructions) was added (0.5 mL) for 37 °C dark incubation (30 min), then stored at 4 °C protected from light. Diluted, filtered samples were analyzed via a calibrated BD flow cytometer (10,000 cells/sample), detecting PI fluorescence (617 nm excitation, FL3 channel). FlowJo software quantified G1/G0, S, G2/M phases; *n* = 3, statistically analyzed.

### Real-time RT-PCR

Total RNAs were isolated from tissues or cells using TRIzol reagent (Takara Bio, China) according to the manufacturer’s instructions and converted into cDNA using a cDNA synthesis kit (Vazyme, China). RNA concentration was about 1500–3000 ng/μl, determined with NanoDrop2000. Real-time PCR analysis was performed using SYBR Green Master Mix (Vazyme, China) in CFX96 Real-time System, C1000 Thermal Cycler (Bio Rad). The primer sets we used were as Table S3.

### Western blot and immunoprecipitation

Total protein samples were isolated by treating tissue or cell samples with RIPA lysis buffer (65 mM Tris-HCl, pH 7.5, 150 mM NaCl, 1 mM EDTA, 1% NP-40, 0.5% sodium deoxycholate, 0.1% SDS, protease inhibitor cocktail tablets (P1265-2, Applygen, Beijing, China) and phosphatase inhibitor tablets (P1260-5, Applygen, Beijing, China)). The BCA Protein Assay Kit (23225, Thermo Fisher Scientific) was used to measure protein concentrations. Proteins were separated using 10% SDS-PAGE gels and then transferred to PVDF membranes (IPVH00010, Millipore). After the membranes were blocked in 5% skim milk, they were incubated overnight at 4 °C with primary antibodies and then for 1 h at room temperature with the corresponding secondary antibodies. The primary antibodies we used were as Table S4. After 3 washes in TBST, the immune complexes were detected using the ECL detection reagents (SQ201, Epizyme, Shanghai, China). Protein expression levels were quantified using Image Lab software and normalized to the levels of GAPDH, which was used as a loading control. Immunoprecipitation was performed using Pierce™ IP kit (26146, Thermo) following the manufacturer’s guidelines.

### Ubiquitination assay

HEK 293T cells were co-transfected with IR-Flag, HA-ubiquitin, and/or FBXO2-Myc. Before harvest, cells were treated with 20 mM MG132 for 6 h. Cells were lysed in IP lysis buffer. Then, subsequent experiments were performed using the Pierce™ IP Kit (26146, Thermo) following the manufacturer’s guidelines. Samples were boiled in SDS loading buffer and subjected to western blot using anti-HA antibody.

### Histology and immunohistochemistry

When animals were sacrificed, WAT and liver tissues were dissected and immediately fixed in 4% paraformaldehyde. Tissues were then routinely processed for paraffin embedding, and 5 mm sections were cut and mounted on glass slides. For hematoxylin-eosin, Masson trichrome, IHC, and EDU staining, the liver, sWAT, and eWAT were fixed overnight in 4% formalin, embedded in paraffin, and sectioned into sections 5 mm in thickness. The sections were stained using the corresponding kits (Masson trichome staining kits, G1340, Solebao, Beijing, China; EDU staining kit, C0071S/C0078S, Beyotime, Shanghai, China) according to the manufacturers’ instructions. Immunohistochemistry was performed on paraffin-embedded WAT and liver sections. Antigen retrieval was performed in citrate buffer using a pressure cooker. Immunohistochemistry (IHC) analysis was performed using an IHC detection kit (PV-9001, ZSGB-Bio, China) following the manufacturer’s guidelines. The sections were visualized with DAB (ZLI-9018; ZSGB-Bio, China) and counterstained with hematoxylin.

### Oil Red O staining

Differentiated SVF and 3T3-L1 cells were fixed with 4% formaldehyde in PBS for 30 min and then washed 3 times in PBS for 10 min. For Oil-Red-O staining, the cells were stained with 0.3% Oil Red O for 30 min in the dark at room temperature. Then washed 3 times in PBS for 10 min. Oil-Red-O-stained cells were directly imaged using an inverted microscope, or lipid content was quantified after Oil-Red-O extraction with isopropanol by determination of light absorbance at 490 nm.

### Glucose and insulin tolerance test

To determine glucose tolerance, 16-h fasted mice were intraperitoneally administered with glucose (1.5 g/kg of body weight). The blood glucose levels were measured from the tail blood before and 15, 30, 60, and 120 min after glucose injections. To determine insulin sensitivity, 6-h fasted mice were intraperitoneally administered insulin (0.75 U/kg of body weight). The blood glucose levels were measured from the tail blood before and 15, 30, and 60 min after insulin injections. Blood glucose from tail-vein blood was quantified by a Sinocare glucometer at designated times after administration.

### Biochemical analysis

Serum TG (A110-1), TC (A111-1), FFA (A042-2-1), ALT (C009-3-1) and AST (C010-3-1) levels were measured with biochemical assay kits from Nanjing Jiancheng Bioengineering Institute (Nanjing, China).

### Elisa assay

Serum insulin (CEA448Mu), leptin (SEA084Mu) and adiponectin (SEA605Mu) levels were measured with ELISA kits (Cloud-Clone, Wuhan, China).

### Metabolic assessment

We used a β3-adrenergic receptor agonist to activate brown fat. Mice were injected with CL316,243 (C5976; Sigma) at 1 mg/kg when needed. The core body temperature was monitored using a rectal probe (Omron, Dalian, China). The body temperature of mice was measured after CL316,243 injection. The body weight of mice was monitored weekly by a digital precision scale (accuracy 0.1 g). For energy metabolism analysis of mice, normal chow diet (NCD) feeding or CL316,243 injection, a total of 96 h of monitoring was conducted, and the data of the latter 48 h were taken for analysis. Energy expenditure measurements were made using a Comprehensive Lab Animal Monitoring System (Columbus Instruments). Mice were acclimated to the Comprehensive Lab Animal Monitoring System (CLAMS) for at least 24 h prior to acquisition of data.

### Statistical analysis

The statistical significance of differences between groups was examined using an unpaired two-tailed Student’s *t* test or a two-way ANOVA. All data are expressed as the mean ± SEM values and were analyzed using GraphPad Prism 8.0 (GraphPad Software Inc., San Diego, California, USA). Differences with *p* < 0.05 were defined as significant. If not otherwise mentioned, each experiment was repeated independently with similar results at least three times.

## Results

### Insulin receptor protein levels decline during adipogenic differentiation via FBXO2-mediated proteasomal degradation

**Fig. 1:**
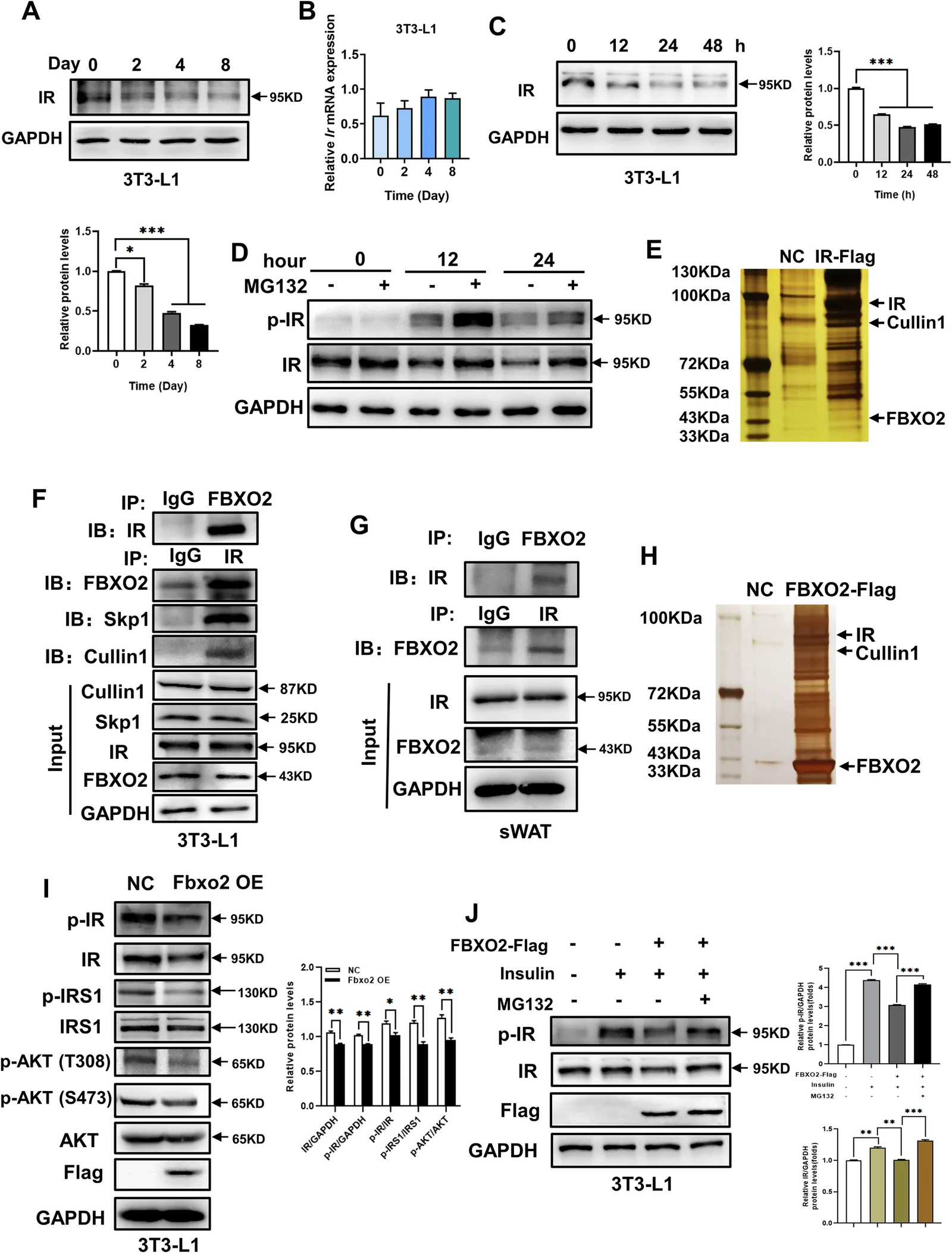
The decrease of IR protein during adipogenic differentiation is closely associated with E3 ligase FBXO2-mediated ubiquitin-dependent degradation. **A** Insulin receptor (IR) protein levels during adipogenic differentiation of 3T3-L1 cells at day 0, 2, 4, and 8 (upper panel). Protein densitometry analysis was conducted (lower panel). **B** Insulin receptor (IR) mRNA levels during adipogenic differentiation of 3T3-L1 cells at day 0, 2, 4, and 8. **C** IR protein levels during adipogenic differentiation of 3T3-L1 cells at h 0, 12, 24, and 48 (left panel). Protein densitometry analysis was conducted (right panel). **D** p-IR/IR protein levels during adipogenic differentiation of 3T3-L1 cells when treated with 20 μM MG132 for 2 h before harvest. **E** Immunoaffinity purification and mass spectrometry analysis of IR-interacted proteins. **F** Co-immunoprecipitation analysis of the association between IR and SCF^FBXO2^ complex. Whole cell lysates from 3T3-L1 cells were immunoprecipitated (IP) and immunoblotted (IB) with antibodies against the indicated proteins. **G** Co-immunoprecipitation of IR with FBXO2 in subcutaneous white adipose tissue of mice. **H** Immunoaffinity purification and mass spectrometry detection of the FBXO2-interacted proteins. **I** Protein levels of p-IR/IR, P-IRS1/IRS1, p-AKT(308)/AKT involved in the insulin signaling pathway in 3T3-L1 cells with FBXO2 overexpression (left panel). Protein densitometry analysis was conducted (right panel). **J** p-IR and IR protein levels transduced with NC or FBXO2 overexpression in 3T3-L1 cells, cells were incubated without or with 100 nM insulin treatment for 1 h and 20 μM MG132 treatment for 2 h (left panel). Protein densitometry of p-IR/GADPH and IR/GAPDH analysis was conducted (right panel). Statistical data are presented as mean ± SEM of three independent experiments, and statistical analysis was performed using unpaired *t*-test and two-way ANOVA. *P < 0.05, **P < 0.01, ***P < *0.001*, *n* = 3.

To explore the role of insulin receptor (IR) ubiquitination in adipogenesis, we examined IR expression at both transcriptional and translational levels during adipogenic differentiation of 3T3-L1 cells and stromal vascular fraction (SVF). A significant decrease in IR protein levels was observed at day 2 of differentiation of both 3T3L1 and SVF, while mRNA levels remained stable (Figs. 1A, B and S1A, B). This reduction in IR protein was evident as early as 12 h post-differentiation induction, with no change in mRNA levels (Figs. 1C and S1C, D, E). Given that ubiquitination is a key post-translational modification for protein degradation, we investigated whether IR reduction was linked to proteasome-dependent degradation using the proteasome inhibitor MG132. MG132 treatment significantly increased p-IR/IR protein levels during early adipogenic differentiation at 12 and 24 h (Figs. 1D and S1F, G).

We conducted immunoaffinity purification experiments using HEK293T cells and studied them in combination with mass spectrometry analysis. Firstly, the IR-Flag overexpression plasmid was transfected into HEK293T cells and treated with proteasome inhibitor MG132, then the cell lysate was prepared for electrophoresis and silver staining gel (Fig. 1E). Enriched proteins that coprecipitated with IR in the gel were further analyzed by mass spectrometry. Mass spectrometric analysis identified IR-interacting proteins, revealing that IR co-purified with FBXO2, Cullin1, and SKP1, components of the SCF^FBXO2^ complex. Detailed data of the mass spectrometry analysis are provided in Supplementary Material Table S1. Further verification of the interaction between FBXO2 and IR in the cell eluent was conducted through Western blot experiments (Fig. S2A). These interactions were also confirmed in 3T3-L1 cells, subcutaneous white adipose tissue (sWAT) and 293T cells by Co-immunoprecipitation (Co-IP) experiments (Figs. 1F, G and S2B). Then the FBXO2-Flag overexpression plasmid was transfected into HEK293T cells, and the cell lysate was prepared for electrophoresis, silver staining gel and mass spectrometry analysis (Fig. 1H). The interaction between FBXO2 and IR in the cell eluent was also conducted through Western blot (Fig. S2C). Detailed data of the mass spectrometry analysis are provided in Supplementary Material Table S2. Overexpression of FBXO2 reduced IR protein levels and downstream insulin signaling molecules (IRS and AKT) without affecting *Insr* mRNA levels (Fig. 1I and Fig. S2D, E). Conversely, FBXO2 knockdown increased IR and downstream molecule levels (Fig. S2F, G). The reduction in IR protein caused by FBXO2 was partially rescued by MG132 treatment (Fig. 1J). These findings indicate that IR protein is regulated by FBXO2-mediated proteasomal degradation during early adipogenic differentiation.

### FBXO2 catalyzes ubiquitination and degradation of phosphorylated IR

**Fig. 2:**
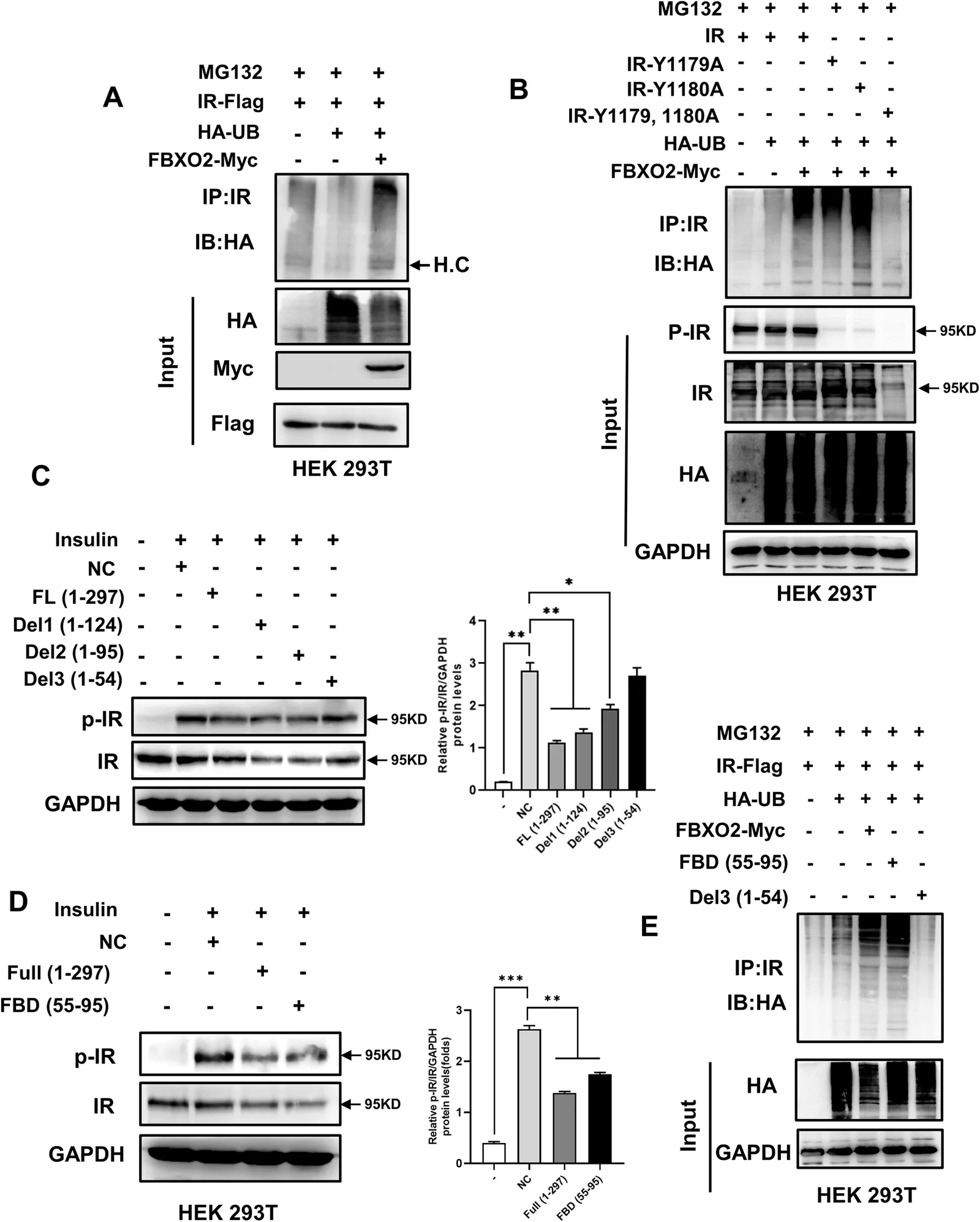
FBXO2 catalysed ubiquitination of the phosphorylated insulin receptor. **A** IR-Flag, HA-UB, and FBXO2-Myc were transfected in HEK 293T cells for ubiquitination assay, 20 μM MG132 was incubated for 2 h. **B** IR plasmid or IR phosphorylated mutants (Y1179A, Y1180A, Y1179A and Y1180A) were co-transfected with FBXO2-Myc and HA-Ub in 293T cells and ubiquitination assay was performed when inbubated with MG132. **C** p-IR/IR protein levels in HEK293T cells transfected with overexpression plasmids of FBXO2-Flag (1-297), Del1 (1-124), Del2 (1-95), and Del3 (1-54), cells were incubated without or with 100 nM insulin treatment for 1 h. Protein densitometry analysis was performed in the right panel. **D** p-IR/IR protein levels in HEK293T cells transfected with overexpression plasmids of FBXO2-Flag and FBD domain (55-95 aa), cells were incubated without or with 100 nM insulin treatment for 1 h. Protein densitometry analysis was performed in the right panel. **E** Flag-IR and HA-Ub were co-transfected with FBXO2-Myc or FBD (55-95 aa) or Del3 (1-54 aa) in 293T cells, and ubiquitination assay was performed when incubated with MG132. Statistical data are presented as mean ± SEM of three independent experiments, and statistical analysis was performed using unpaired *t*-test and two-way ANOVA. *P < 0.05, **P < 0.01, ***P < *0.001*, n = 3.

We next tested whether IR is a direct substrate of FBXO2. In vitro ubiquitination assays confirmed that FBXO2 modifies IR ubiquitination (Fig. 2A). Notably, phosphorylated IR (p-IR) was more significantly altered than total IR (Fig. 1I, J). These results indicate that phosphorylation might be the premise for IR ubiquitination. To test this, we generated IR mutants at tyrosine sites Y1179 and Y1180. Ubiquitination assays showed that IR ubiquitination was unaffected by single mutations at Y1179 or Y1180 but was nearly abolished with double mutations (Fig. 2B). Thus, FBXO2-mediated IR ubiquitination is based on its phosphorylation.

FBXO2 contains four domains: PEST, F-box (FBD), linker, and sugar-binding (SBD). To elucidate the role of these domains, we constructed FBXO2 fragments (Fig. S2H). The results showed that only Del3 (1-54) cannot affect p-IR/IR levels among the three fragments of Del1 (1-124), Del2 (1-95), and Del3 (1-54), which indirectly reflects that FBD (55-95) is the key fragment (Fig. 2C). So we further constructed FBD fragments to directly demonstrate that FBD is the core structural domain for FBXO2 (Fig. 2D). Ubiquitination assays confirmed that the FBD is crucial for IR multi-ubiquitination (Fig. 2E). These results demonstrate that phosphorylation is essential for IR ubiquitination and degradation, and the FBD is indispensable for FBXO2 function.

### FBXO2 inhibits proliferation and adipogenic differentiation of preadipocytes by attenuating the insulin signaling pathway in vitro

**Fig. 3:**
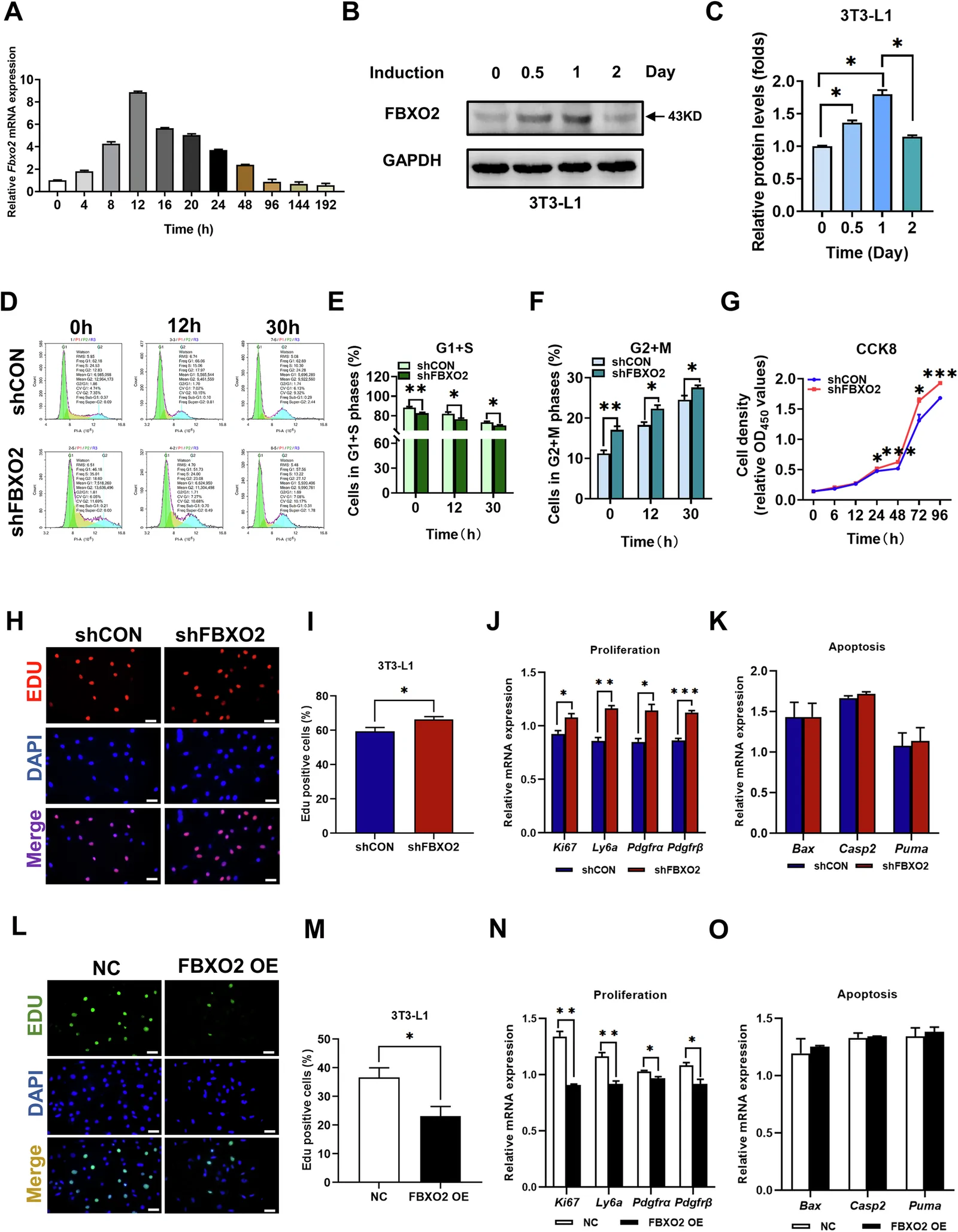
FBXO2 inhibits the proliferation of preadipocytes. **A** *Fbxo2* mRNA levels at the indicated time points during adipogenic differentiation of SVF cells. **B** FBXO2 protein levels during adipogenic differentiation of 3T3L1 cells at day 0, 0.5, 1, 2, and 4. **C** Protein densitometry analysis was performed for (**B**). **D** Flow cytometry of cell cycle phase distribution after FBXO2 knockdown during adipogenic differentiation of 3T3-L1 cells at h 0, 12, and 30. **E, F** Statistical analysis was performed for (**D**). **G** CCK-8 assay was used to determine the proliferation of 3T3-L1 cells after FBXO2 knockdown. **H** The rate of EDU-positive cells in 3T3-L1 cells after FBXO2 knockdown, scale bars, 50 μm. **I** Statistical analysis was performed for (**H**). mRNA levels of proliferation (**J**) and apoptosis (**K**) genes of 3T3-L1 cells after FBXO2 knockdown. The rate of EDU-positive cells in 3T3-L1 cells after FBXO2 overexpression, scale bars, 50 μm. **M** Statistical analysis was performed for (**L**). mRNA levels of proliferation (**N**) and apoptosis (**O**) genes of 3T3-L1 cells after FBXO2 overexpression. Statistical data are presented as mean ± SEM of three independent experiments, and statistical analysis was performed using unpaired *t*-test. *P < 0.05, **P < 0.01, ***P < *0.001*, *n* = 3.

Given that FBXO2 weakens insulin signaling by degrading p-IR, we hypothesized that FBXO2 regulates adipogenic differentiation. *Fbxo2* mRNA levels increased during early adipogenic differentiation and peaked after 12 h induction (Figs. 3A and S3A, B). FBXO2 protein levels also increased during early differentiation (Figs. 3B, C and S3C, D). The early stage of adipocyte differentiation is for preadipocytes mitotic clonal proliferation [26, 27]. We observed that knockdown of FBXO2 decreased the G1 + S phase and increased the G2 + M phase in preadipocytes (Fig. 3D–F), promoted cell viability by CCK8 (Fig. 3G), and enhanced cell proliferation by EDU staining (Fig. 3H, I). Meanwhile, knockdown of FBXO2 upregulated proliferation genes (*Ki67*, *Ly6a*, *Pdgfrα*, *Pdgfrβ*), without affecting apoptosis genes (*Bax*, *Casp2*, *Puma*) (Fig. 3J, K). Conversely, FBXO2 overexpression reduced proliferation and downregulated proliferation genes, while apoptosis genes remained unchanged (Fig. 3L–O). FBXO2 knockdown also inhibited SVF proliferation (Fig. S3E–I), underscoring its role in preadipocyte proliferation.

**Fig. 4:**
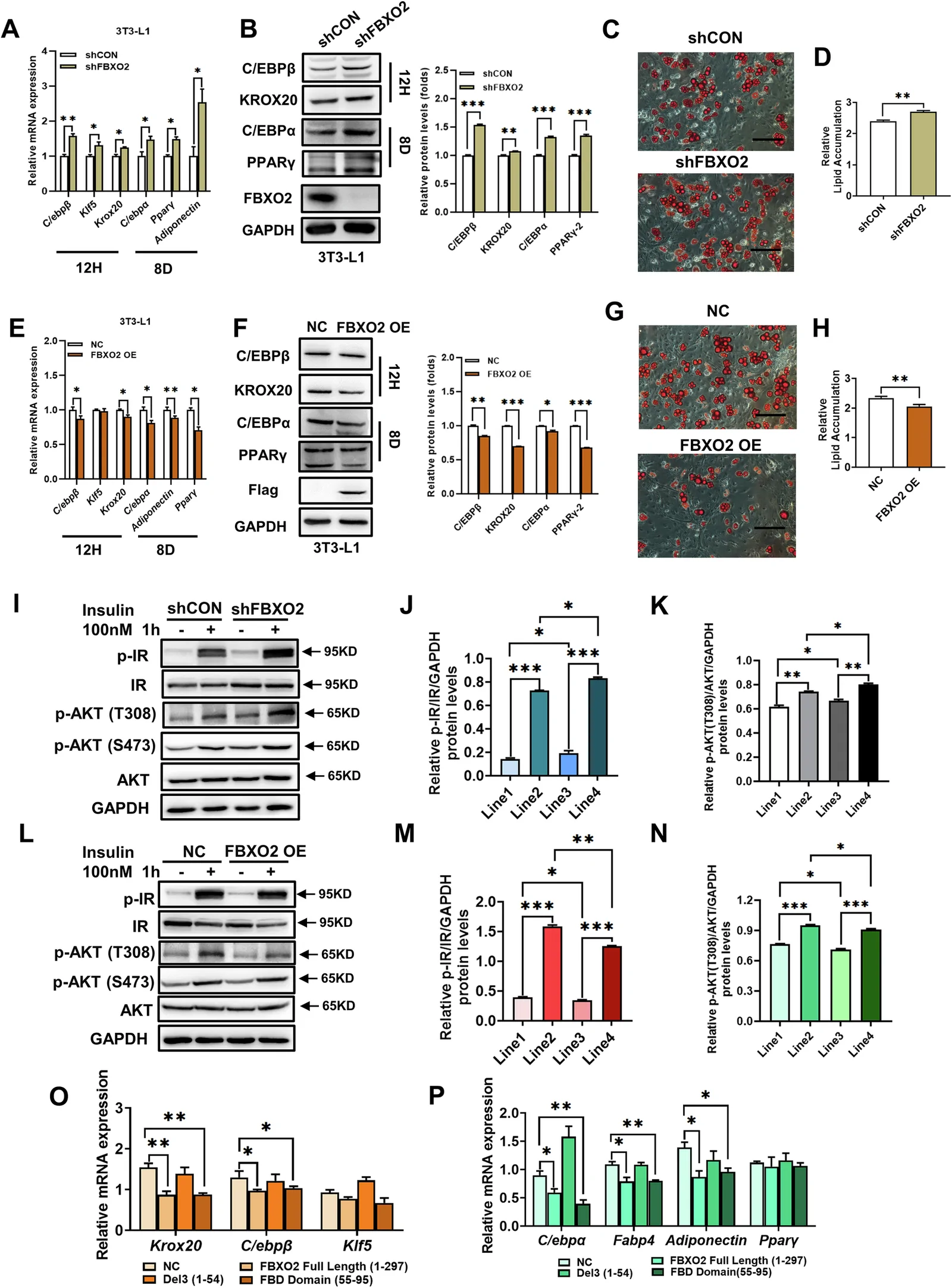
FBXO2 inhibits adipogenesis by negatively regulating the insulin signaling pathway in vitro. mRNA (**A**) and protein (**B**) levels of adipogenic genes during adipogenic differentiation of 3T3-L1 cells with FBXO2 knockdown. Protein densitometry analysis was conducted in the right panel. **C** Oil red O staining of lipid accumulation in differentiated 3T3-L1 cells with FBXO2 knockdown, scale bars, 20 μm. **D** Spectrophotometric measurement was used to determine the absorbance values of Oil Red O staining solution. mRNA (**E**) and protein (**F**) levels of adipogenic genes during differentiation of 3T3-L1 cells with FBXO2 overexpression. Protein densitometry analysis was conducted in the right panel. **G** Oil red O staining to detect lipid accumulation in 3T3-L1 cells with FBXO2 overexpression, scale bars, 20 μm. **H** Spectrophotometric measurement was used to determine the absorbance values of Oil red O staining solution. **I** Protein levels of p-IR/IR, p-AKT(308)/AKT in 3T3-L1 cells with FBXO2 knockdown, cells were treated with 100 nM insulin for 1 h before harvest. **J, K** Protein densitometry analysis was conducted for (I). **L** Protein levels of p-IR/IR, p-AKT (308) /AKT in 3T3-L1 cells with FBXO2 overexpression, cells were treated with 100 nM insulin for 1 h before harvest. **M, N** Protein densitometry analysis was conducted for (**L**). mRNA levels of early (**O**) and late-stage (**P**) adipogenic genes in 3T3-L1 cells with overexpression of FBXO2-Flag, Del3 (1-54), and FBD (55-95). Statistical data are presented as mean ± SEM of three independent experiments, and statistical analysis was performed using unpaired *t*-test and two-way ANOVA. *P < 0.05, **P < 0.01, ***P < *0.001*, *n* = 3.

During adipogenic differentiation, FBXO2 knockdown enhanced early factor induction (*C/ebpβ*, *Klf5*, *Krox20*) and adipogenic gene expression (*C/ebpα*, *Adiponectin*, *Pparγ*) (Fig. 4A, B). Oil Red O staining showed increased lipid accumulation with FBXO2 knockdown (Fig. 4C, D). Conversely, FBXO2 overexpression inhibited adipogenic gene expression and lipid accumulation (Fig. 4E–H). FBXO2 knockdown increased p-IR/IR and p-AKT/AKT levels (Fig. 4I–K), while overexpression inhibited insulin signaling (Fig. 4L–N). FBD overexpression also inhibited adipogenic gene expression (Fig. 4O, P). Additionally, FBXO2 knockdown promoted adipogenic gene induction and lipid accumulation in sWAT but not eWAT (Fig. S4A–F). Collectively, FBXO2 suppresses adipogenic differentiation by inhibiting insulin signaling in vitro.

### FBXO2 deficiency in adipocyte progenitors enhances adipogenesis in sWAT by augmenting insulin signaling

Based on the previous cell experiments, we next investigated the biological function of FBXO2 in regulating adipogenesis by constructing mouse models. This study investigates the regulatory role of FBXO2 in adipogenesis, which refers to the physiological process of adipocyte precursor cells differentiating into mature adipocytes. Based on our findings of adipogenic differentiation studies in vitro, it is suggested that FBXO2 mainly functions in the early stage, not the mature period of adipogenesis. Therefore, we constructed gene knockout mouse models with three types of Cre mice. First, we chose the Pdgfra-Cre mice, which are the most commonly used for targeting adipocyte precursor cells in the study of adipogenesis. The second one is the Fabp4-Cre, which is a classic tool mouse used for studying adipose function, although its lack of specificity in targeting limits its application. The third one is adipose tissue-specific promoter Adiponectin-Cre that targets the mature adipocytes, which might not be useful for studying the role of FBXO2 in adipogenesis.

**Fig. 5:**
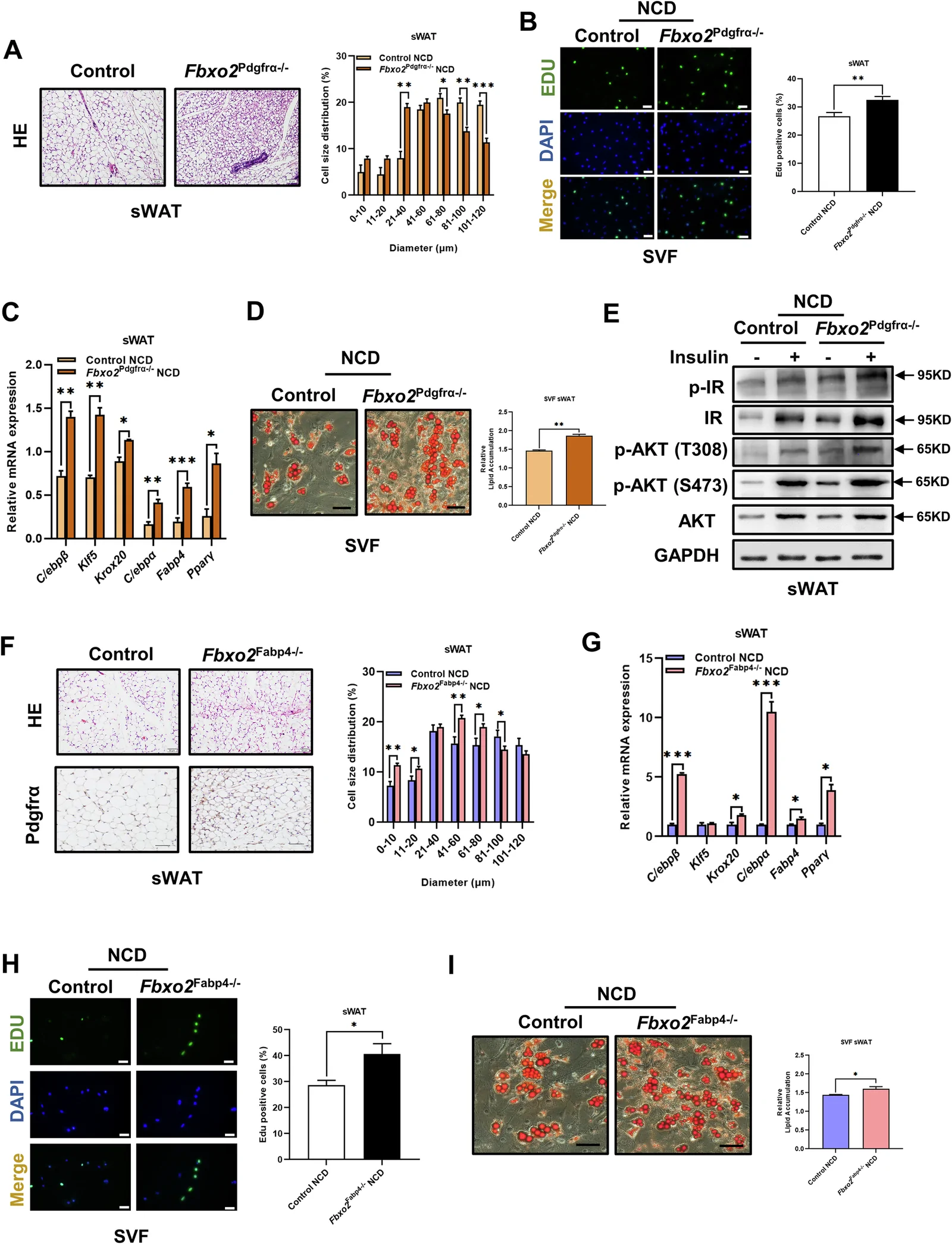
FBXO2 deficiency in adipocyte progenitors promoted adipogenesis by enhancing the insulin signaling. **A** HE staining of sWAT in control and *Fbxo2*^Pdgfrα–/–^ mice, scale bar, 50 μm. Statistical analysis of adipocyte number and size in the right panel. **B** EdU staining of SVF from sWAT of NCD-fed control and *Fbxo2*^Pdgfrα–/–^ mice, scale bars, 50 μm. Statistical analysis of the EdU-positive cells in the right panel. **C** mRNA levels of proliferation, apoptosis, and adipogenic genes in sWAT of control and *Fbxo2*^Pdgfrα–/–^ mice. **D** Oil red O staining to detect lipid accumulation in SVF cells from sWAT of control and *Fbxo2*^Pdgfrα–/–^ mice, scale bar, 20 μm. Spectrophotometric measurement was used to determine the absorbance values of Oil red O staining solution in the right panel. **E** Protein levels of p-IR/IR, p-AKT(308)/AKT in sWAT of control and *Fbxo2*^Pdgfrα–/–^ mice following intraperitoneal insulin injection fed with NCD. **F** HE and IHC staining of sWAT sections in control and *Fbxo2*^Fabp4–/–^ mice fed with NCD, scale bar, 50 μm. Statistical analysis of adipocyte number and size in the right panel. **G** mRNA levels of proliferation, apoptosis, and adipogenic genes in sWAT of control and *Fbxo2*^Fabp4–/–^ mice fed with NCD. **H** EdU staining of SVF from sWAT of NCD-fed control and *Fbxo2*^Fabp4–/–^ mice, scale bars, 50 μm. Statistical analysis of the EdU-positive cells in the right panel. **I** Oil red O staining to detect lipid accumulation in SVF cells from sWAT of control and *Fbxo2*^Fabp4–/–^ mice, scale bar, 20 μm. Spectrophotometric measurement was used to determine the absorbance values of Oil red O staining solution in the right panel. Statistical data are presented as mean ± SEM of three independent experiments, and statistical analysis was performed using unpaired *t*-test and two-way ANOVA. *P < 0.05, **P < 0.01, ***P < *0.001*, *n* = 6.

In the first knock-out mouse model, mice carrying the floxed-Fbxo2 allele containing two loxP sites were crossed with Pdgfrα-Cre mice to generate *Fbxo2*^Pdgfrα–/–^ mice. FBXO2 knock-out was validated in sWAT and eWAT but not the liver by Western Blot (Fig. S5A). Body weight of *Fbxo2*^Pdgfrα–/–^ mice was comparable to control under NCD feeding (Fig. S5B). Subcutaneous fat mass of *Fbxo2*^Pdgfrα–/–^ mice exhibited a higher ratio than the control group (Fig. S5C). Histological analysis revealed a higher number of adipocytes with a smaller size in sWAT from *Fbxo2*^Pdgfrα–/–^ mice compared to control (Fig. 5A). EdU tracing experiments revealed higher vitality of adipogenic progenitors in sWAT from *Fbxo2*^Pdgfrα–/–^ mice compared to control (Fig. 5B). mRNA levels of adipocyte progenitor markers and adipogenesis genes in sWAT of *Fbxo2*^Pdgfrα–/–^ mice were upregulated while the expression of apoptotic genes was comparable between the two groups (Figs. 5C and S5D). Differentiated SVF isolated from sWAT of *Fbxo2*^Pdgfrα–/–^ mice showed a higher amount of lipid droplet accumulation by Oil Red O staining (Fig. 5D). Meanwhile, protein levels of p-IR/IR and p-AKT/AKT were increased in sWAT of *Fbxo2*^Pdgfrα–/–^ mice (Figs. 5E and S5E). In eWAT, mRNA levels of adipogenic genes were not altered in *Fbxo2*^Pdgfrα–/–^ mice compared with control (Fig. S5F).

In the second knockout model, we generated *Fbxo2*^Fabp4–/–^ mice by crossing Fbxo2-Flox with Fabp4-Cre mice. FBXO2 knock-out was validated in sWAT and eWAT but not the liver (Fig. S5G). There was also no discernible difference between control and *Fbxo2*^Fabp4–/–^ mice in body weight, while the ratio of sWAT was also mildly elevated in *Fbxo2*^Fabp4–/–^ mice (Fig. S5H, I). Notably, *Fbxo2*^Fabp4–/–^ mice exhibited an increased number of PDGFRα-positive adipocytes with a smaller size in sWAT compared to control (Fig. 5F). The expression of adipogenic genes in sWAT of *Fbxo2*^Fabp4–/–^ mice was higher than that of control (Fig. 5G). EdU tracing experiments revealed higher vitality of adipogenic progenitors in sWAT from *Fbxo2*^Fabp4–/–^ mice compared to control (Fig. 5H). Lipid accumulation was increased in differentiated SVF of *Fbxo2*^Fabp4–/–^ mice than control (Fig. 5I). FBXO2 deficiency led to higher protein levels of p-IR/IR and p-AKT/AKT, indicating an enhanced insulin signaling pathway in sWAT of *Fbxo2*^Fabp4–/–^ mice (Fig. S5J).

In the third mouse model, we generated *Fbxo2*^Adipoq–/–^ mice by using Adipoq-Cre mice. As we predicted, FBXO2 was not successfully knocked down in the adipose tissue of mice (Fig. S6A). A series of studies, including histological, Edu, and gene analysis showed that there was no discernible difference in adipogenesis between *Fbxo2*^Adipoq–/–^ mice and control group (Fig. S6B–D). In summary, these results indicate that FBXO2 deficiency in preadipocytes rather than adipocytes promotes adipogenesis in mice.

### FBXO2 deficiency improves metabolic health in obese mice

**Fig. 6:**
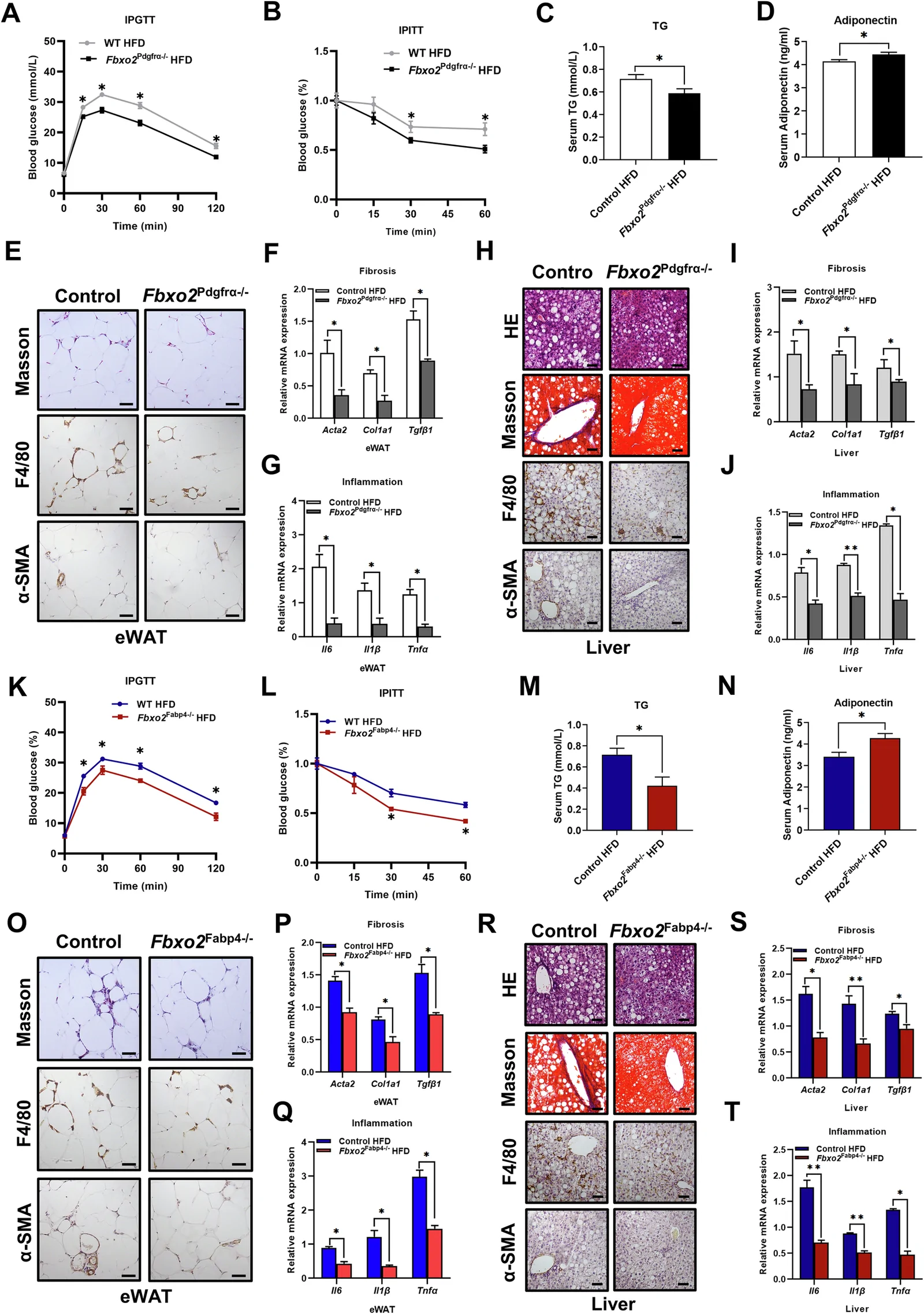
FBXO2 deficiency in preadipocytes improved systemic metabolic health. Glucose tolerance test (**A**) and insulin tolerance test (**B**) of control and *Fbxo2*^Pdgfrα–/–^ mice fed with HFD. Serum triglycerides (TG) (**C**) and adiponectin (**D**) levels of control and *Fbxo2*^Pdgfrα–/–^ mice fed with HFD. **E** Masson staining, IHC staining of F4/80 and α-SMA in eWAT of *Fbxo2*^Pdgfrα–/–^ and control mice fed with HFD, scale bar, 50 μm. mRNA levels of fibrosis (**F**) and inflammatory genes (**G**) of eWAT in control and *Fbxo2*^Pdgfrα–/–^ mice fed with HFD, scale bar, 50 μm. **H** HE staining, Masson staining, and IHC staining of F4/80 and α-SMA in livers of control and *Fbxo2*^Pdgfrα–/–^ mice fed with HFD, scale bar, 50 μm. mRNA levels of fibrosis (**I**) and inflammatory genes (**J**) in the liver of control and *Fbxo2*^Pdgfrα–/–^ mice fed with HFD, scale bar, 50 μm. Glucose tolerance test (**K**) and insulin tolerance test (**L**) of control and *Fbxo2*^Fabp4–/–^ mice fed with HFD. Serum triglycerides (TG) (**M**) and adiponectin (**N**) levels of control and *Fbxo2*^Fabp4–/–^ mice fed with HFD. **O** Masson staining, IHC staining of F4/80 and α-SMA in eWAT of control and *Fbxo2*^Fabp4–/–^ mice fed with HFD, scale bar, 50 μm. mRNA levels of fibrosis (**P**) and inflammatory genes (**Q**) in eWAT of control and *Fbxo2*^Fabp4–/–^ mice fed with HFD, scale bar, 50 μm. **R** HE staining, Masson staining, and IHC staining of F4/80 and α-SMA in the liver of control and *Fbxo2*^Fabp4–/–^ mice fed with HFD, scale bar, 50 μm. mRNA levels of fibrosis (**S**) and inflammatory genes (**T**) in livers of control and *Fbxo2*^Fabp4–/–^ mice fed with HFD, scale bar, 50 μm. Statistical data are presented as mean ± SEM of three independent experiments, and statistical analysis was performed using an unpaired *t*-test. *P < 0.05, **P < 0.01, ***P < *0.001*, *n* = 6.

We next investigated whether the effects of FBXO2 in regulating adipogenesis have an impact on improving the metabolism of obese mice. It was shown that FBXO2 deficiency enhanced glucose tolerance and insulin sensitivity in *Fbxo2*^Pdgfrα–/–^ mice under HFD feeding (Fig. 6A, B). Serum levels of triglyceride (TG), total cholesterol (TC), free fatty acid (FFA), alanine aminotransferase (ALT), aspartate aminotransferase (AST) and insulin were lower, while adiponectin was higher in *Fbxo2*^Pdgfrα–/–^ mice (Figs. 6C, D and S7A–F). Next, we assessed fibrosis and inflammation in adipose and liver tissue by Masson staining and immunohistochemical assays. Notably, eWAT and sWAT of *Fbxo2*^Pdgfrα–/–^ HFD mice exhibited alleviative fibrosis and inflammation compared to control (Figs. 6E and S7G). Additionally, the expression of genes associated with fibrosis (*Acta2*, *Col1a1*, *Tgfβ1*) and inflammation (*Il6*, *Il1β*, *Tnfα*) was markedly lower in eWAT and sWAT of *Fbxo2*^Pdgfrα–/–^ mice than in controls with an HFD feeding (Figs. 6F, G and S7H, I). Histological examination showed a pronounced alleviative lipid infiltration, fibrosis, and inflammation in the liver of *Fbxo2*^Pdgfrα–/–^ HFD mice compared to controls (Fig. 6H). The expression of genes associated with fibrosis, inflammation, mitochondrial, hypoxia, and macrophage markers was also markedly lower in the liver of *Fbxo2*^Pdgfrα–/–^HFD mice than in control mice (Figs. 6I, J and S7J). In the *Fbxo2*^Fabp4–/–^ mice, FBXO2 deficiency also improved glucose tolerance and insulin sensitivity of obese mice (Fig. 6K, L). Serum biochemical parameters as mentioned above, are also improved in *Fbxo2*^Fabp4–/–^ mice than controls (Fig. 6M, N and S7K–P). *Fbxo2*^Fabp4–/–^ mice also exhibited alleviated inflammation and fibrosis in adipose tissue and liver (Figs. 6O–T and S7Q–T). In analysis of *Fbxo2*^Adipoq–/–^ mice, it was shown that there was no obviously change in their whole body metabolism (Fig. S8A–K). Taken together, these data suggest that FBXO2 deficiency in preadipocytes significantly contributes to the amelioration of systemic metabolic health of obese mice.

Given the pivotal roles of brown and beige fat biogenesis in regulating metabolic health [28–32]. We conducted a comprehensive assessment of various metabolic parameters in both *Fbxo2*^Pdgfrα–/–^ and control mice under basal conditions or CL316,243 stimulation. Our analysis revealed no significant differences in thermogenesis, energy expenditure and whole body metabolism between control and *Fbxo2*^Pdgfrα–/–^ mice (Fig. S9A–T).

### FBXO2 overexpression inhibits adipogenesis and exacerbates metabolic syndrome

**Fig. 7:**
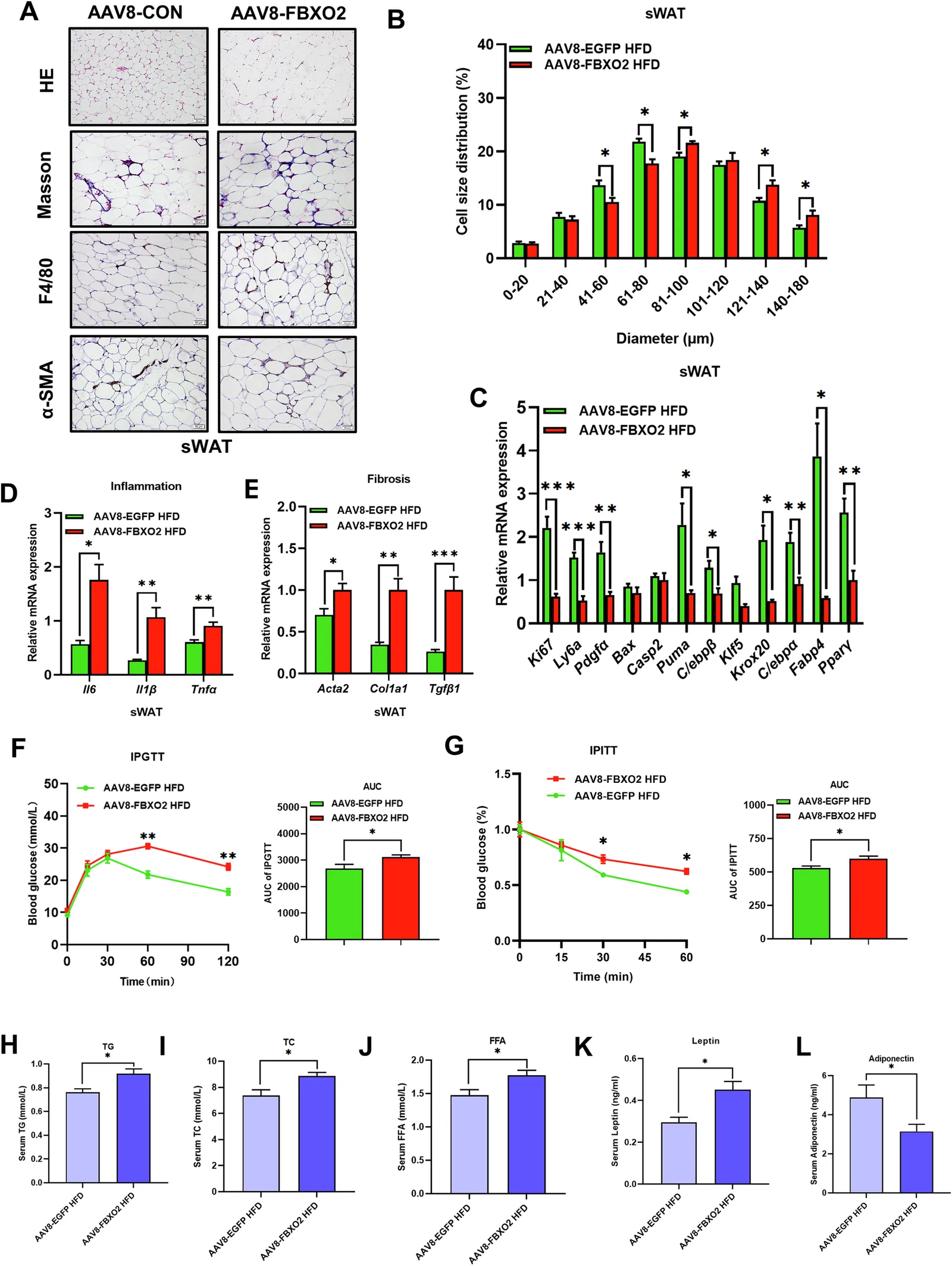
FBXO2 overexpression inhibited adipogenesis and aggravated the metabolic health of HFD-induced obese mice. **A** Representative images of sWAT sections from HFD-fed AAV-GFP and AAV-FBXO2 mice by HE, Masson staning, and IHC for F4/80, α-SMA, scale bar, 50 μm. **B** Statistical analysis of adipocyte number and size in (**A**). **C** RT-PCR analysis for proliferative, apoptosis and adipogenic genes in sWAT of HFD-fed AAV-GFP and AAV-FBXO2 mice. RT-PCR analysis of inflammatory (**D**) and fibrosis (**E**) genes expression in sWAT of HFD-fed AAV-GFP and AAV-FBXO2 mice. GTT (**F**) and ITT (**G**) analysis of HFD-fed AAV-GFP and AAV-FBXO2 mice. AUC (Area Under the Curve) analysis for each right panel. Serum levels of TG (**H**), TC (**I**), FFA (**J**), Leptin (**K**), and Adiponectin (**L**) of HFD-fed AAV-GFP and AAV-FBXO2 mice. Statistical data are presented as mean ± SEM of three independent experiments, and statistical analysis was performed using unpaired t-test. *P < 0.05, **P < 0.01, ***P < *0.001*, *n* = 8.

**Fig. 8:**
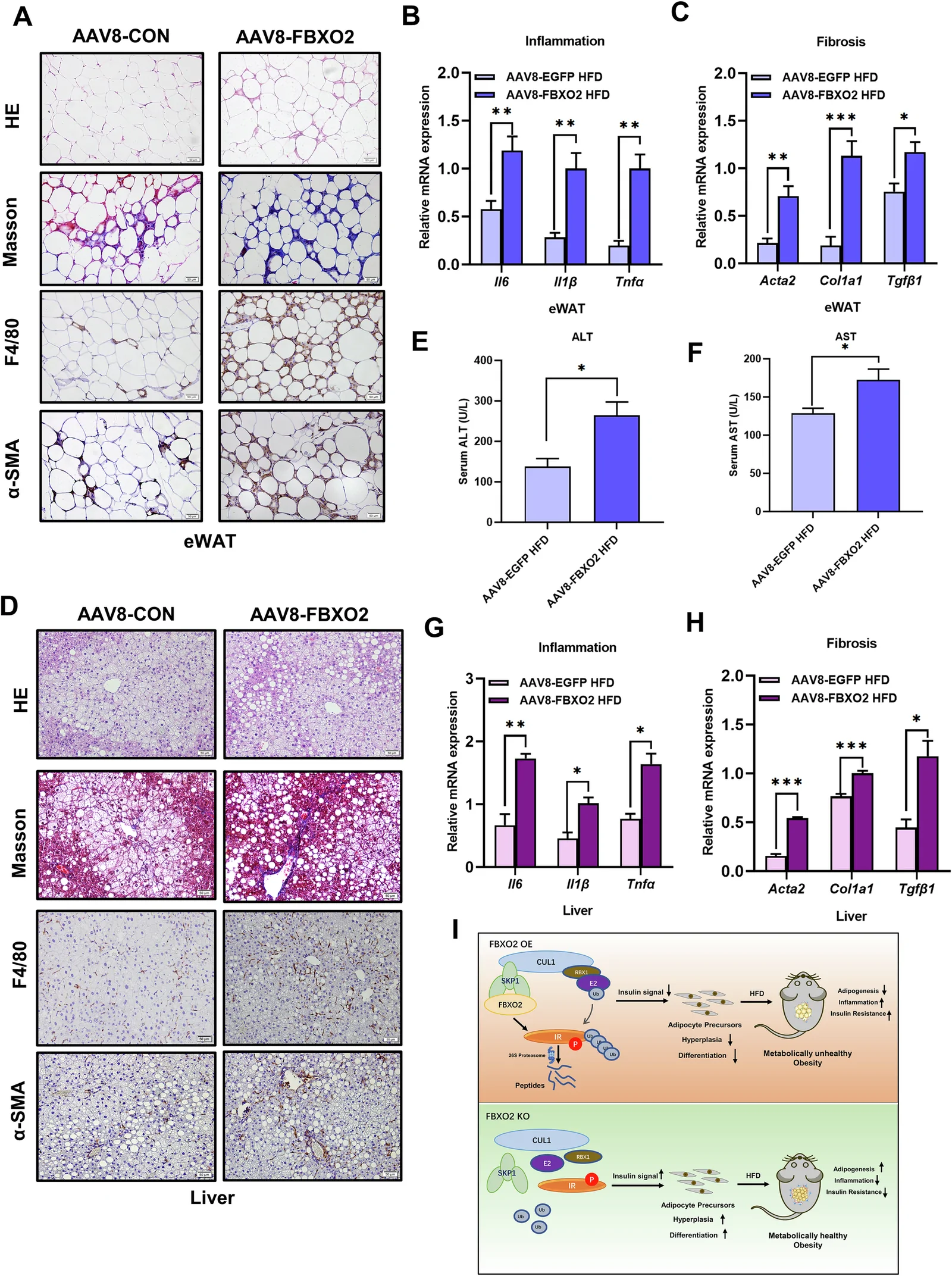
FBXO2 overexpression aggravated the dysfunction of eWAT and the liver of obese mice. **A** Representative images of eWAT sections from HFD-fed AAV-GFP and AAV-FBXO2 mice by HE, Masson staning, and IHC for F4/80, α-SMA, scale bar, 50 μm. qRT-PCR analysis of inflammatory (**B**) and fibrosis (**C**) genes expression in eWAT of HFD-fed AAV-GFP and AAV-FBXO2 mice. **D** Representative images of liver sections from HFD-fed AAV-GFP and AAV-FBXO2 mice by HE, Masson staning, and IHC for F4/80, α-SMA, scale bar, 50 μm. Serum levels of ALT (**E**) and AST (**F**) in HFD-fed AAV-GFP and AAV-FBXO2 mice. qRT-PCR analysis of inflammatory (**G**) and fibrosis (**H**) genes expression in livers of HFD-fed AAV-GFP and AAV-FBXO2 mice. **I** A diagram showing the mechanism by which FBXO2 regulates adipogenesis and metabolic health of obese mice. The SCFFBXO2 complex catalyzes the phosphorylated insulin receptor by ubiquitination and inhibits hyperplasia and adipogenic differentiation of adipocyte precursor cells. Statistical data are presented as mean ± SEM of three independent experiments, and statistical analysis was performed using an unpaired *t*-test. *P < 0.05, **P < 0.01, ***P < *0.001*, *n* = 8.

To further study the role of FBXO2 overexpression in regulating adipogenesis and metabolic health. We next conducted gene overexpression with Adeno-Associated Virus (AAV)-mediated *Fbxo2* gene transfer into both sWAT and eWAT of mice. FBXO2 overexpression was validated in sWAT and eWAT, but not in the liver of mice (Fig. S10A). Our opinion is that short-term HFD rapidly initiates adipogenesis while long-term HFD drives existing adipocytes to hypertrophy for storage energy. The FBXO2-overexpression and control mice were subjected to a short- or long-term HFD feeding for 4 or 16 weeks. There was no discernible difference in body weight and fat mass between the two groups fed with short- or long-term HFD (Fig. S10B–E). Histological analysis revealed that adipocytes in sWAT from AAV-FBXO2 mice with larger size compared to controls with both short- (Fig. S10F) and long-term HFD feeding (Fig. 7A, B). Furthermore, FBXO2 overexpression inhibited the mRNA levels of adipocyte progenitor markers and the adipogenic genes in sWAT of both short- and long-term HFD feeding mice, while the apoptotic genes expression was comparable between the two groups (Figs. 7C and S10G). When under long-term HFD feeding, fibrosis and inflammation were obviously aggravated in sWAT of AAV-FBXO2 mice with Masson, F4/80 and α-SMA staining (Fig. 7A). This was further validated by higher expression of inflammatory and fibrotic genes (Fig. 7D, E). FBXO2-overexpression mice showed impaired glucose tolerance and insulin sensitivity compared with control by glucose tolerance test (GTT) and insulin tolerance test (ITT) in both short- (Fig. S10H, I) and long-term HFD feeding mice (Fig. 7F, G). Serum levels of TG, TC, FFA, and leptin were significantly higher in HFD-fed AAV-FBXO2 mice than those of controls, while adiponectin level was lower in AAV-FBXO2 mice (Fig. 7H–L). Further, we analyzed the biological function of eWAT and the liver caused by FBXO2 overexpression. Histological analysis showed that eWAT of AAV-FBXO2 mice with larger adipocyte size, aggravated fibrotic and inflammatory state with HE, Masson, F4/80 and α-SMA staining (Fig. 8A). Higher expression of inflammatory and fibrotic genes was found in eWAT of FBXO2 overexpression mice (Fig. 8B, C). In the liver, FBXO2 overexpression obviously aggravated fat infiltration, fibrosis and inflammation by analysis of HE, Masson, F4/80 and α-SMA staining (Fig. 8D). Serum levels of ALT and AST were increased, which indicated impaired liver function in FBXO2 overexpressed mice (Fig. 8E, F). Also, the expression of inflammatory and fibrotic genes was higher in the liver of FBXO2 overexpressed mice than in controls (Fig. 8G, H). Taken together, these data suggest that FBXO2 overexpression significantly inhibited adipogenesis and aggravated the metabolic health of HFD-induced obese mice.

## Discussion

The emergence of hyperplastic adipocytes in de novo differentiation is often accompanied by the attenuation of fibrosis and inflammation within white adipose tissue during obesity [33, 34]. There is a growing body of evidence suggesting that promoting adipogenesis contributes to the preservation of metabolic health and enhances glucose metabolism [35, 36]. Consequently, researchers are actively seeking novel regulators of adipogenesis to address obesity-related metabolic disorders. Notably, insulin serves as a central regulator of adipogenesis, with the insulin receptor (IR) playing a pivotal role in the insulin signaling pathway [37, 38]. Post-translational modification of IR, particularly ubiquitination, has been implicated in regulating insulin signal transduction. However, the precise relationship between IR post-translational modification and adipogenesis remains elusive. In our study, we demonstrate that FBXO2 serves as a potent repressor of adipogenesis both in vitro and in vivo, by catalyzing the ubiquitination modification and subsequent degradation of the phosphorylated IR. FBXO2 exerts this inhibitory effect by suppressing the proliferation of adipocyte progenitors, leading to aberrant adipocyte expansion and dysfunction. Consequently, FBXO2 promotes the onset of insulin resistance, fatty liver, and other obesity-related complications (Fig. 8I). Our findings offer a novel theoretical foundation for further understanding the mechanism of adipogenesis.

It is reported that FBXO2 plays a multifaceted role in various biological processes by targeting diverse substrates, including those involved in tumor progression, non-alcoholic fatty liver disease, and viral and bacterial infections [39–42]. In this study, we firstly propose the role of FBXO2 in regulating adipose plasticity, which is a braking mechanism of the insulin signaling pathway in adipogenesis. Given that the substrate of FBXO2 is always a phosphorylated protein [43], we sought to elucidate whether FBXO2 specifically targets the phosphorylated form of IR. As expected, our investigation revealed that FBXO2 could no longer ubiquitinate IR if the phospho-sites (Y1179 and Y1180) are mutant. This indicates that FBXO2 effectively and forcefully involves in regulating insulin signaling by directly targeting p-IR ubiquitination and degradation. In other studies, it has been documented that the SBD domain is essential for FBXO2 to recognize certain glycoprotein substrates [41]. Hence, we infer that FBXO2 catalyzes the ubiquitination of different substrate proteins via either the FBD or SBD domain in various physiological and pathological contexts. In this study, we elucidate the indispensable role of the F-box domain (FBD) for FBXO2 in catalyzing p-IR ubiquitination. In the future, screening for small-molecule compounds that target the FBD domain or p-IR ubiquitination could offer novel therapeutic strategies for promoting adipogenesis and ameliorating metabolic health in obesity.

Obesity often causes M1 macrophages infiltration and adipocyte progenitors differentiating into myofibroblasts expressing α–SMA in this pathological environment. This precipitates a decline in the plasticity of adipose tissue and leads to inflammation, fibrosis, progenitor cell senescence, and catecholamine resistance [44, 45]. The mechanisms governing adipose tissue expansion, including the proliferation of fat cells or the enlargement of existing fat cells, as well as their distribution (subcutaneous or visceral), profoundly influence metabolic health [46, 47]. Notably, the proliferative activity of adipocyte progenitors serves as a key determinant of adipose tissue plasticity, orchestrating critical adaptive processes such as adipose tissue expansion, browning, and the maintenance of the fat cell population [30, 48]. In mice, Fbxo2 deficiency led to an increased number of newly generated fat cells in sWAT, while its overexpression obviously inhibited adipocyte generation. In our study, we find that FBXO2 mainly regulates the plasticity of subcutaneous fat but not in visceral fat. We speculate that this might be related to the significantly higher sensitivity of subcutaneous fat to insulin compared to visceral fat [49]. Recent studies have shed further light on the benefits of adipogenesis in maintaining metabolic health, particularly in obese individuals. Our findings indicate that FBXO2 deficiency in adipose progenitors gives mice a metabolically healthy phenotype. Our work underscoring the role of FBXO2 as an inhibitor of adipogenesis and its potential use for regulating adipose plasticity and metabolic health.

In the animal studies, we constructed knockout models with three types of Cre mice according to the *Fbxo2* expression pattern during adipogenic differentiation. Pdgfra-Cre is the most commonly used for targeting adipocyte precursor cells in the study of adipogenesis. Fabp4-Cre is a classic tool for studying adipose development, while its lack of specificity in targeting limits its application. Adiponectin-Cre is a good choice for studying adipose function, which targets the mature adipocytes. We previously checked the knockdown efficiency of FBXO2 in adipose tissue and liver; the results showed that FBXO2 was significantly knocked down in adipose tissue of Pdgfra-Cre and Fabp4-Cre mice, while there was no effect in Adiponectin-Cre mice. This is consistent with the pattern of high expression of *Fbxo2* in adipocyte precursor cells and early adipogenic differentiation, while low expression in mature adipocytes. Pdgfra-Cre and Fabp4-Cre restrict the expression of *Fbxo2* during the initiation stage of adipogenesis, thereby exerting a subsequent effect of inhibiting adipogenesis. This is the main reason why we observed Pdgfra-Cre and Fabp4-Cre mice effects, while Adiponectin-Cre had no effects. In addition, *Fbxo2* is unchanged in the liver, so the overall phenotype of the mice we observed was mainly related to adipose tissue rather than the liver.

Together, our study identifies FBXO2 as an inhibitor of adipogenesis. FBXO2 targets the insulin receptor for ubiquitination based on its phosphorylation to brake insulin signaling in adipogenesis, both in vitro and in vivo. Gene deletion of Fbxo2 in mice promoted adipogenesis and improved remodeling of adipose tissue, thus ameliorating the overall metabolic health under the condition of excess nutrition. While Fbxo2 overexpression showed an opposite function in regulating adipogenesis and metabolic health. Our findings suggest that targeting insulin receptor ubiquitination catalyzed by FBXO2 may represent a novel therapeutic approach for improving metabolic health in obesity.

## Supplementary information

Original Data

Reproducibility checklist

Supplementary TableS1

Supplementary TableS2

Supplementary TableS3

Supplementary TableS4

Supplementary FigureS1

Supplementary FigureS2

Supplementary FigureS3

Supplementary FigureS4

Supplementary FigureS5

Supplementary FigureS6

Supplementary FigureS7

Supplementary FigureS8

Supplementary FigureS9

Supplementary FigureS10

Supplementary Figure legend

## Data Availability

All data needed to evaluate the conclusions in the paper are present in the paper and/or the Supplementary Materials.

## References

[1] Ghaben AL, Scherer PE. Adipogenesis and metabolic health. Nat Rev Mol Cell Biol. 2019;20:242–58. 10.1038/s41580-018-0093-z 30610207

[2] Sakers A, De Siqueira MK, Seale P, Villanueva CJ. Adipose-tissue plasticity in health and disease. Cell. 2022;185:419–46. 10.1016/j.cell.2021.12.016 PMC11152570 35120662

[3] Vishvanath L, Gupta RK. Contribution of adipogenesis to healthy adipose tissue expansion in obesity. J Clin Invest. 2019;129:4022–31. 10.1172/JCI129191 PMC6763245 31573549

[4] Heinonen S, Jokinen R, Rissanen A, Pietilainen KH. White adipose tissue mitochondrial metabolism in health and in obesity. Obes Rev. 2020;21:e12958. 10.1111/obr.12958 31777187

[5] Nunn ER, Shinde AB, Zaganjor E. Weighing in on adipogenesis. Front Physiol. 2022;13:821278. 10.3389/fphys.2022.821278 PMC8914022 35283790

[6] Wang QA, Scherer PE, Gupta RK. Improved methodologies for the study of adipose biology: insights gained and opportunities ahead. J Lipid Res. 2014;55:605–24. 10.1194/jlr.R046441 PMC3966696 24532650

[7] Le TKC, Dao XD, Nguyen DV, Luu DH, Bui TMH, Le TH, et al. Insulin signaling and its application. Front Endocrinol. 2023;14:1226655. 10.3389/fendo.2023.1226655 PMC10469844 37664840

[8] Green H, Kehinde O. An established preadipose cell line and its differentiation in culture. II. Factors affecting the adipose conversion. Cell. 1975;5:19–27. 10.1016/0092-8674(75)90087-2 165899

[9] Kolodziej M, Strauss S, Lazaridis A, Bucan V, Kuhbier JW, Vogt PM, et al. Influence of glucose and insulin in human adipogenic differentiation models with adipose-derived stem cells. Adipocyte. 2019;8:254–64. 10.1080/21623945.2019.1636626 PMC6768274 31280651

[10] Haeusler RA, McGraw TE, Accili D. Biochemical and cellular properties of insulin receptor signalling. Nat Rev Mol Cell Biol. 2018;19:31–44. 10.1038/nrm.2017.89 PMC5894887 28974775

[11] Garten A, Schuster S, Kiess W. The insulin-like growth factors in adipogenesis and obesity. Endocrinol Metab Clin North Am. 2012;41:283–95. v-vi. 10.1016/j.ecl.2012.04.011 22682631

[12] Saltiel AR, Kahn CR. Insulin signalling and the regulation of glucose and lipid metabolism. Nature. 2001;414:799–806. 10.1038/414799a 11742412

[13] Rahman MS, Hossain KS, Das S, Kundu S, Adegoke EO, Rahman MA, et al. Role of insulin in health and disease: an update. Int J Mol Sci. 2021;22:12. 10.3390/ijms22126403 PMC8232639 34203830

[14] Balaji V, Pokrzywa W, Hoppe T. Ubiquitylation pathways in insulin signaling and organismal homeostasis. Bioessays. 2018;40:e1700223. 10.1002/bies.201700223 29611634

[15] Tawo R, Pokrzywa W, Kevei E, Akyuz ME, Balaji V, Adrian S, et al. The ubiquitin ligase CHIP integrates proteostasis and aging by regulation of insulin receptor turnover. Cell. 2017;169:470–482.e413. 10.1016/j.cell.2017.04.003 PMC5406386 28431247

[16] Chen C, Gu L, Matye DJ, Clayton YD, Hasan MN, Wang Y, et al. Cullin neddylation inhibitor attenuates hyperglycemia by enhancing hepatic insulin signaling through insulin receptor substrate stabilization. Proc Natl Acad Sci USA. 2022;119:6. 10.1073/pnas.2111737119 PMC8832973 35115401

[17] Li Y, Li S, Wu H. Ubiquitination-proteasome system (UPS) and autophagy are two main protein degradation machineries in response to cell stress. Cells 2022;11:851. 10.3390/cells11050851 PMC8909305 35269473

[18] Toma-Fukai S, Shimizu T. Structural diversity of ubiquitin E3 ligase. Molecules 2021;26:6682. 10.3390/molecules26216682 PMC8586995 34771091

[19] Zheng N, Shabek N. Ubiquitin ligases: structure, function, and regulation. Annu Rev Biochem. 2017;86:129–57. 10.1146/annurev-biochem-060815-014922 28375744

[20] Deshaies RJ, Joazeiro CA. RING domain E3 ubiquitin ligases. Annu Rev Biochem. 2009;78:399–434. 10.1146/annurev.biochem.78.101807.093809 19489725

[21] Nguyen KM, Busino L. The biology of F-box proteins: the SCF family of E3 ubiquitin ligases. Adv Exp Med Biol. 2020;1217:111–22. 10.1007/978-981-15-1025-0_8 31898225

[22] Wang Q, Li H, Tajima K, Verkerke ARP, Taxin ZH, Hou Z, et al. Post-translational control of beige fat biogenesis by PRDM16 stabilization. Nature. 2022;609:151–8. 10.1038/s41586-022-05067-4 PMC9433319 35978186

[23] Zhou N, Qi H, Liu J, Zhang G, Liu J, Liu N, et al. Deubiquitinase OTUD3 regulates metabolism homeostasis in response to nutritional stresses. Cell Metab. 2022;34:1023–1041.e1028. 10.1016/j.cmet.2022.05.005 35675826

[24] Li P, Song Y, Zan W, Qin L, Han S, Jiang B, et al. Lack of CUL4B in adipocytes promotes PPARgamma-mediated adipose tissue expansion and insulin sensitivity. Diabetes. 2017;66:300–13. 10.2337/db16-0743 27899484

[25] Baghaie L, Bunsick DA, Szewczuk MR. Insulin receptor signaling in health and disease. Biomolecules. 2023;13:5. 10.3390/biom13050807 PMC10216010 37238677

[26] Chang E, Kim CY. Natural products and obesity: a focus on the regulation of mitotic clonal expansion during adipogenesis. Molecules 2019;24:1157. 10.3390/molecules24061157 PMC6471203 30909556

[27] Fajas L. Adipogenesis: a cross-talk between cell proliferation and cell differentiation. Ann Med. 2003;35:79–85. 10.1080/07853890310009999 12795337

[28] Cinti S. Adipose organ development and remodeling. Compr Physiol. 2018;8:1357–431. 10.1002/cphy.c170042 30215863

[29] Scheja L, Heeren J. Metabolic interplay between white, beige, brown adipocytes and the liver. J Hepatol. 2016;64:1176–86. 10.1016/j.jhep.2016.01.025 26829204

[30] Auger C, Kajimura S. Adipose tissue remodeling in pathophysiology. Annu Rev Pathol. 2023;18:71–93. 10.1146/annurev-pathol-042220-023633 PMC9877135 36070562

[31] Singh R, Barrios A, Dirakvand G, Pervin S. Human brown adipose tissue and metabolic health: potential for therapeutic avenues. Cells. 2021;10:11. 10.3390/cells10113030 PMC8616549 34831253

[32] Xue S, Lee D, Berry DC. Thermogenic adipose tissue in energy regulation and metabolic health. Front Endocrinol. 2023;14:1150059. 10.3389/fendo.2023.1150059 PMC10067564 37020585

[33] Sun K, Kusminski CM, Scherer PE. Adipose tissue remodeling and obesity. J Clin Investig. 2011;121:2094–101. 10.1172/JCI45887 PMC3104761 21633177

[34] An SM, Cho SH, Yoon JC. Adipose tissue and metabolic health. Diab Metab J. 2023;47:595–611. 10.4093/dmj.2023.0011 PMC10555533 37482656

[35] Lundgren M, Svensson M, Lindmark S, Renstrom F, Ruge T, Eriksson JW. Fat cell enlargement is an independent marker of insulin resistance and ‘hyperleptinaemia. Diabetologia. 2007;50:625–33. 10.1007/s00125-006-0572-1 17216279

[36] Zhao GN, Tian ZW, Tian T, Zhu ZP, Zhao WJ, Tian H, et al. TMBIM1 is an inhibitor of adipogenesis and its depletion promotes adipocyte hyperplasia and improves obesity-related metabolic disease. Cell Metab. 2021;33:1640–54.e1648. 10.1016/j.cmet.2021.05.014 34107313

[37] Liao Z, Zhang C, Ding L, Moyers JS, Tang JX, Beals JM. Comprehensive insulin receptor phosphorylation dynamics profiled by mass spectrometry. FEBS J. 2022;289:2657–71. 10.1111/febs.16299 34826178

[38] Choi E, Bai XC. The Activation Mechanism of the Insulin Receptor: a Structural Perspective. Annu Rev Biochem. 2023;92:247–72. 10.1146/annurev-biochem-052521-033250 PMC10398885 37001136

[39] Buehler M, Yi X, Ge W, Blattmann P, Rushing E, Reifenberger G, et al. Quantitative proteomic landscapes of primary and recurrent glioblastoma reveal a protumorigeneic role for FBXO2-dependent glioma-microenvironment interactions. Neuro Oncol. 2023;25:290–302. 10.1093/neuonc/noac169 PMC9925714 35802605

[40] Ji J, Shen J, Xu Y, Xie M, Qian Q, Qiu T, et al. FBXO2 targets glycosylated SUN2 for ubiquitination and degradation to promote ovarian cancer development. Cell Death Dis. 2022;13:442. 10.1038/s41419-022-04892-9 PMC9079088 35525855

[41] Yamada A, Hikichi M, Nozawa T, Nakagawa I. FBXO2/SCF ubiquitin ligase complex directs xenophagy through recognizing bacterial surface glycan. EMBO Rep. 2021;22:e52584. 10.15252/embr.202152584 PMC8567282 34515398

[42] Liu B, Lu H, Li D, Xiong X, Gao L, Wu Z, et al. Aberrant expression of FBXO2 disrupts glucose homeostasis through ubiquitin-mediated degradation of insulin receptor in obese mice. Diabetes. 2017;66:689–98. 10.2337/db16-1104 27932386

[43] Zhao X, Guo W, Zou L, Hu B. FBXO2 modulates STAT3 signaling to regulate proliferation and tumorigenicity of osteosarcoma cells. Cancer Cell Int. 2020;20:245. 10.1186/s12935-020-01326-4 PMC7296666 32549792

[44] Bluher M. Obesity: global epidemiology and pathogenesis. Nat Rev Endocrinol. 2019;15:288–98. 10.1038/s41574-019-0176-8 30814686

[45] Hammarstedt A, Gogg S, Hedjazifar S, Nerstedt A, Smith U. Impaired adipogenesis and dysfunctional adipose tissue in human hypertrophic obesity. Physiol Rev. 2018;98:1911–41. 10.1152/physrev.00034.2017 30067159

[46] Gustafson B, Gogg S, Hedjazifar S, Jenndahl L, Hammarstedt A, Smith U. Inflammation and impaired adipogenesis in hypertrophic obesity in man. Am J Physiol Endocrinol Metab. 2009;297:E999–E1003. 10.1152/ajpendo.00377.2009 19622783

[47] Zubiria MG, Vidal-Bravo J, Spinedi E, Giovambattista A. Relationship between impaired adipogenesis of retroperitoneal adipose tissue and hypertrophic obesity: role of endogenous glucocorticoid excess. J Cell Mol Med. 2014;18:1549–61. 10.1111/jcmm.12308 PMC4190901 24913911

[48] Yang Loureiro Z, Solivan-Rivera J, Corvera S. Adipocyte heterogeneity underlying adipose tissue functions. Endocrinology. 2022;163:1. 10.1210/endocr/bqab138 PMC8660558 34223880

[49] Merrick D, Sakers A, Irgebay Z, Okada C, Calvert C, Morley MP, et al. Identification of a mesenchymal progenitor cell hierarchy in adipose tissue. Science. 2019;364:6438. 10.1126/science.aav2501 PMC6816238 31023895

