# Supplementary material for: E3 ligase FBXO2-mediated protein stability of insulin receptor regulates adipogenesis and metabolic health in obesity
Source: Cell Death Dis. 2026 Jul 8;17(1):848. doi: 10.1038/s41419-026-09096-z (PMC13631264; doi:10.1038/s41419-026-09096-z)

Fig. 1A

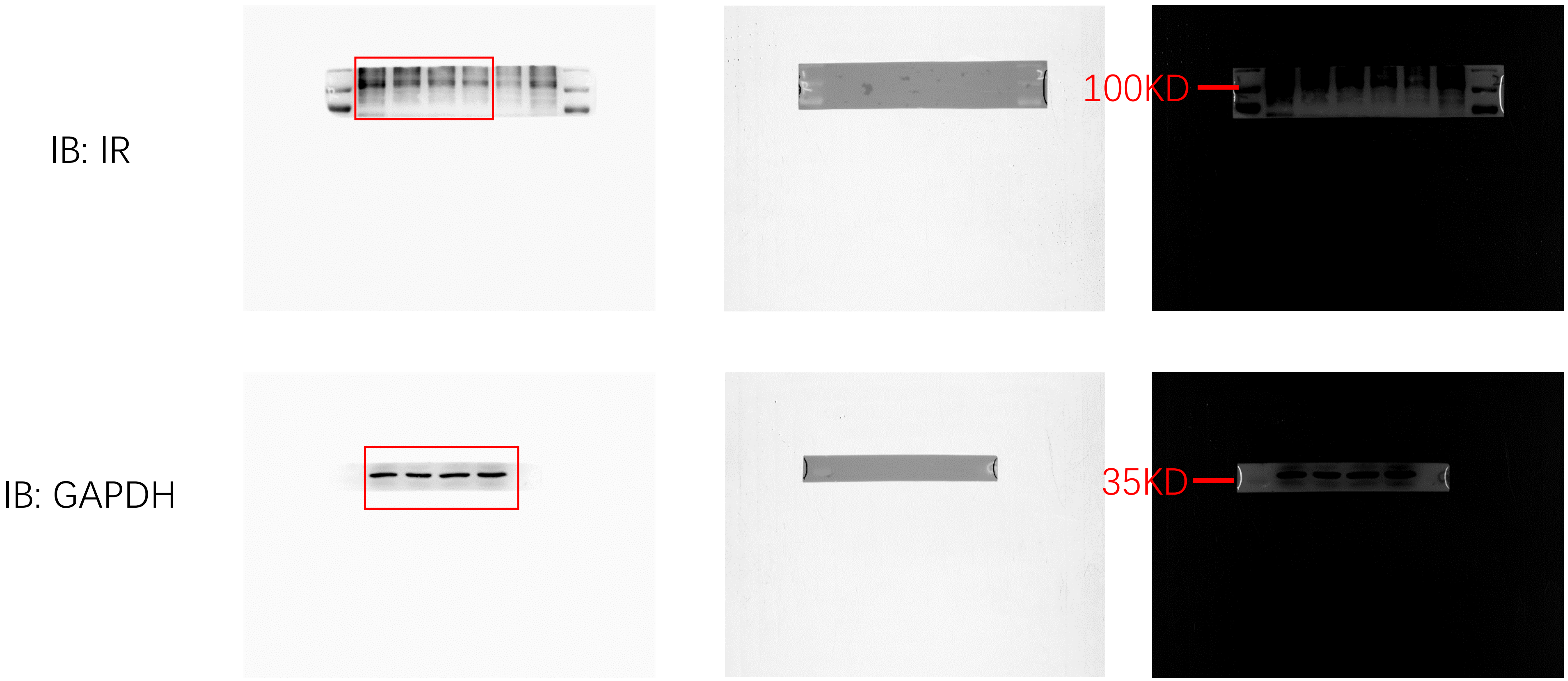

Fig. 1C

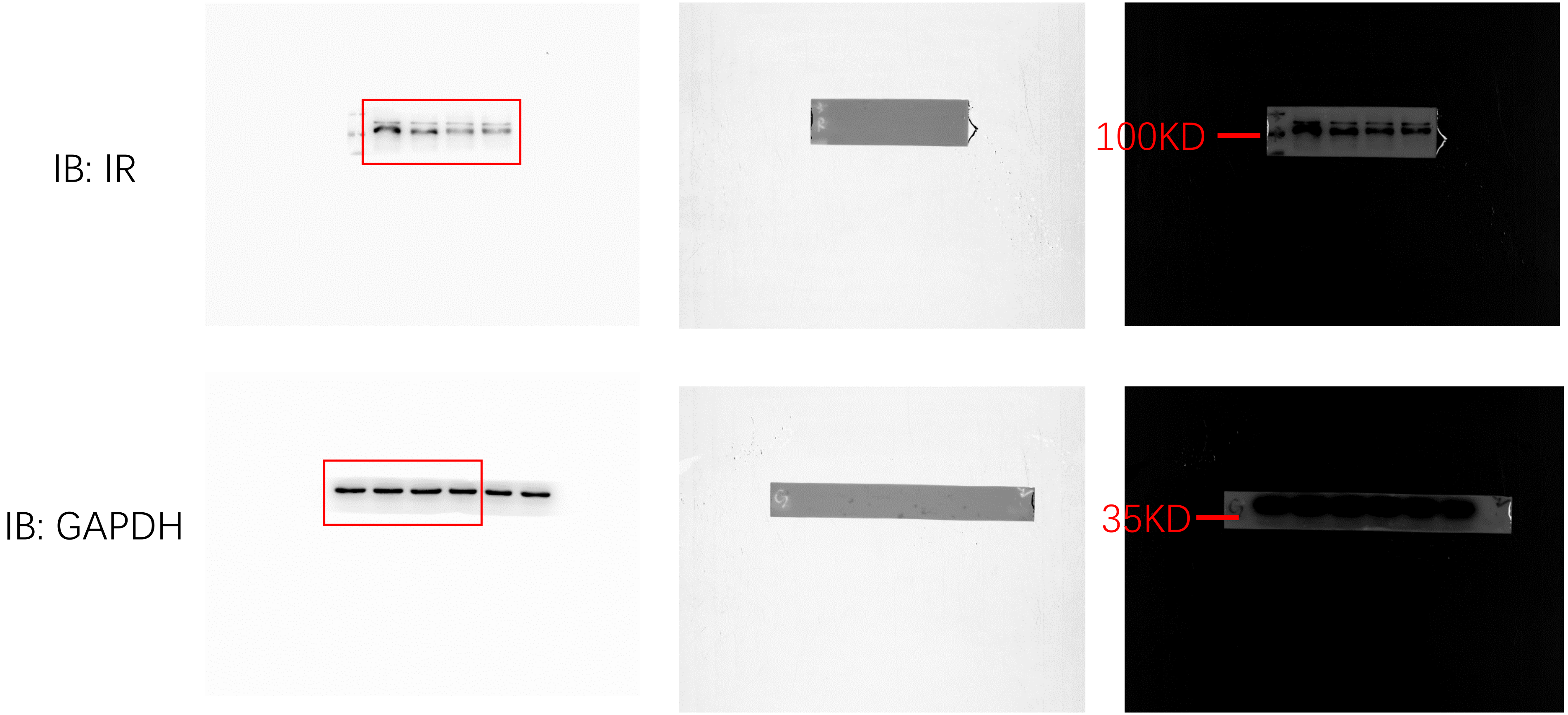

Fig. 1D

IB: p-IR

IB: IR

IB: GAPDH

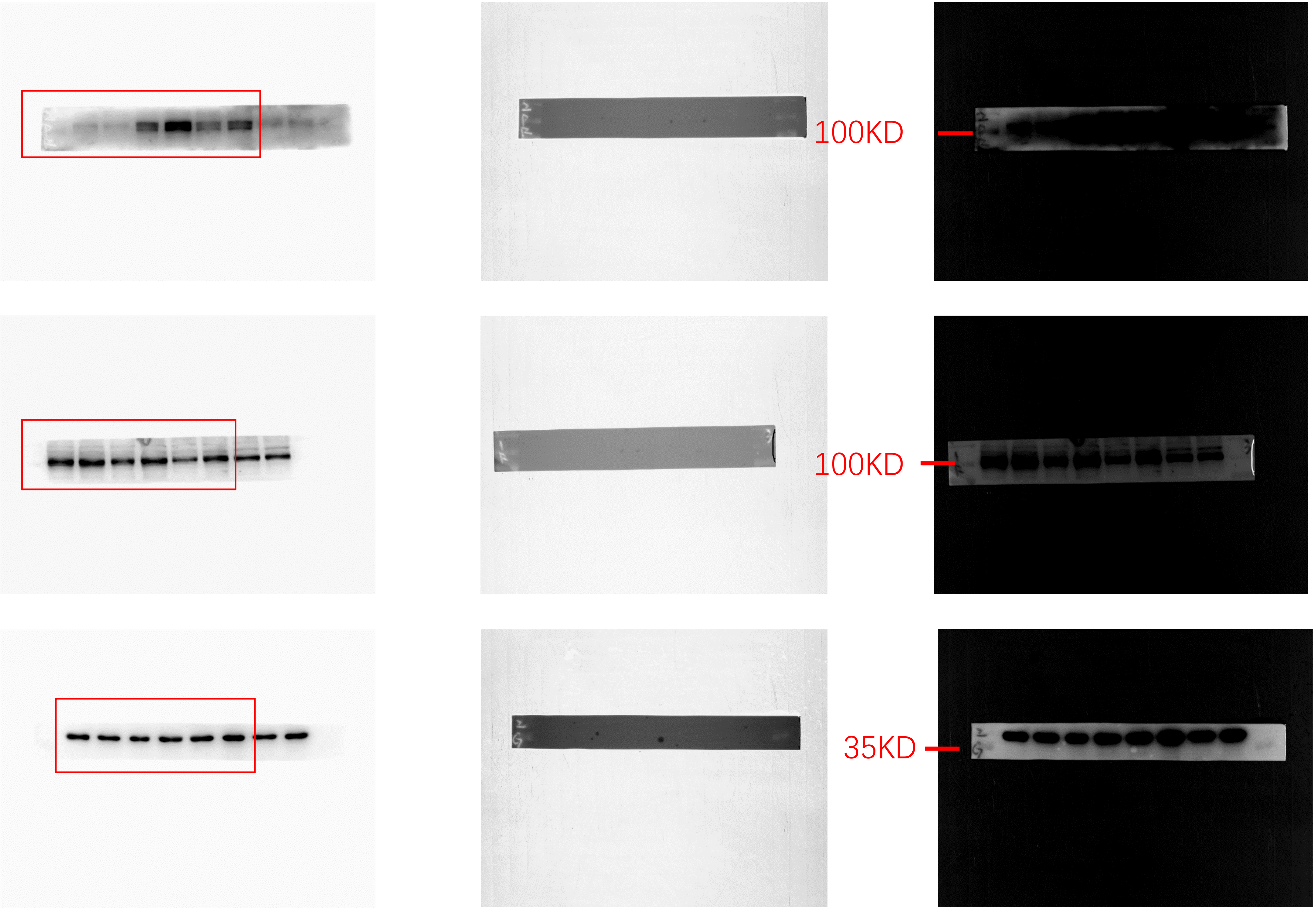

Fig. 1E

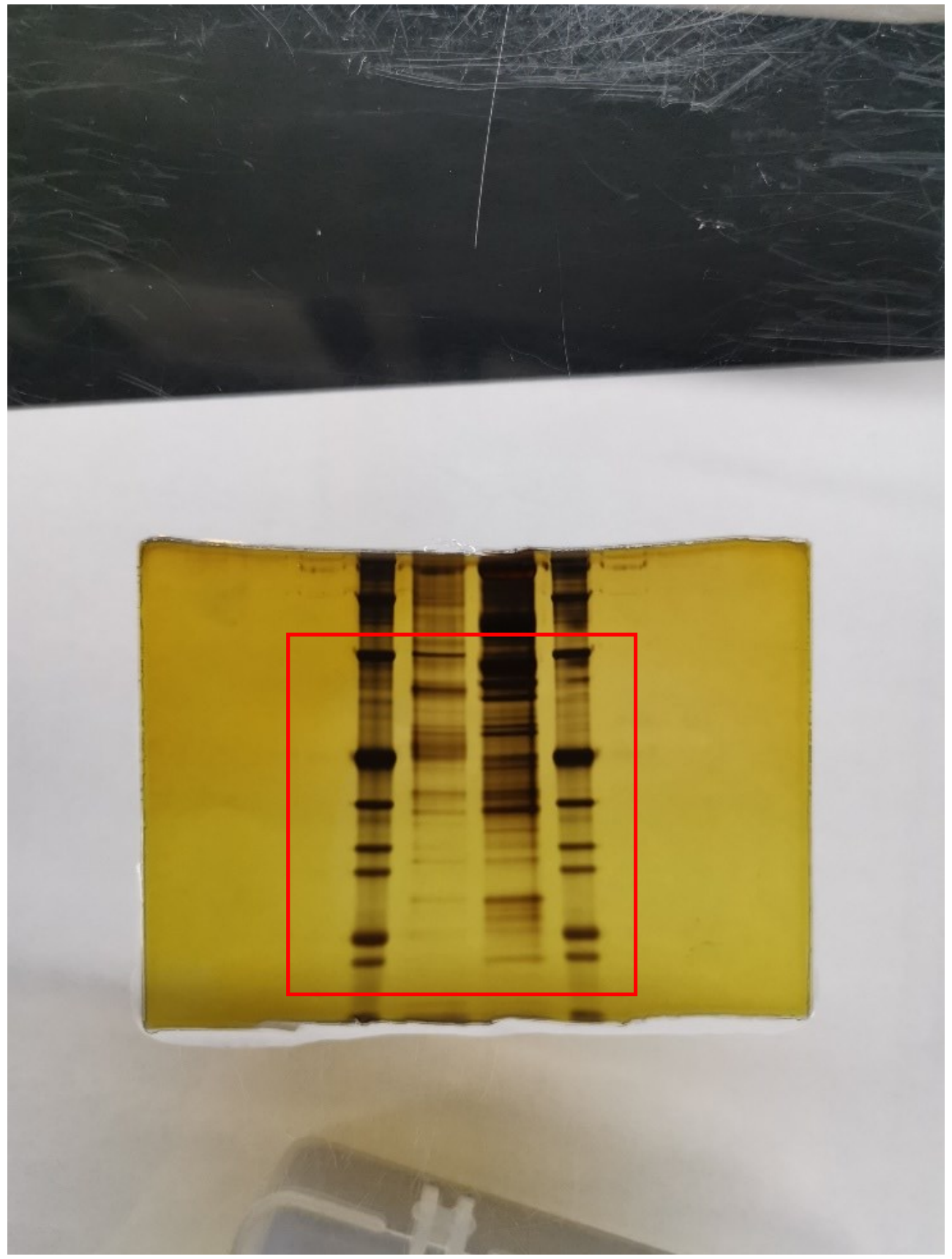

Fig. 1F

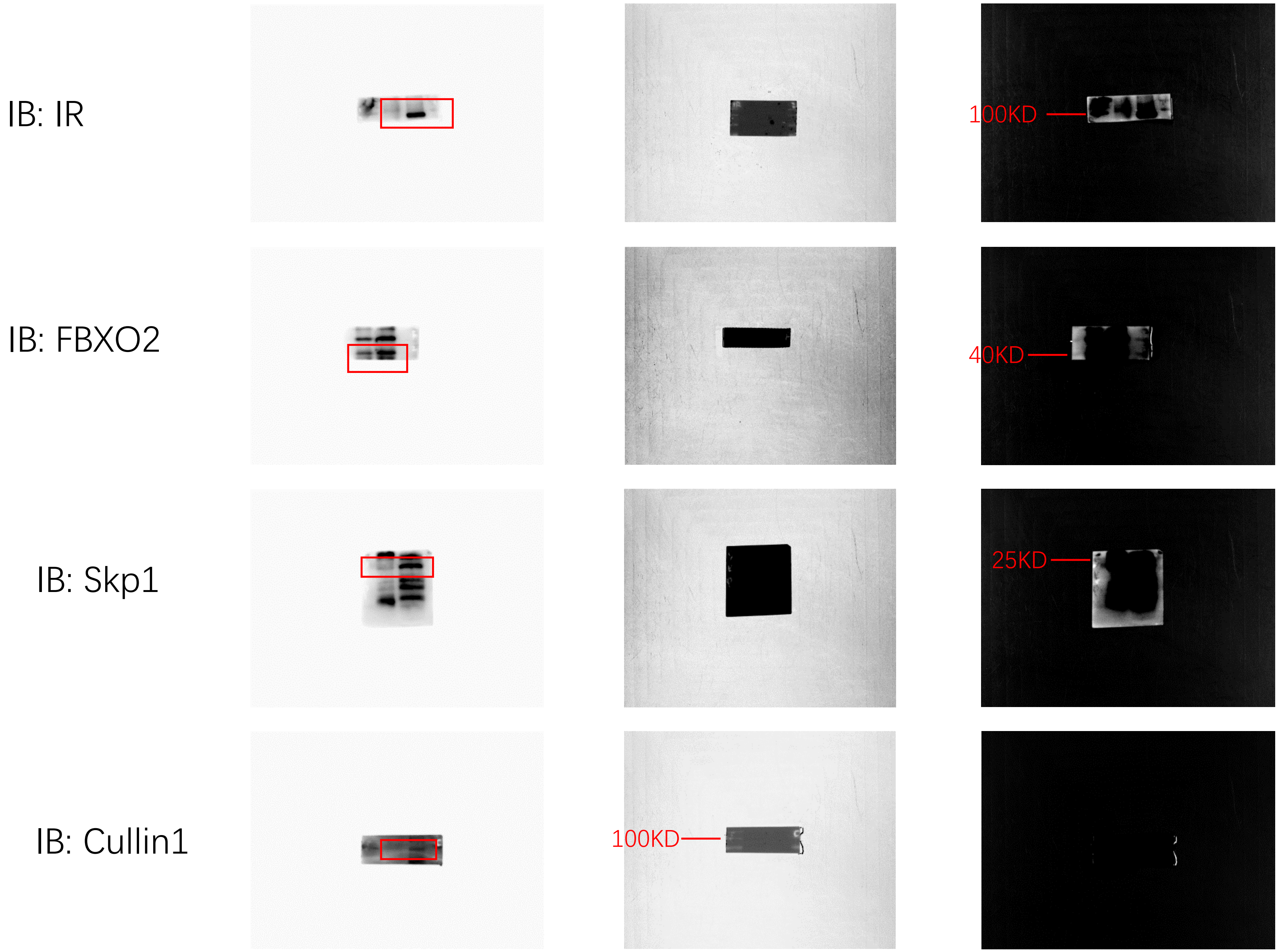

Fig. 1F

Input IB: Cullin1

Input IB: Skp1

Input IB: IR

Input IB: FBXO2

Input IB: GAPDH

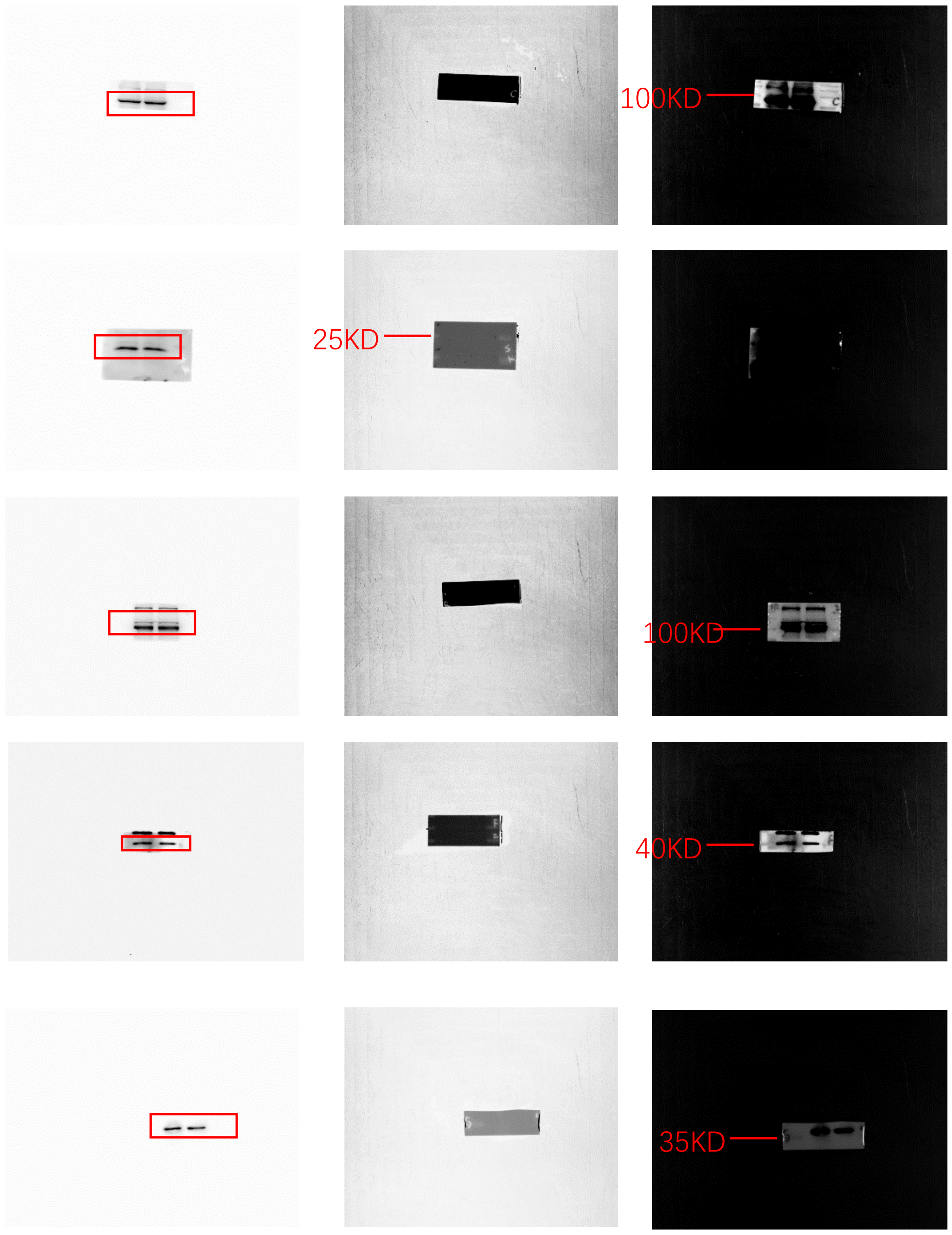

Fig. 1G

IB: IR

IB: FBXO2

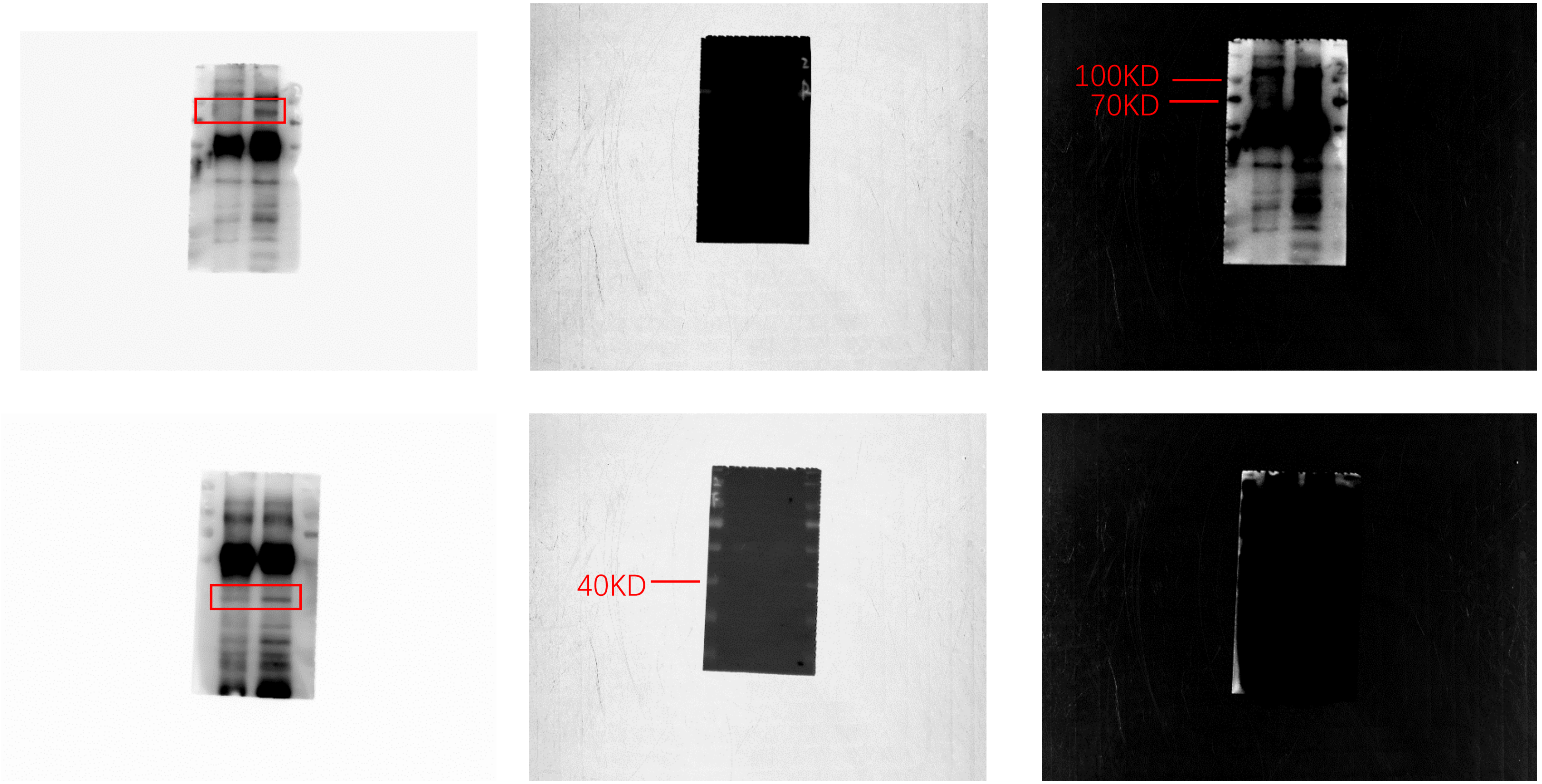

Fig. 1G

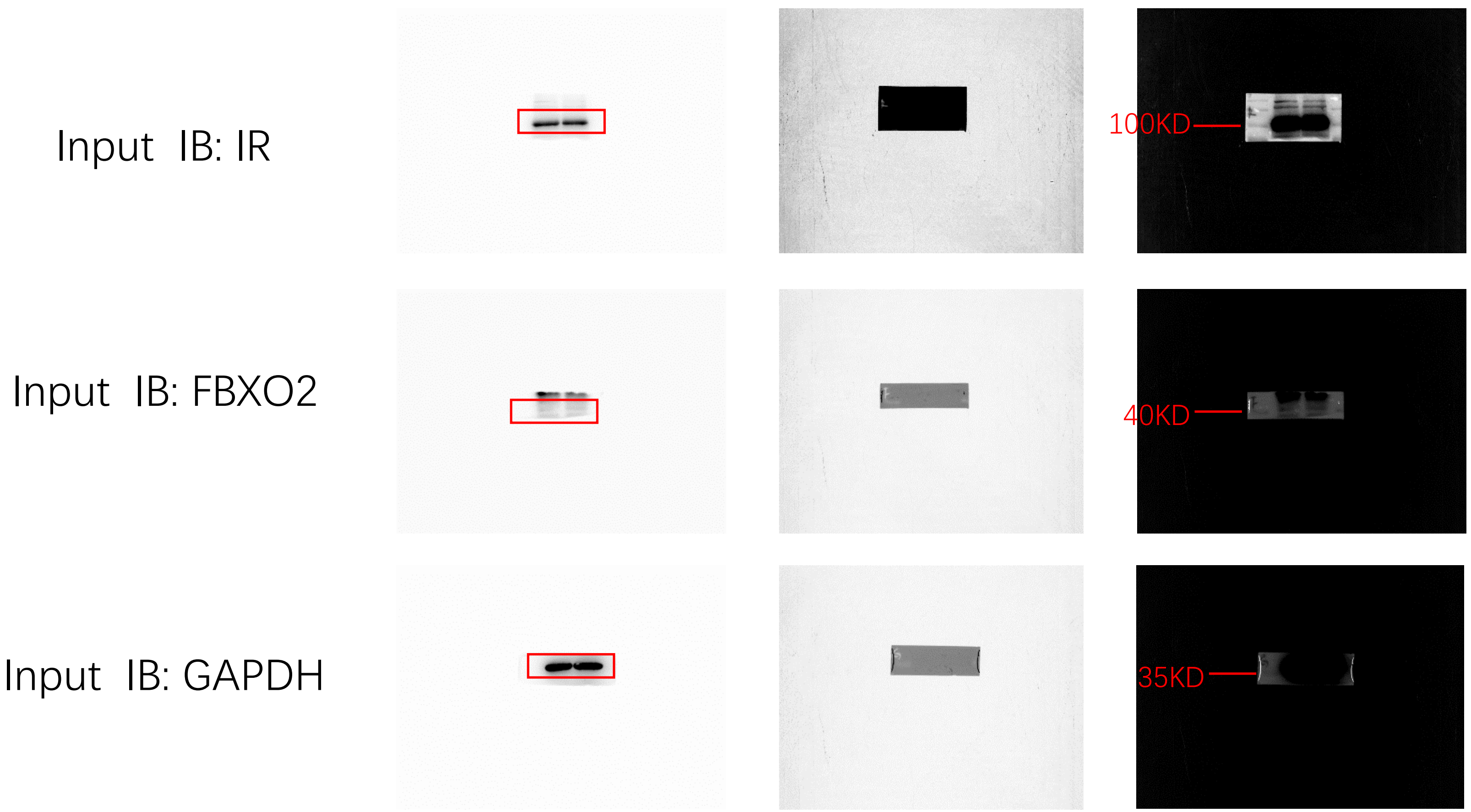

Fig. 1H

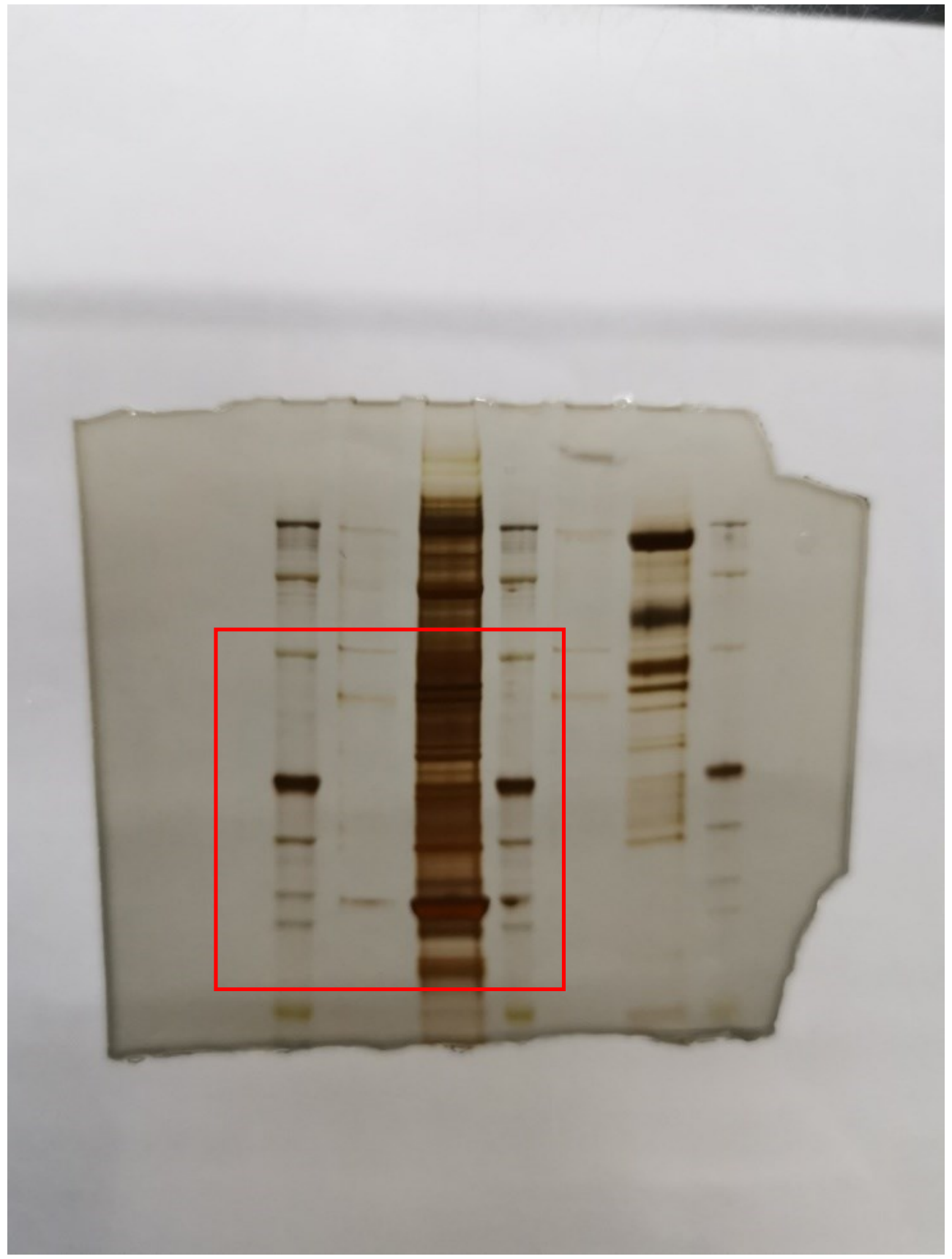

Fig. 1I

IB: p-IR

IB: IR

IB: p-IRS1

IB: IRS1

IB: p-AKT （T308）

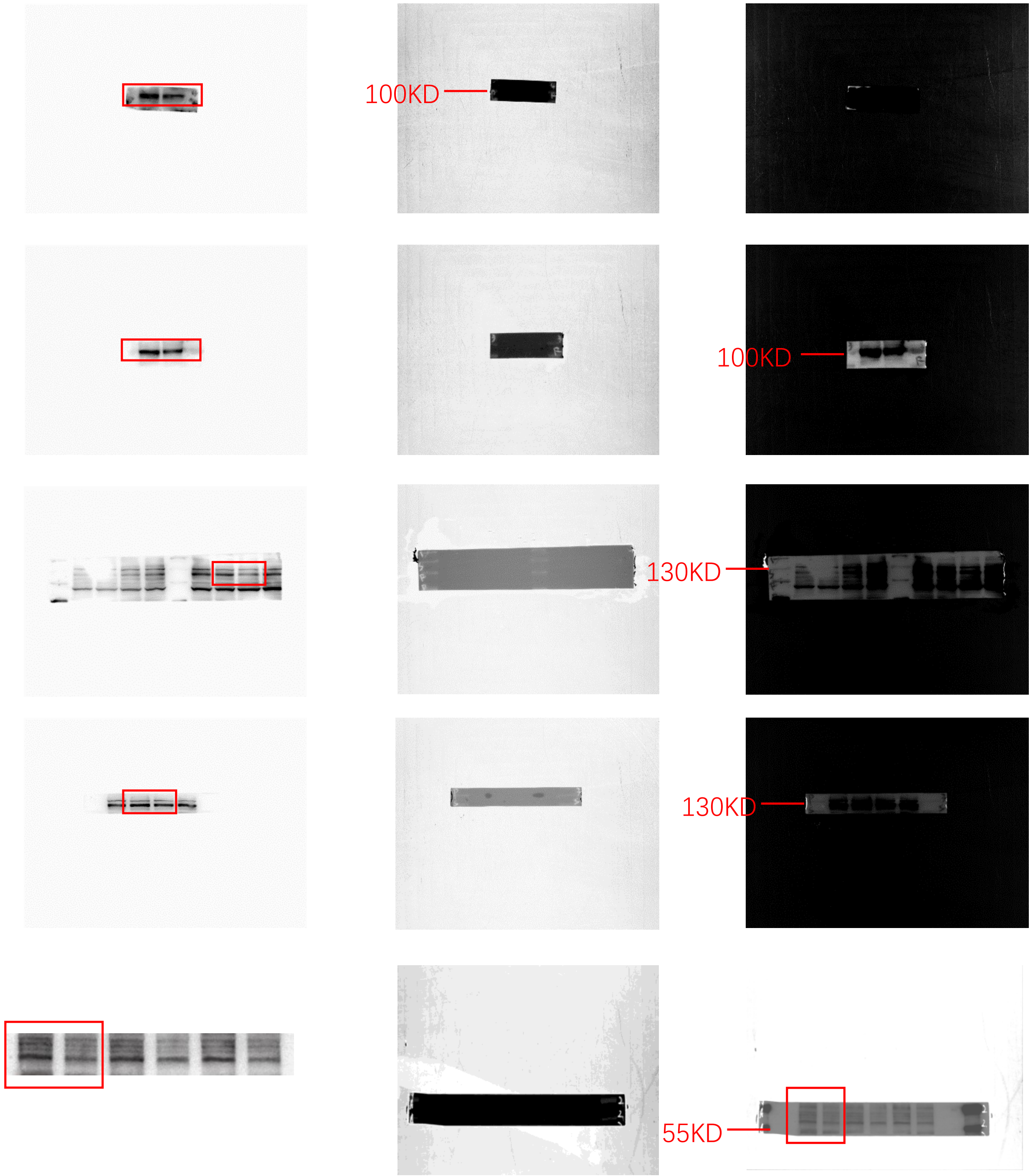

Fig. 1I

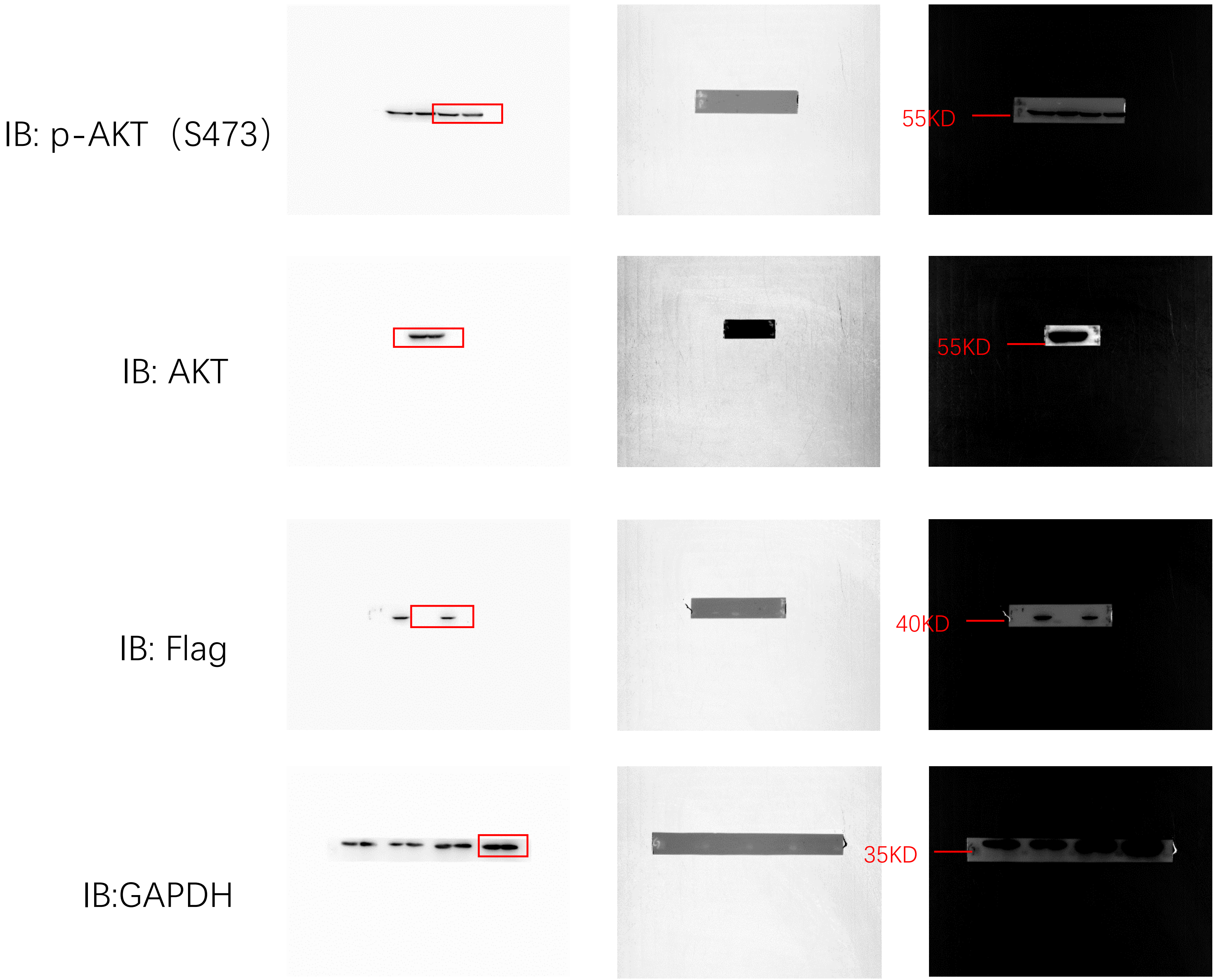

Fig. 1J

IB: p-IR

IB: IR

IB: Flag

IB:GAPDH

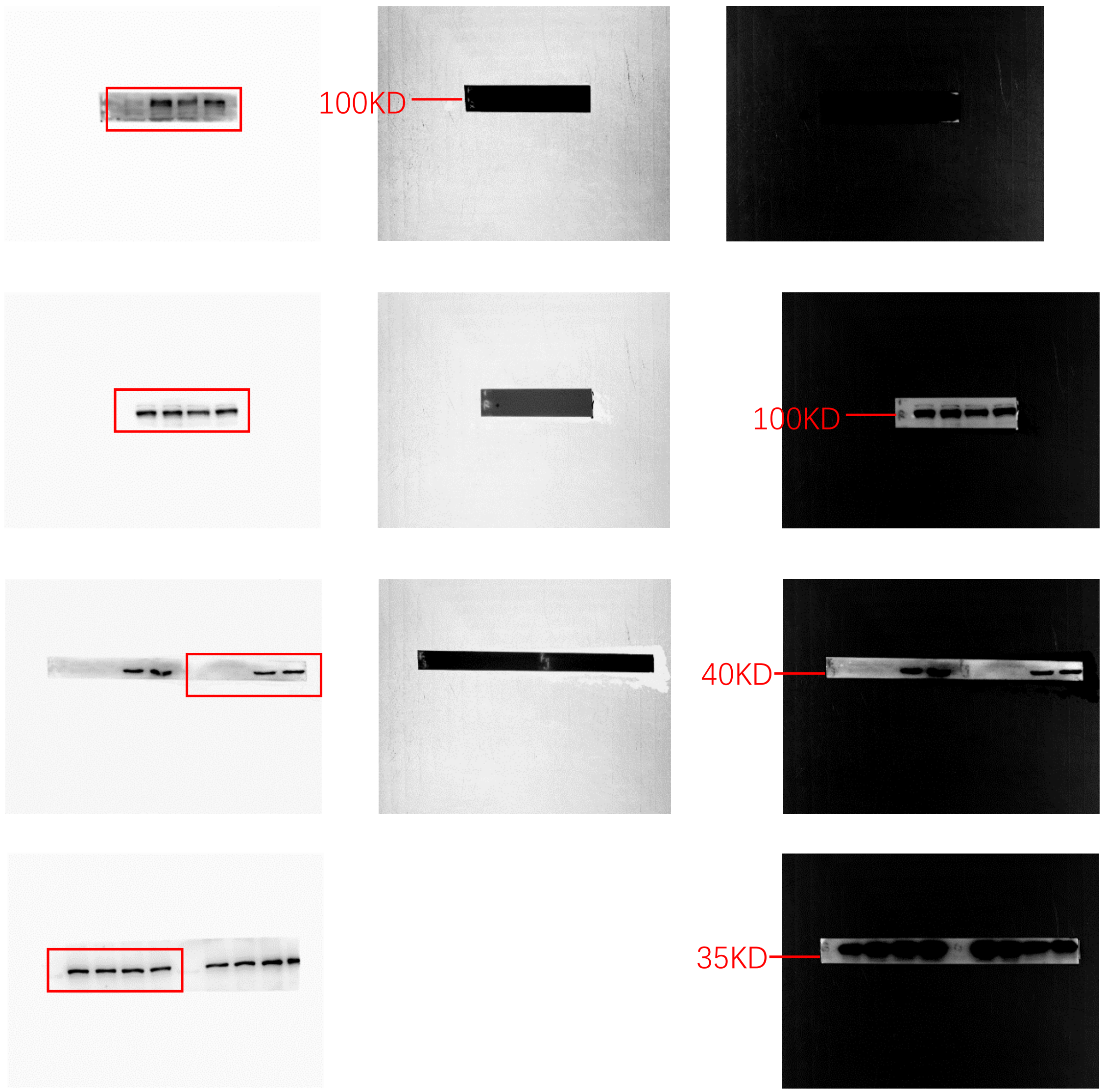

Fig. 2A

IB:HA

Input IB:HA

Input IB:Myc

Input IB:Flag

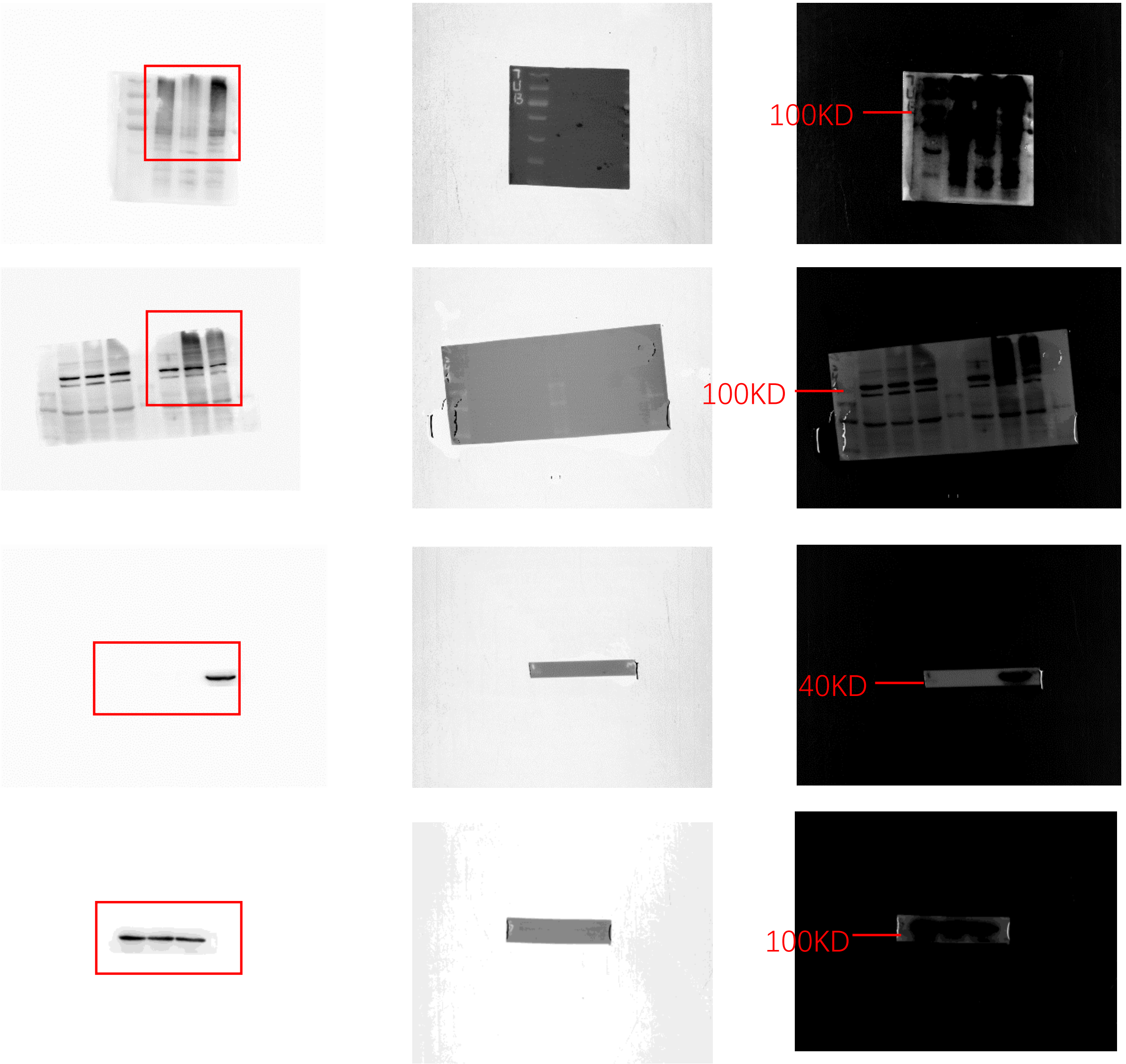

Fig. 2B

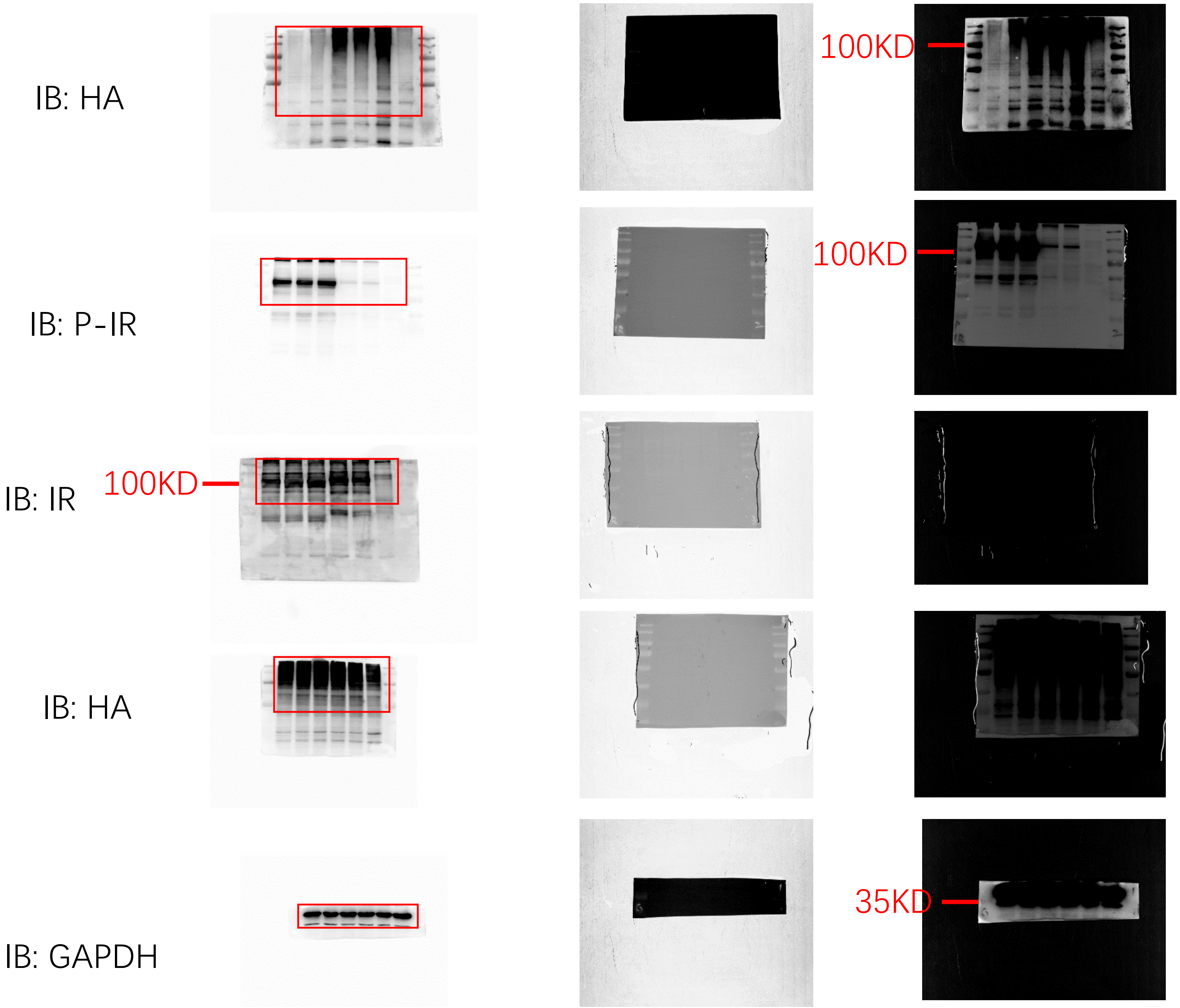

Fig. 2C

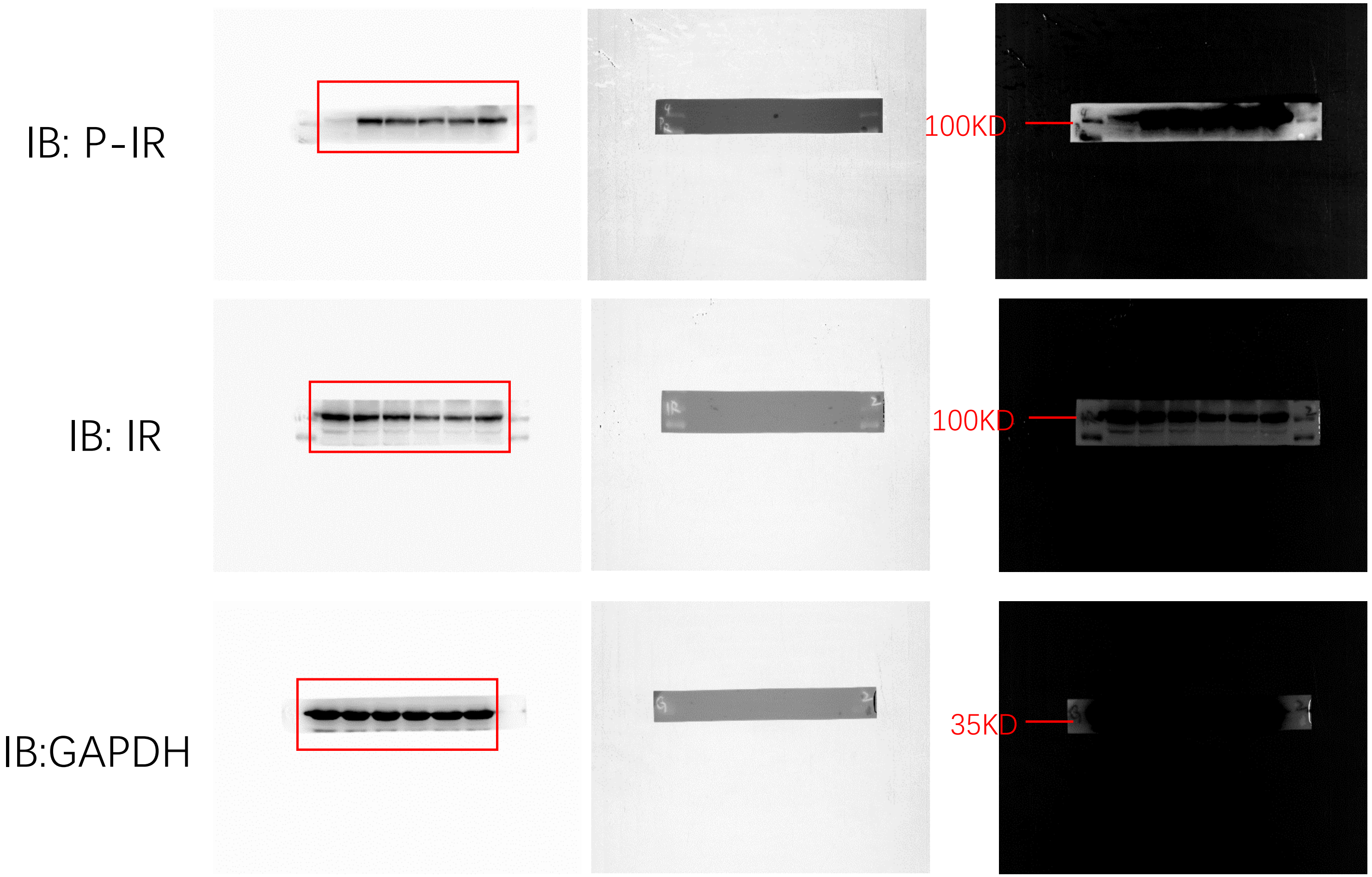

Fig. 2D

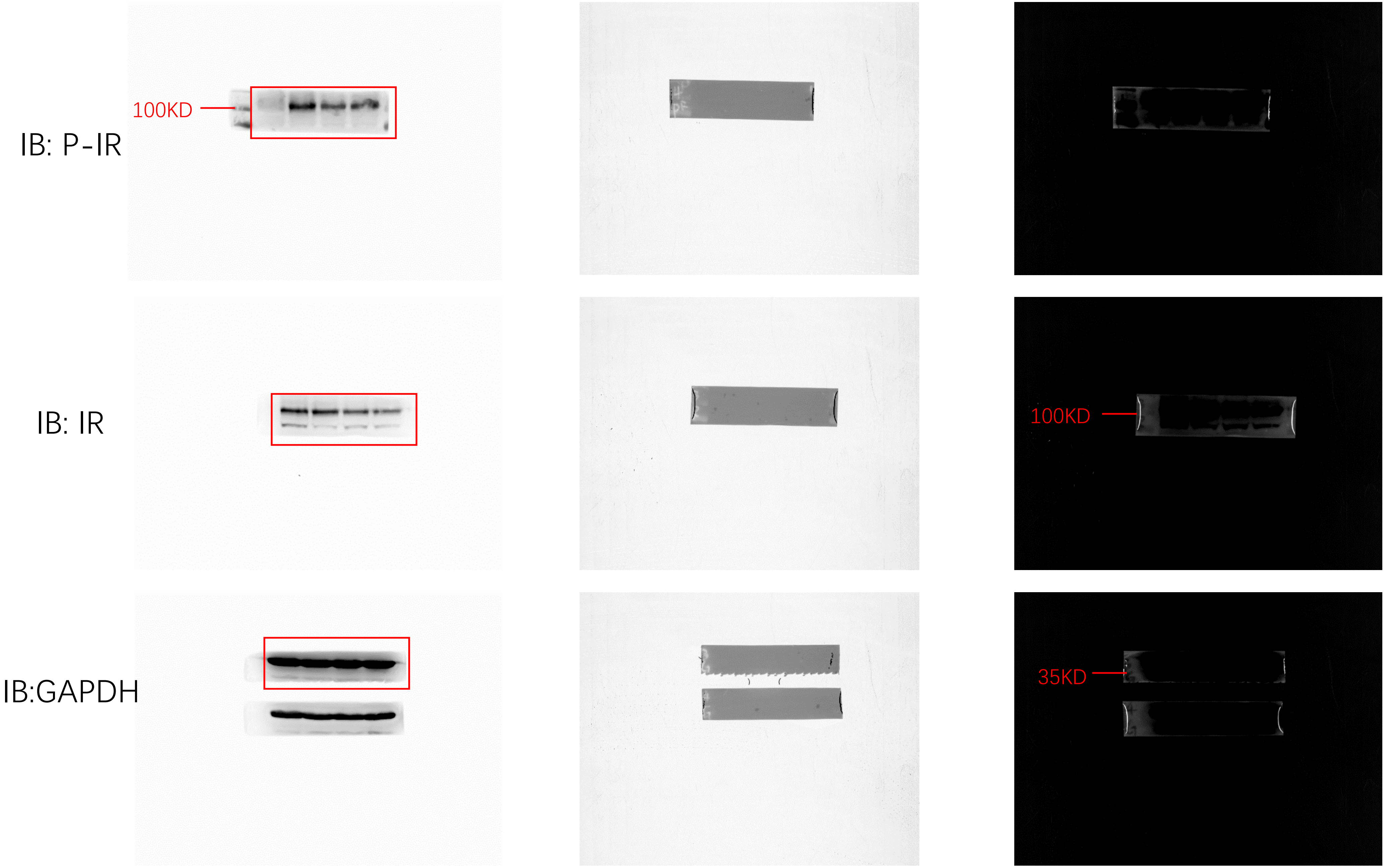

Fig. 2G

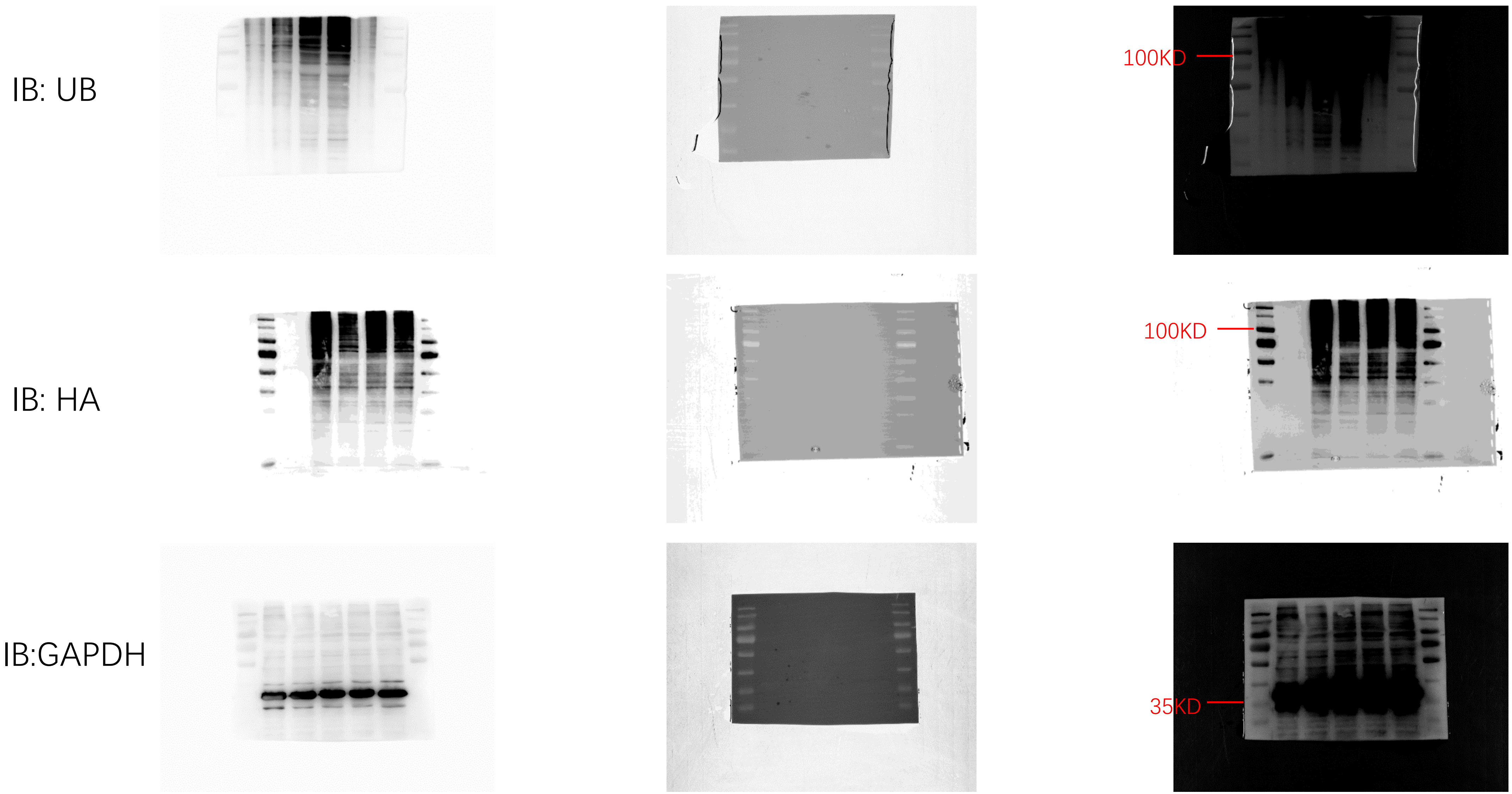

Fig. 3B

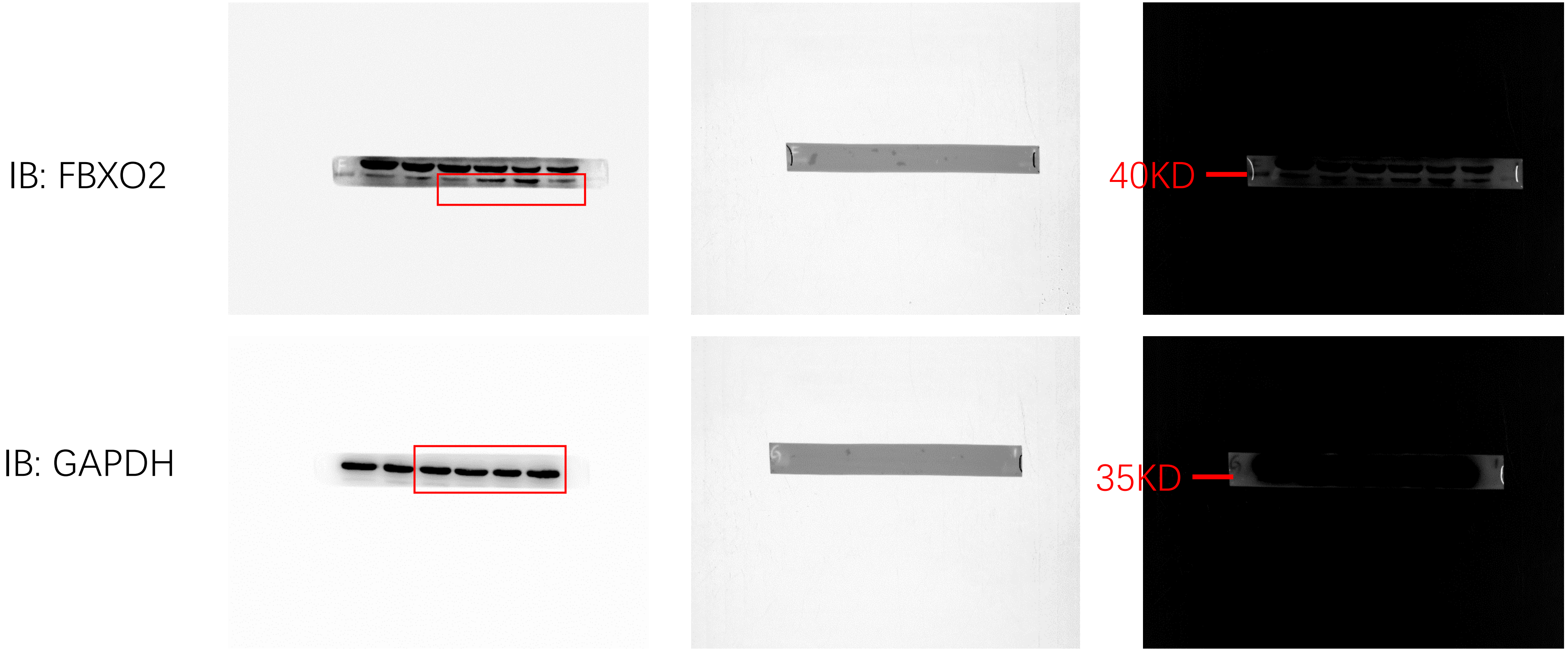

Fig. 4B

IB:C/EBPβ

IB:KROX20

IB:C/EBPα

IB:PPARγ

IB:FBXO2

IB: GAPDH

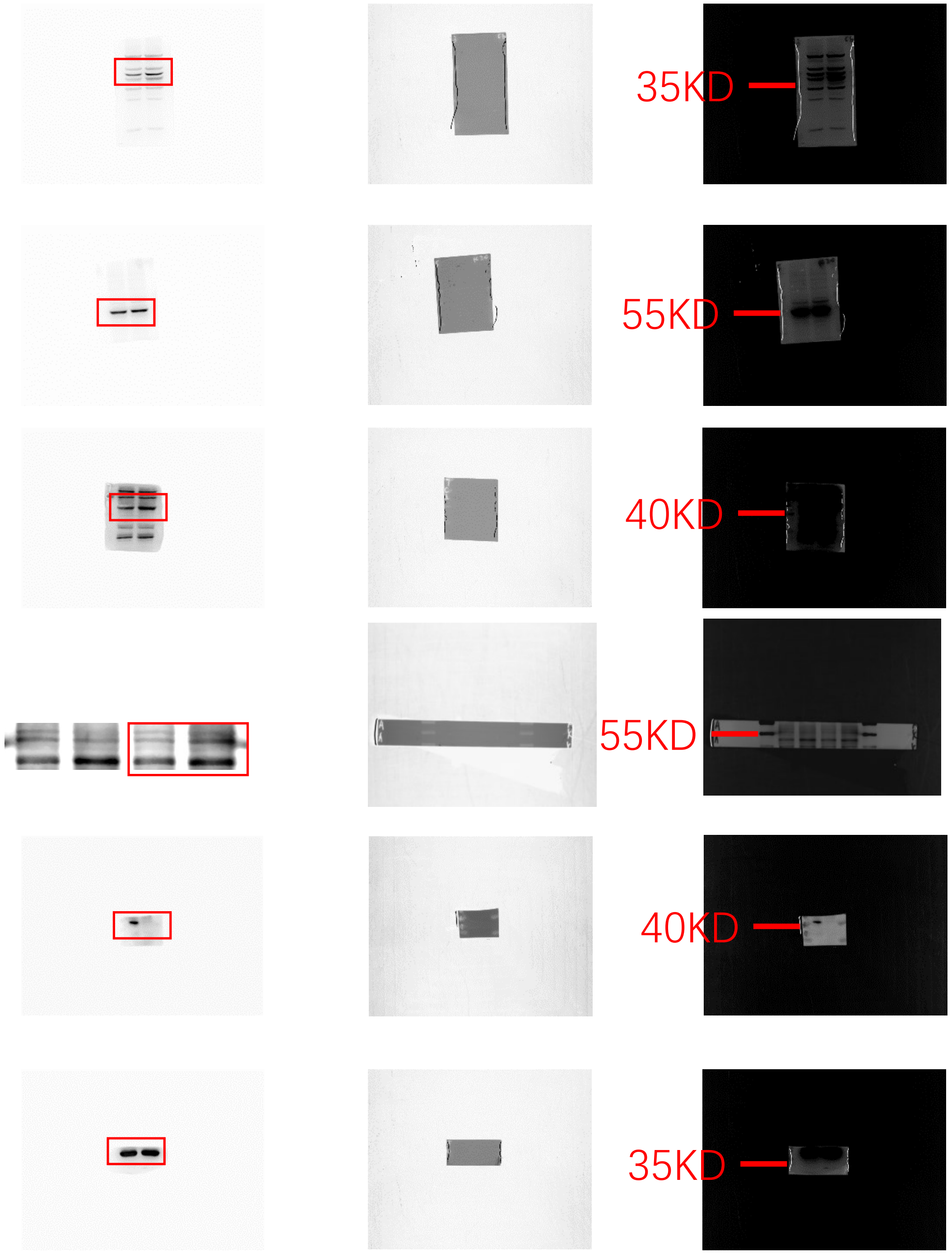

Fig. 4F

IB:C/EBPβ

IB:KROX20

IB:C/EBPα

IB:PPARγ

IB:FBXO2

IB: GAPDH

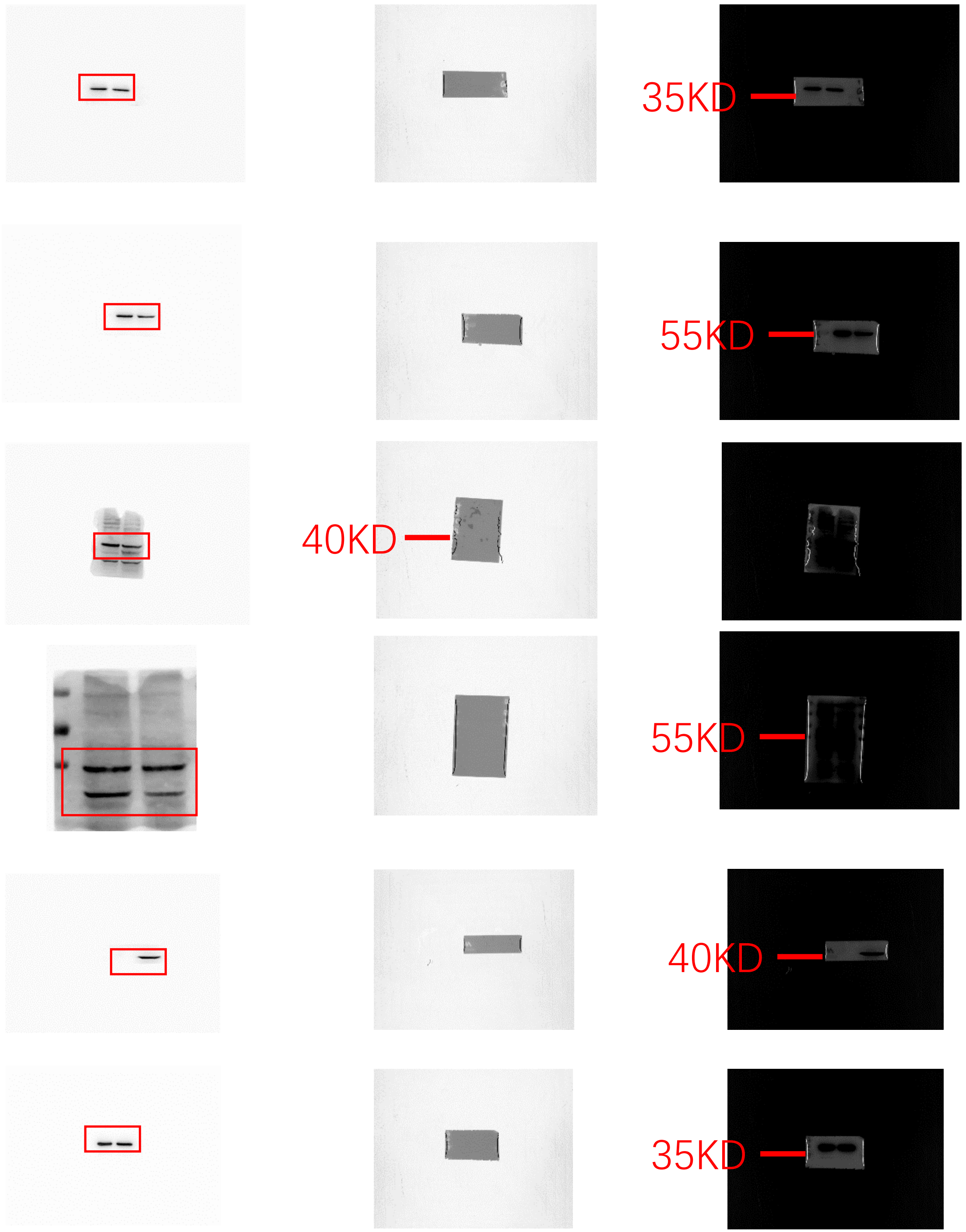

Fig. 4I

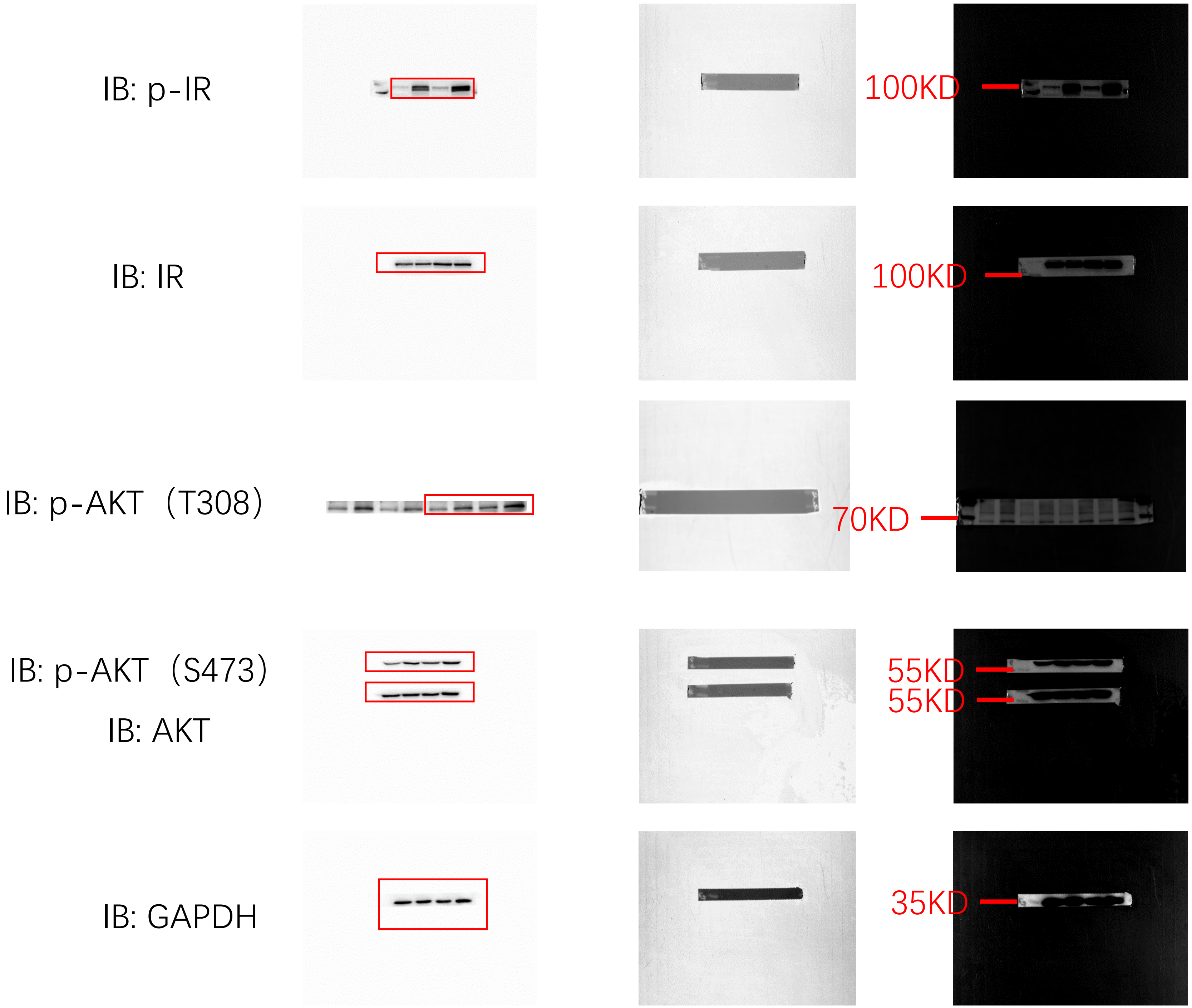
IB: p-IR
IB: IR
IB: p-AKT（T308）
IB: p-AKT（S473）
IB: AKT
IB: GAPDH
100KD
100KD
70KD
55KD
55KD
35KD

Fig. 4L

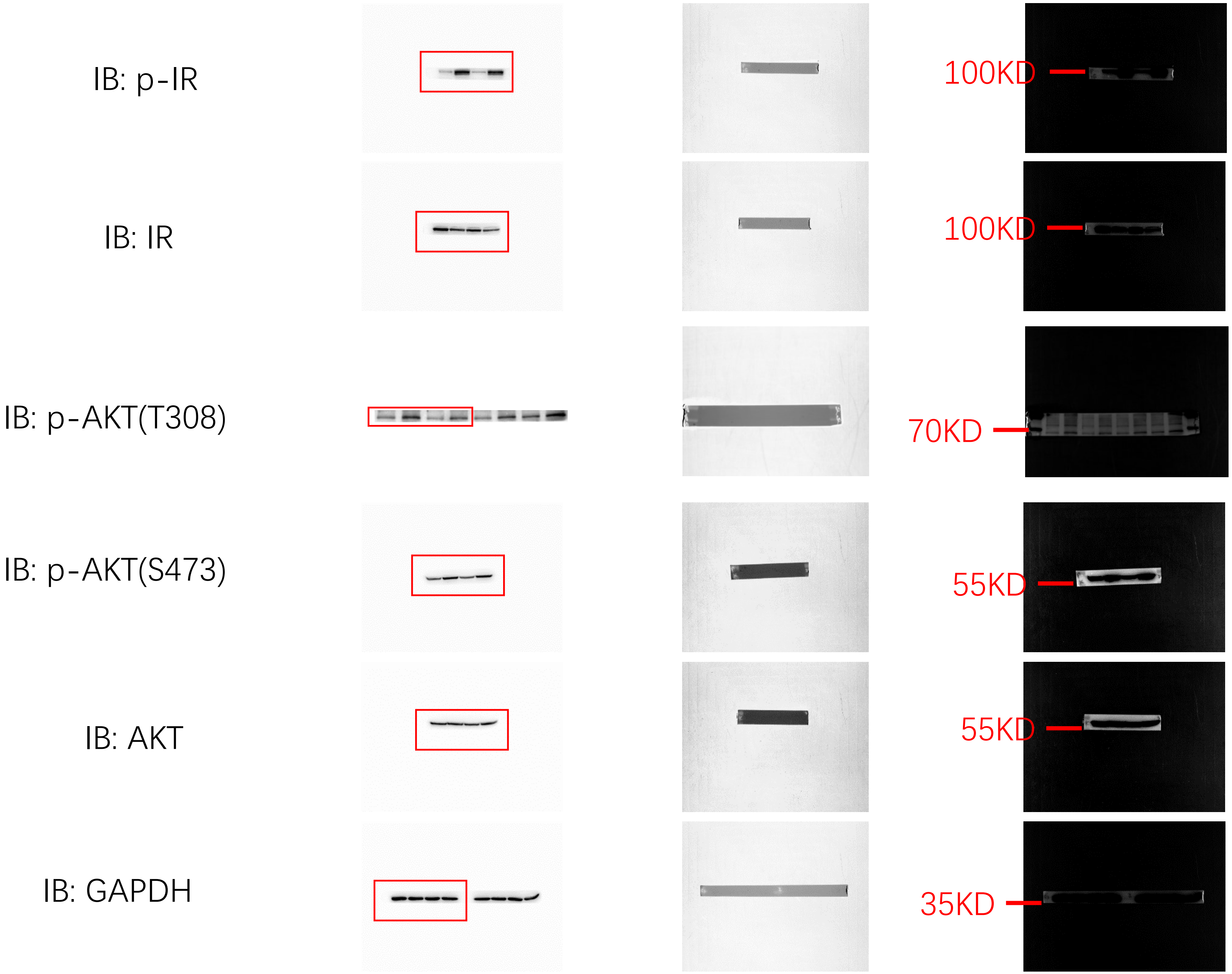

IB: p-IR
IB: IR
IB: p-AKT(T308)
IB: p-AKT(S473)
IB: AKT
IB: GAPDH
100KD
100KD
70KD
55KD
55KD
35KD

Fig. 5E

IB: p-IR

IB: IR

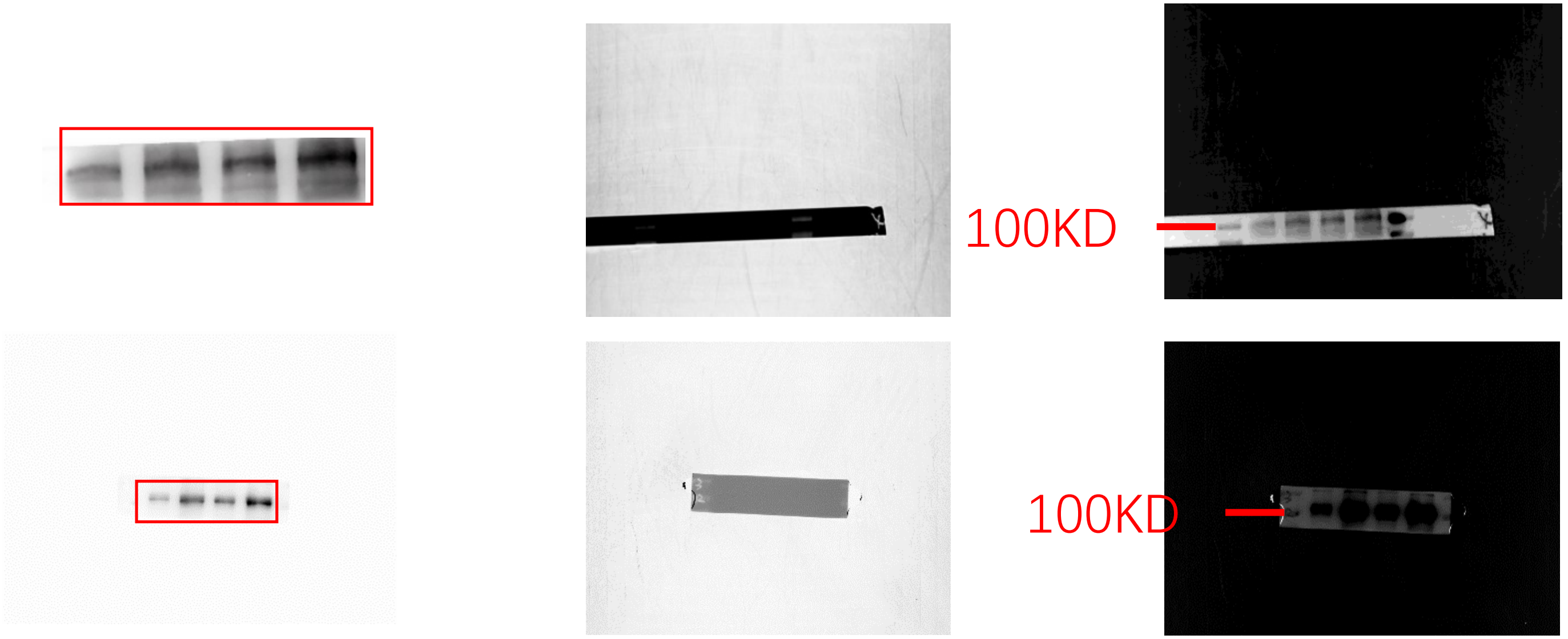

Fig. 5E

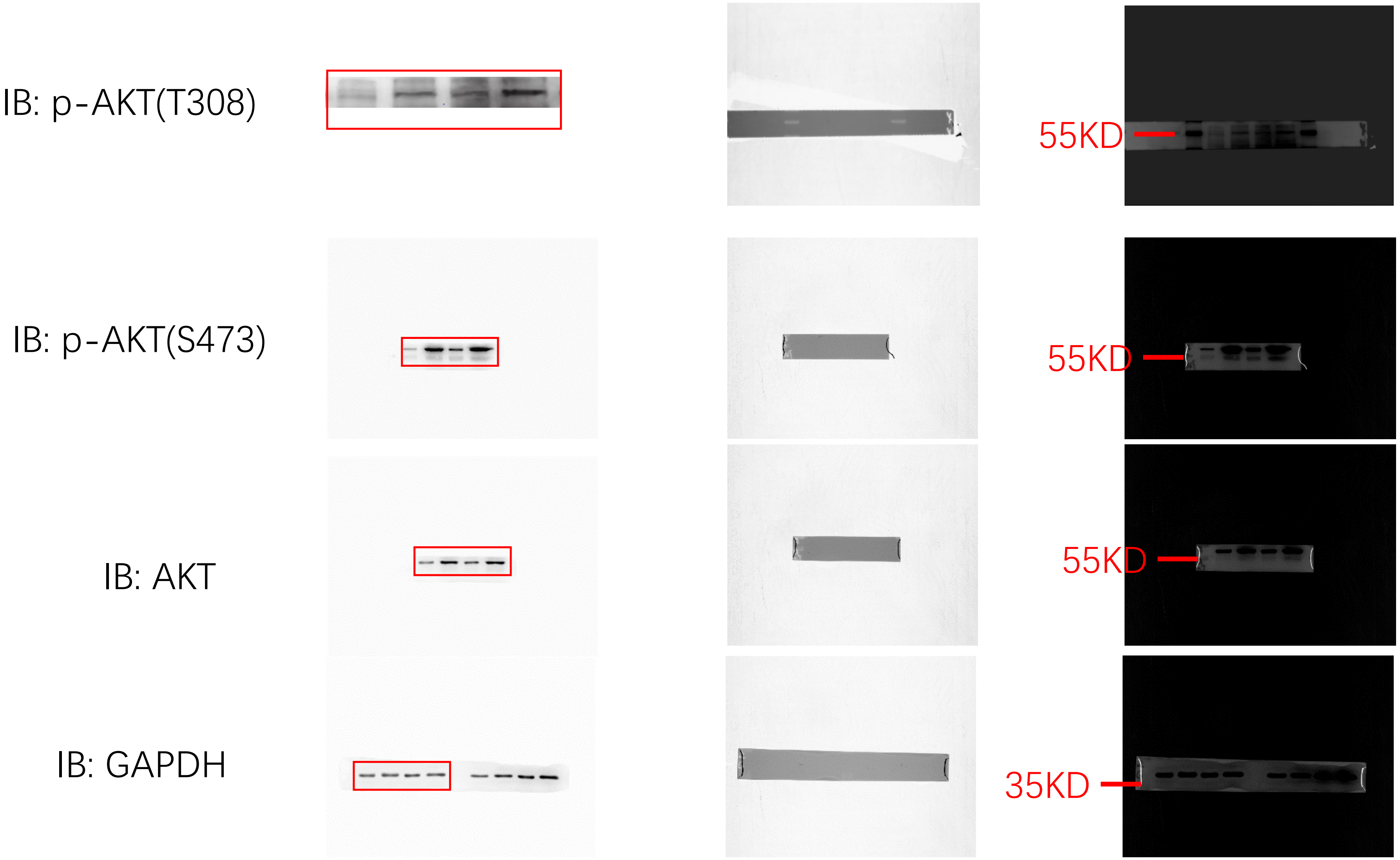

Supplementary Fig. 1A

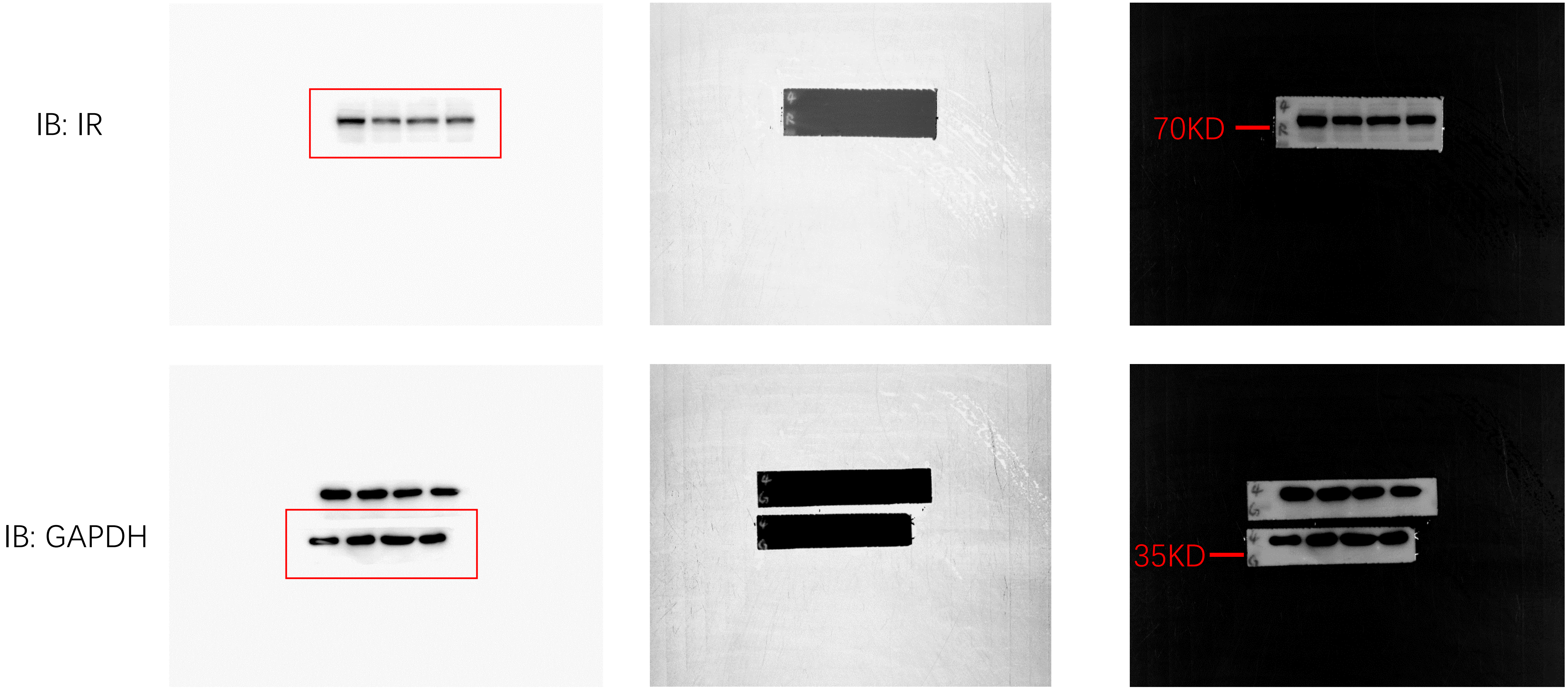

Supplementary Fig. 1C

IB: IR

IB: GAPDH

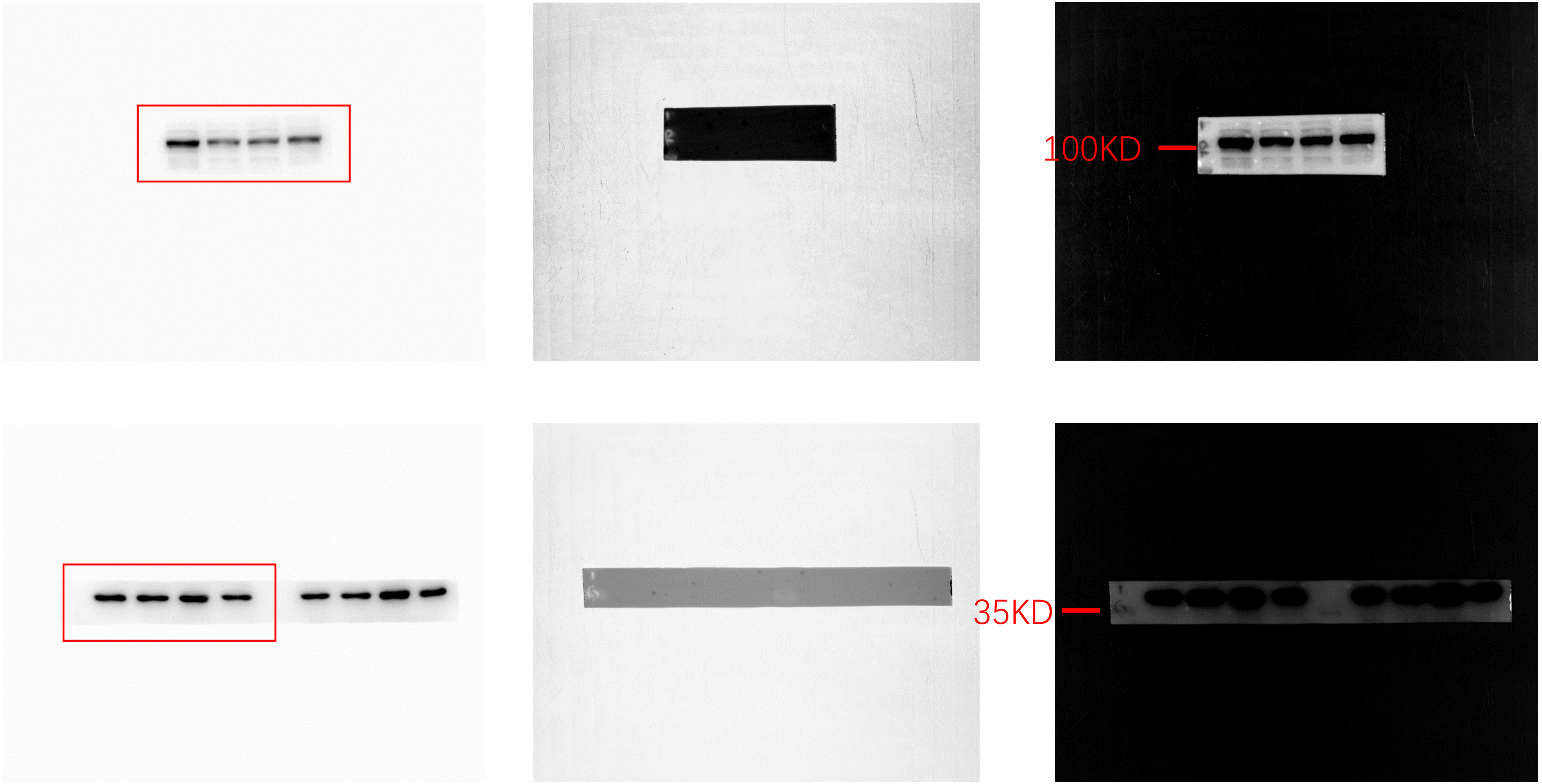

Supplementary Fig. 1G

IB: p-IR

IB: IR

IB: GAPDH

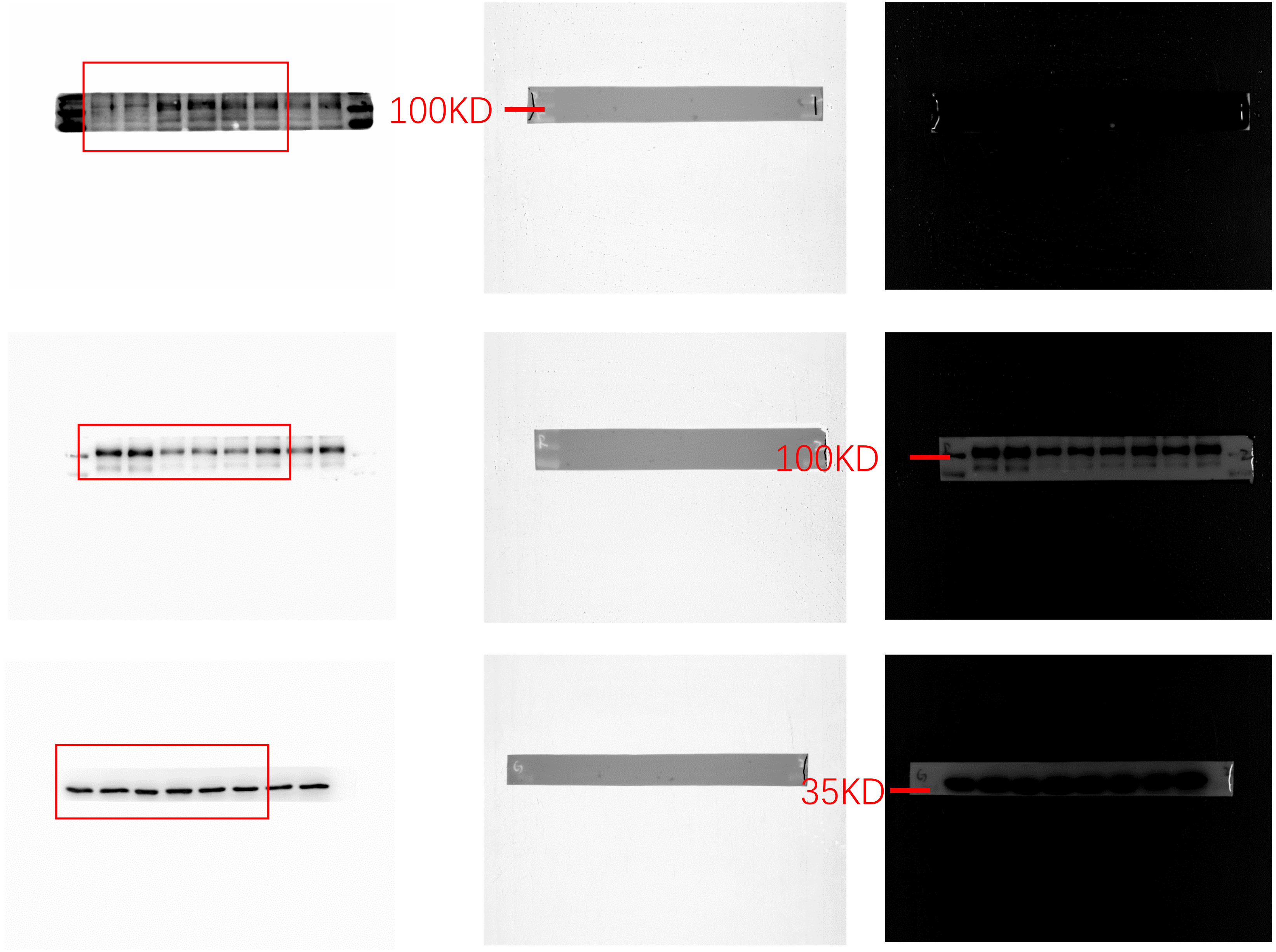

Supplementary Fig. 2A

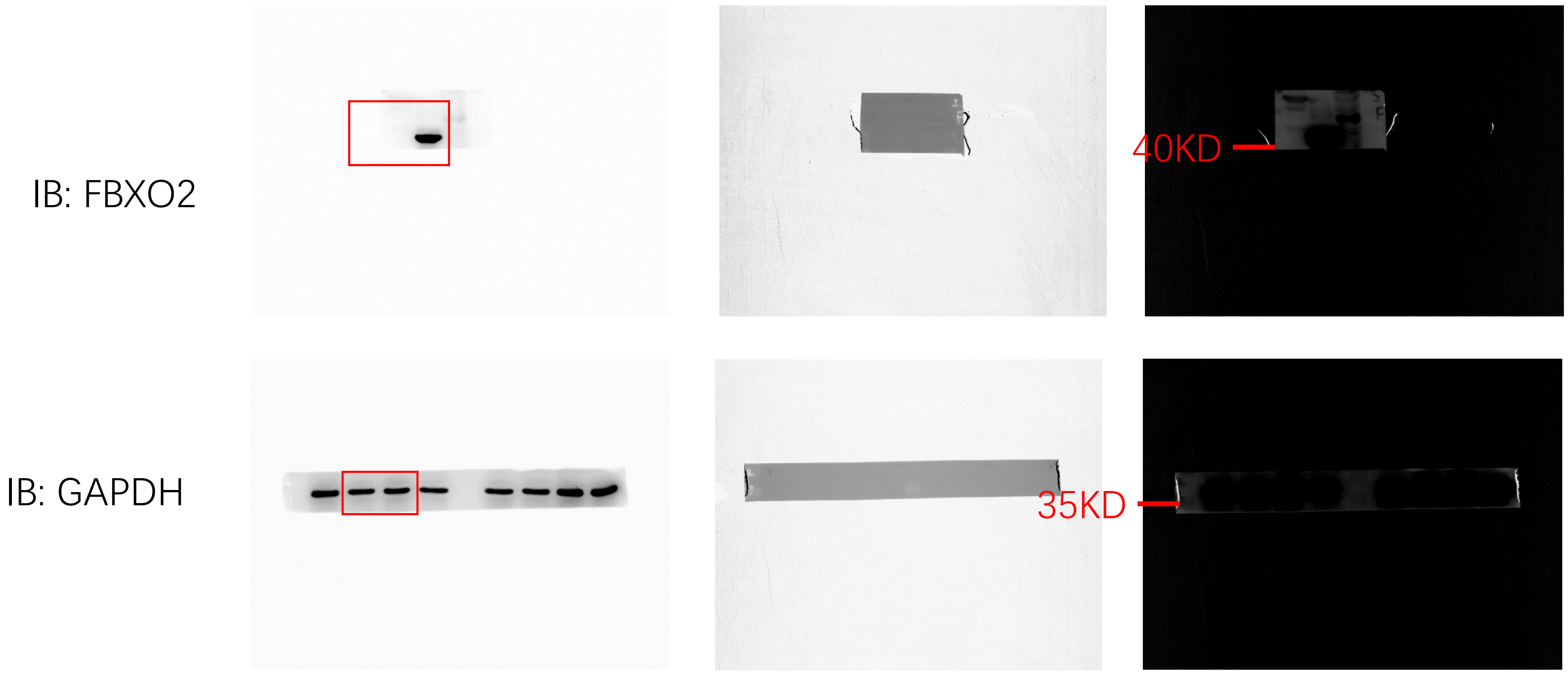

Supplementary Fig. 2B

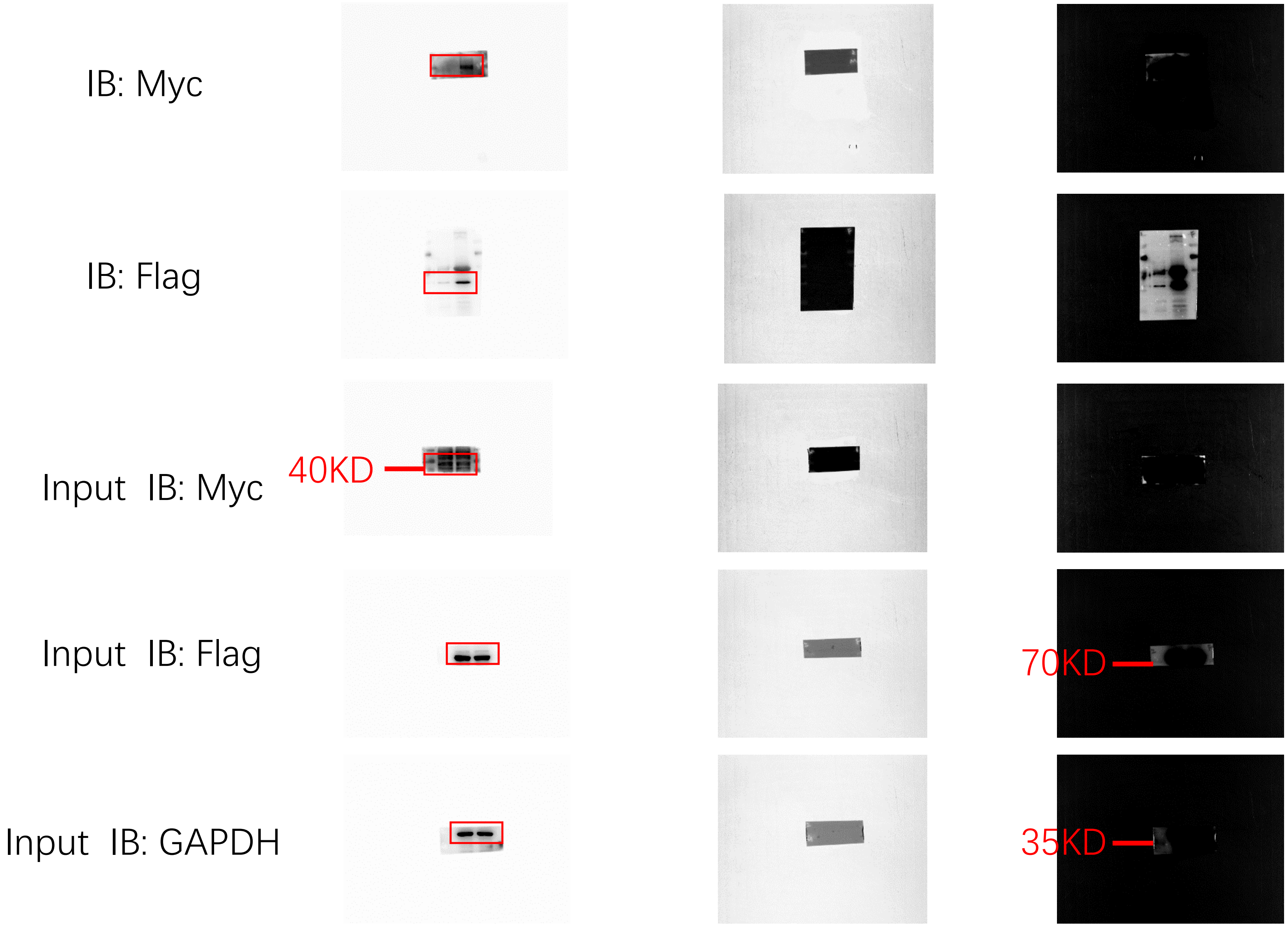

Supplementary Fig. 2C

IB: IR

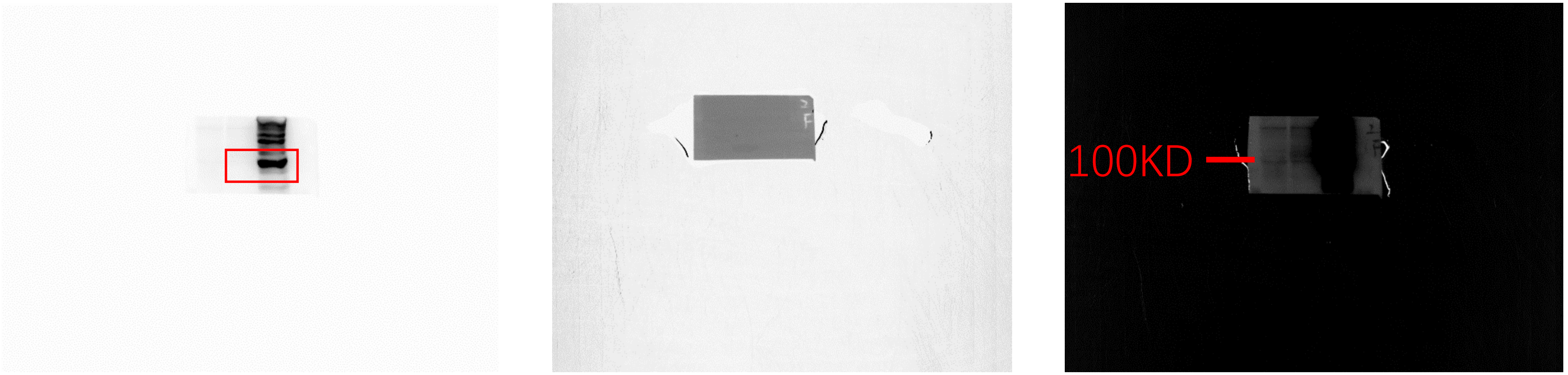

IB: GAPDH

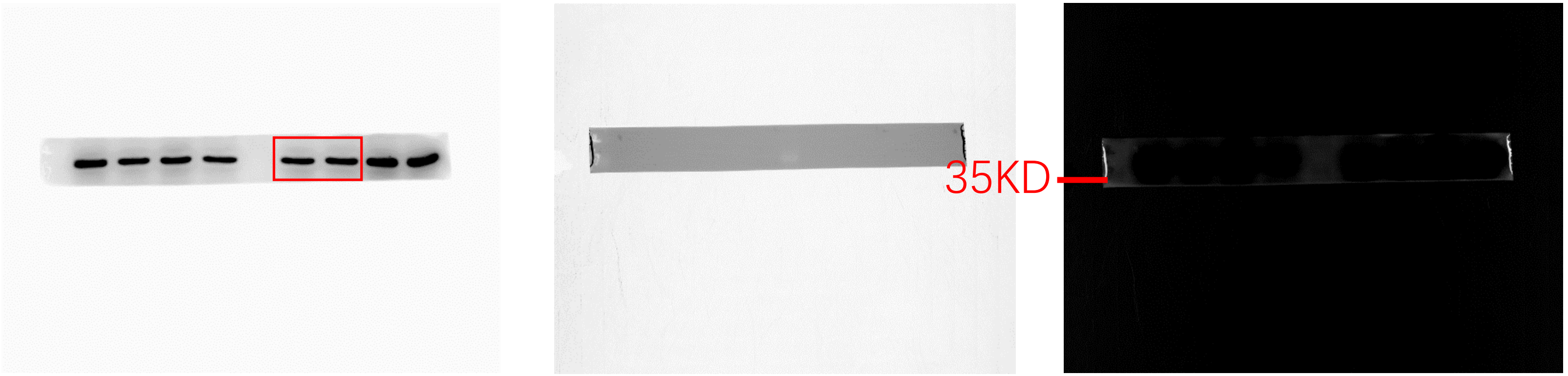

Supplementary Fig. 2G

IB: p-IR

IB: IR

IB: p-IRS1

IB: IRS1

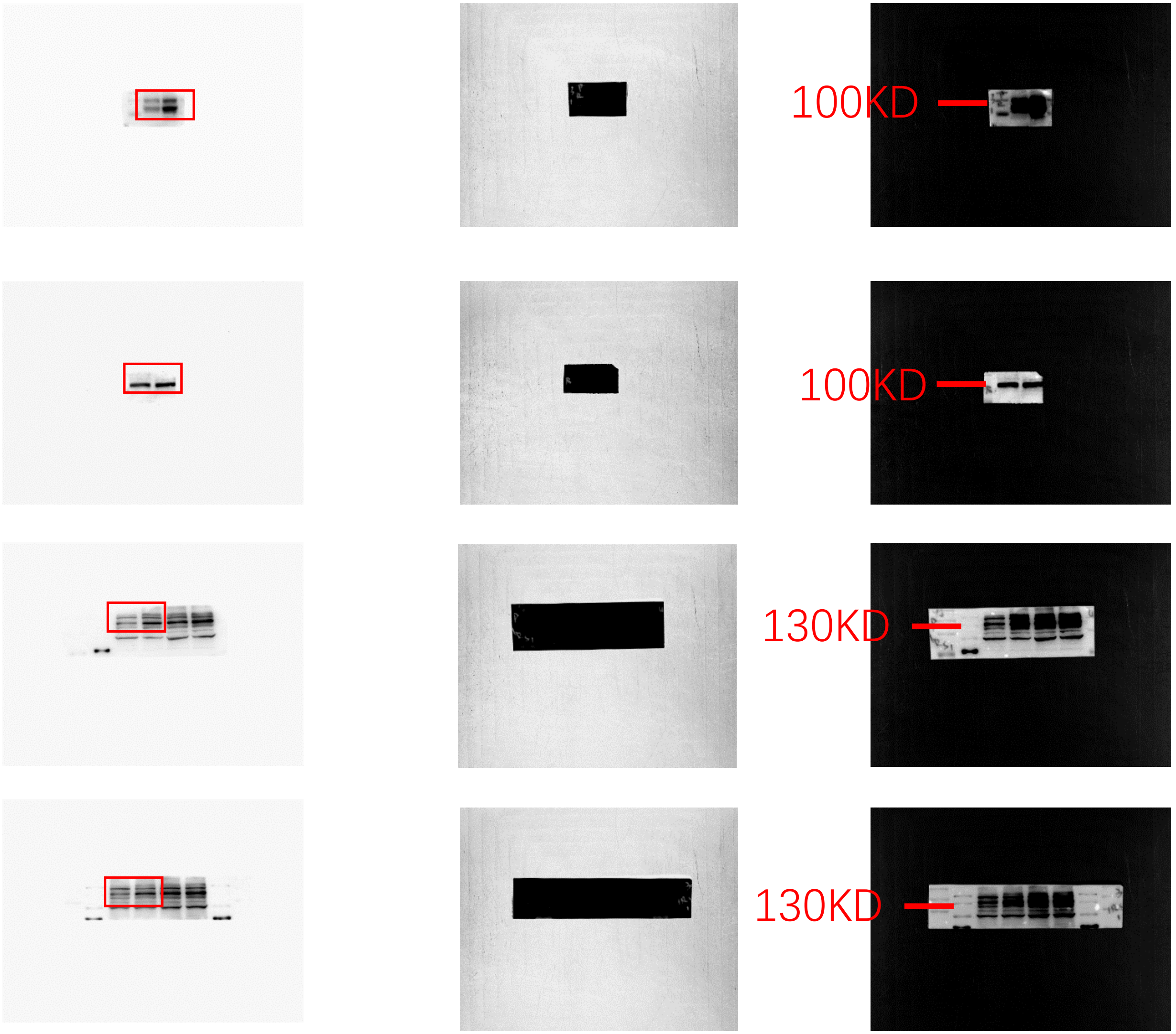

Supplementary Fig. 2G

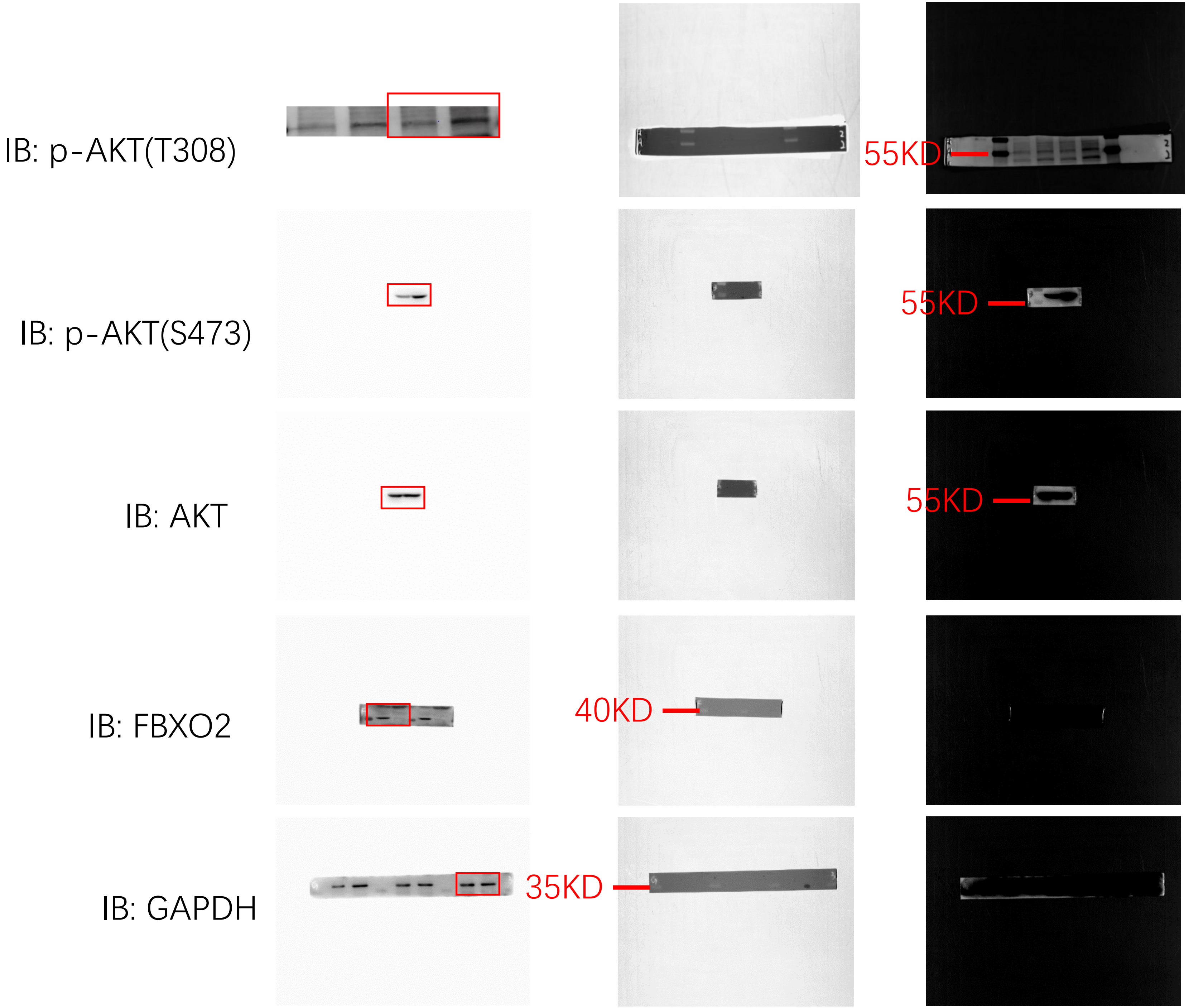

Supplementary Fig. 3C

IB: FBXO2

IB: GAPDH

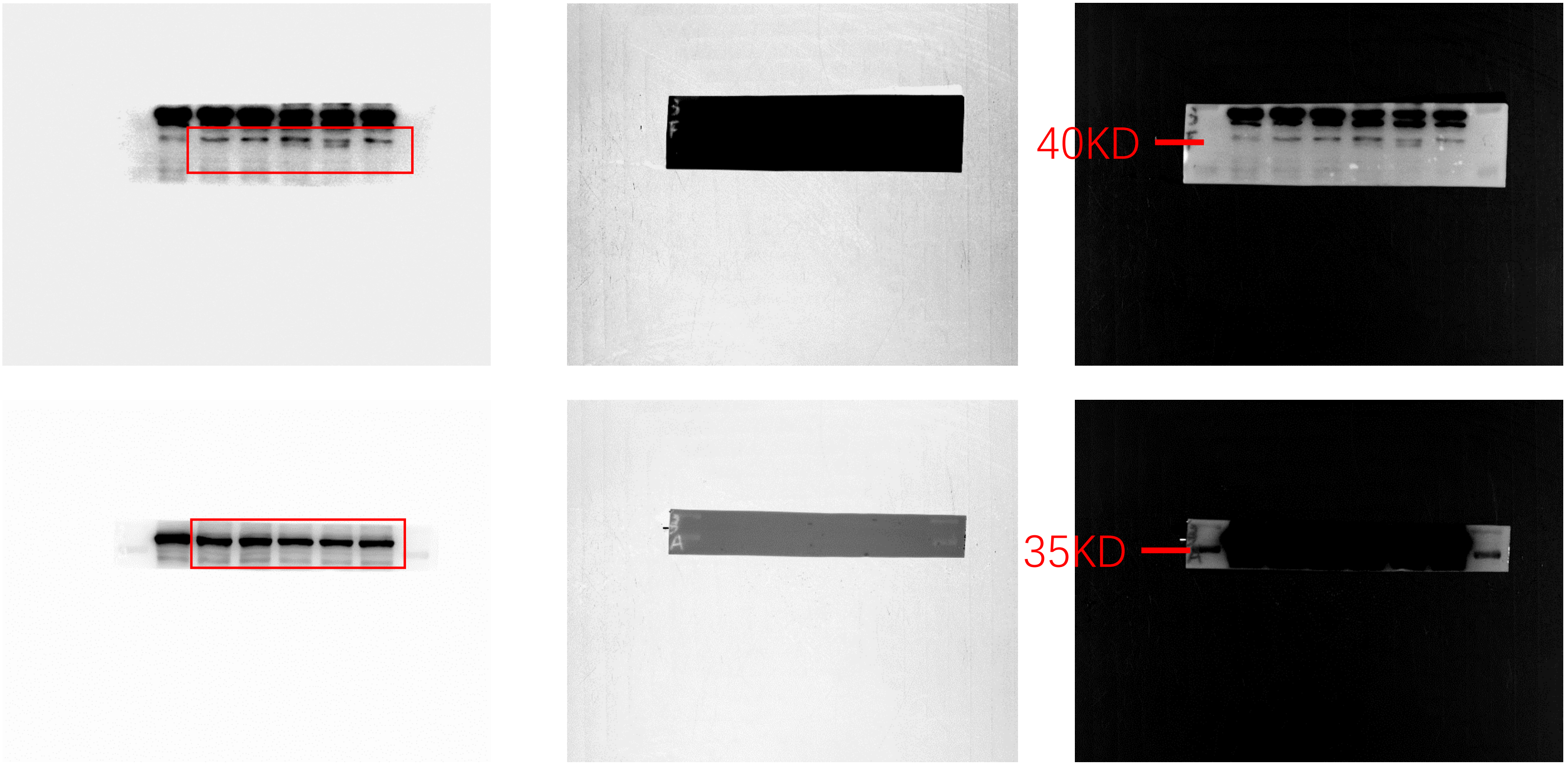

Supplementary Fig. 5A

Supplementary Fig. 5G

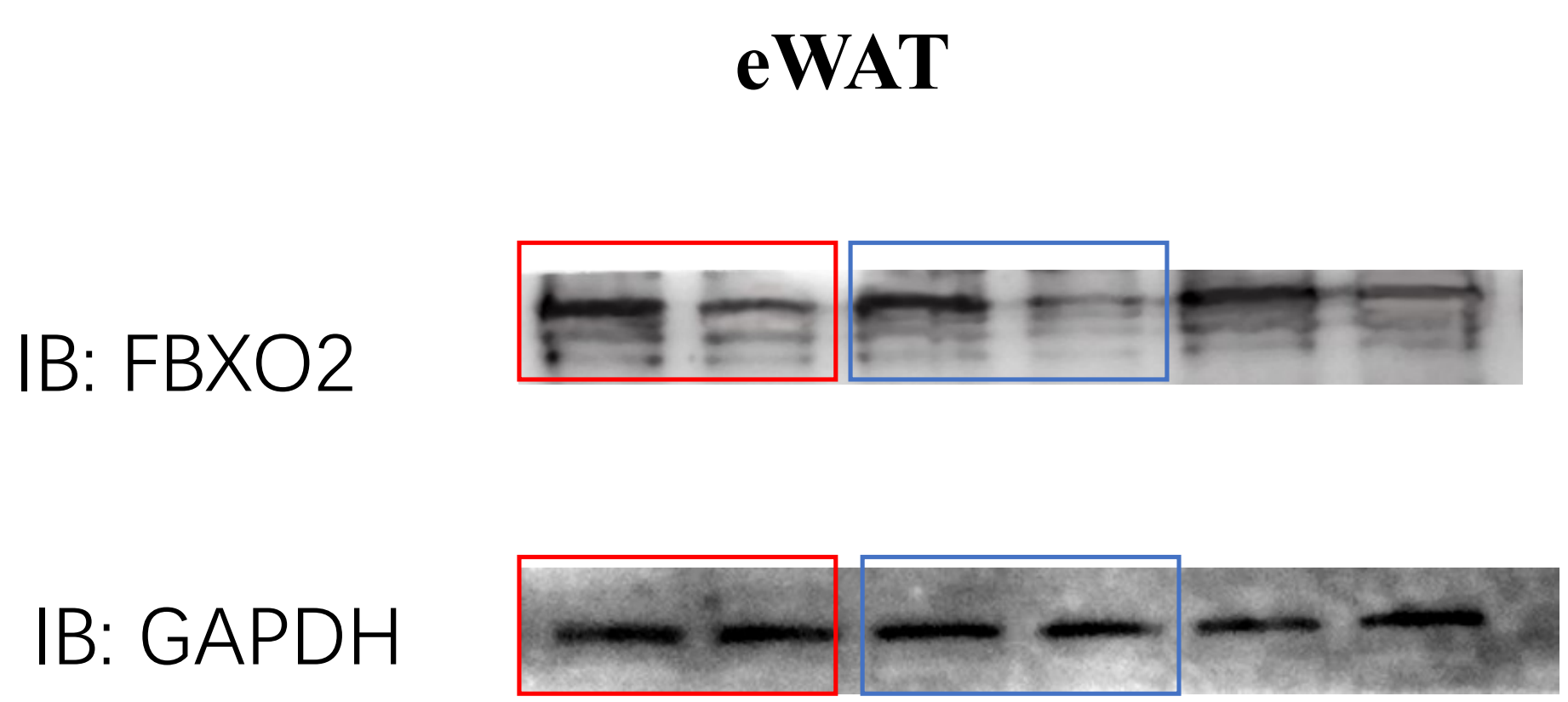

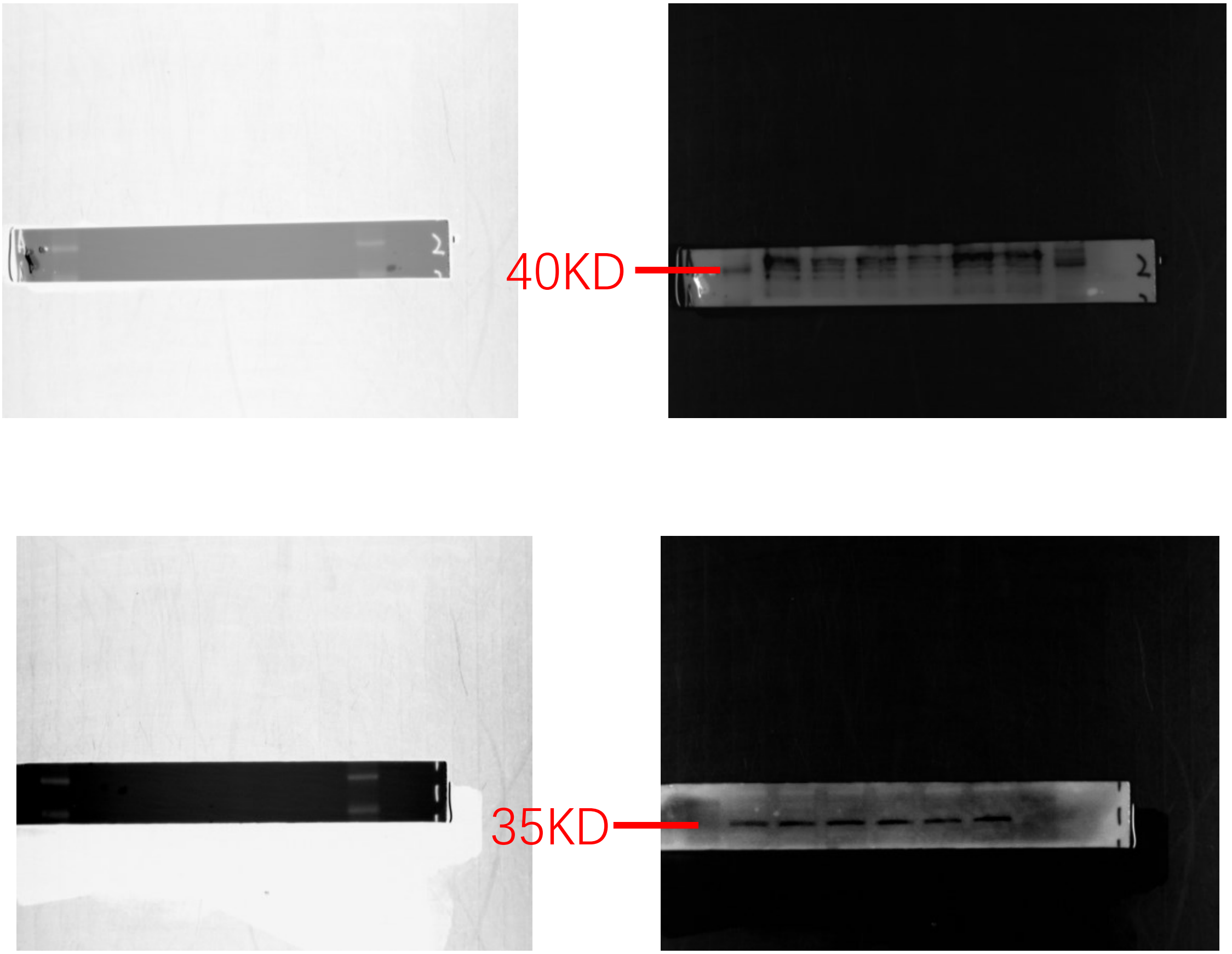

Supplementary Fig. 5A

Supplementary Fig. 5G

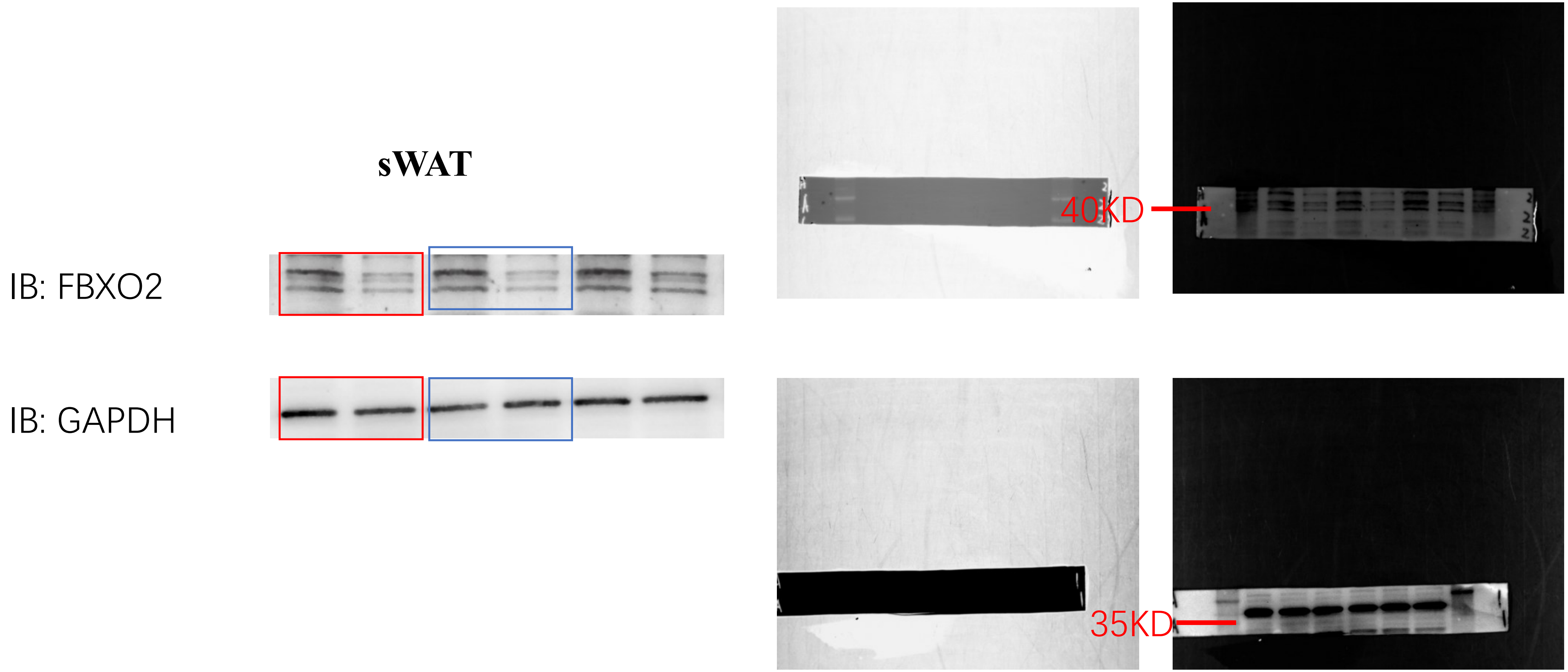

Supplementary Fig. 5A

Supplementary Fig. 5G

Liver

IB: FBXO2

IB: GAPDH

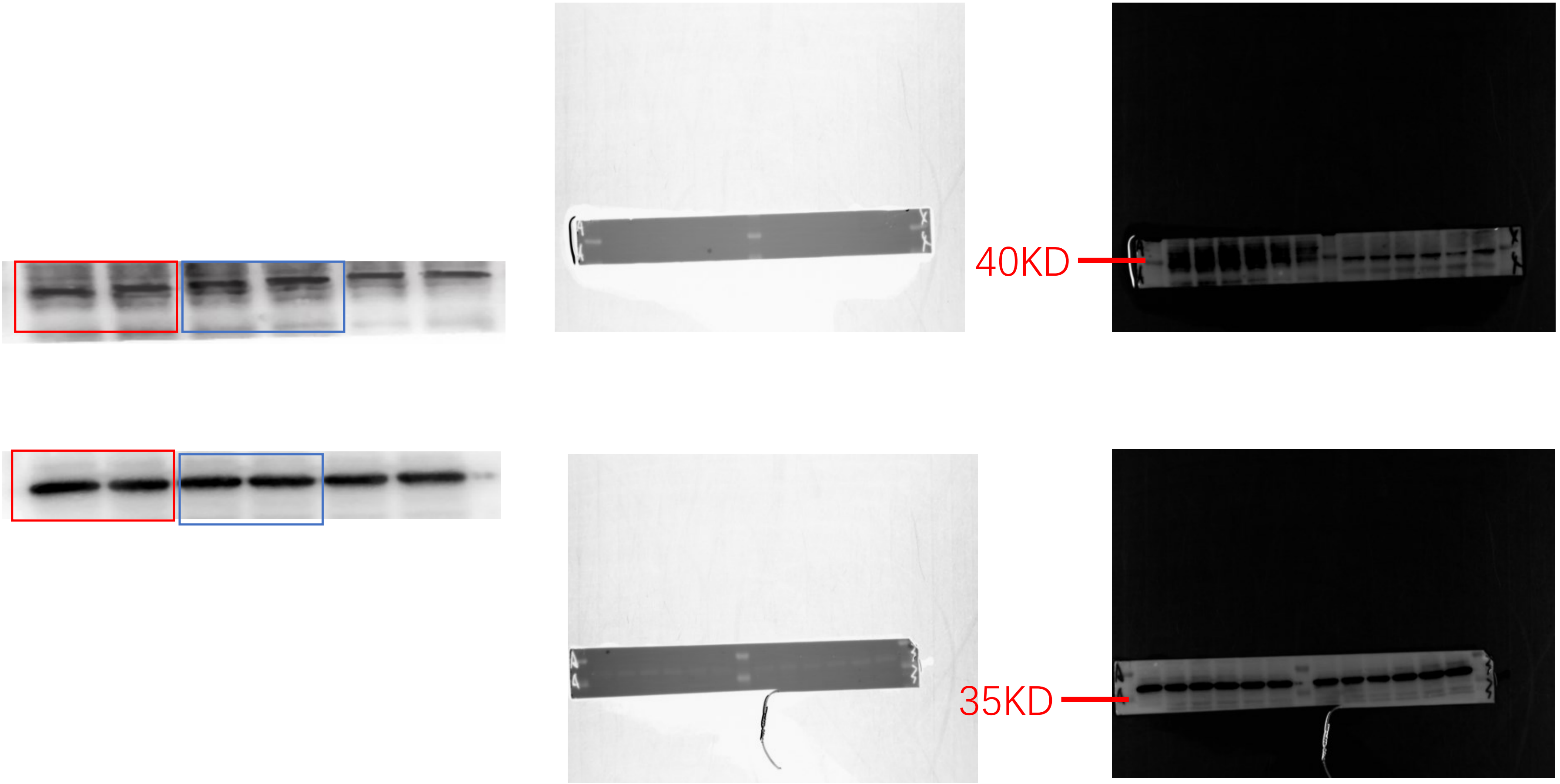

Supplementary Fig. 5J

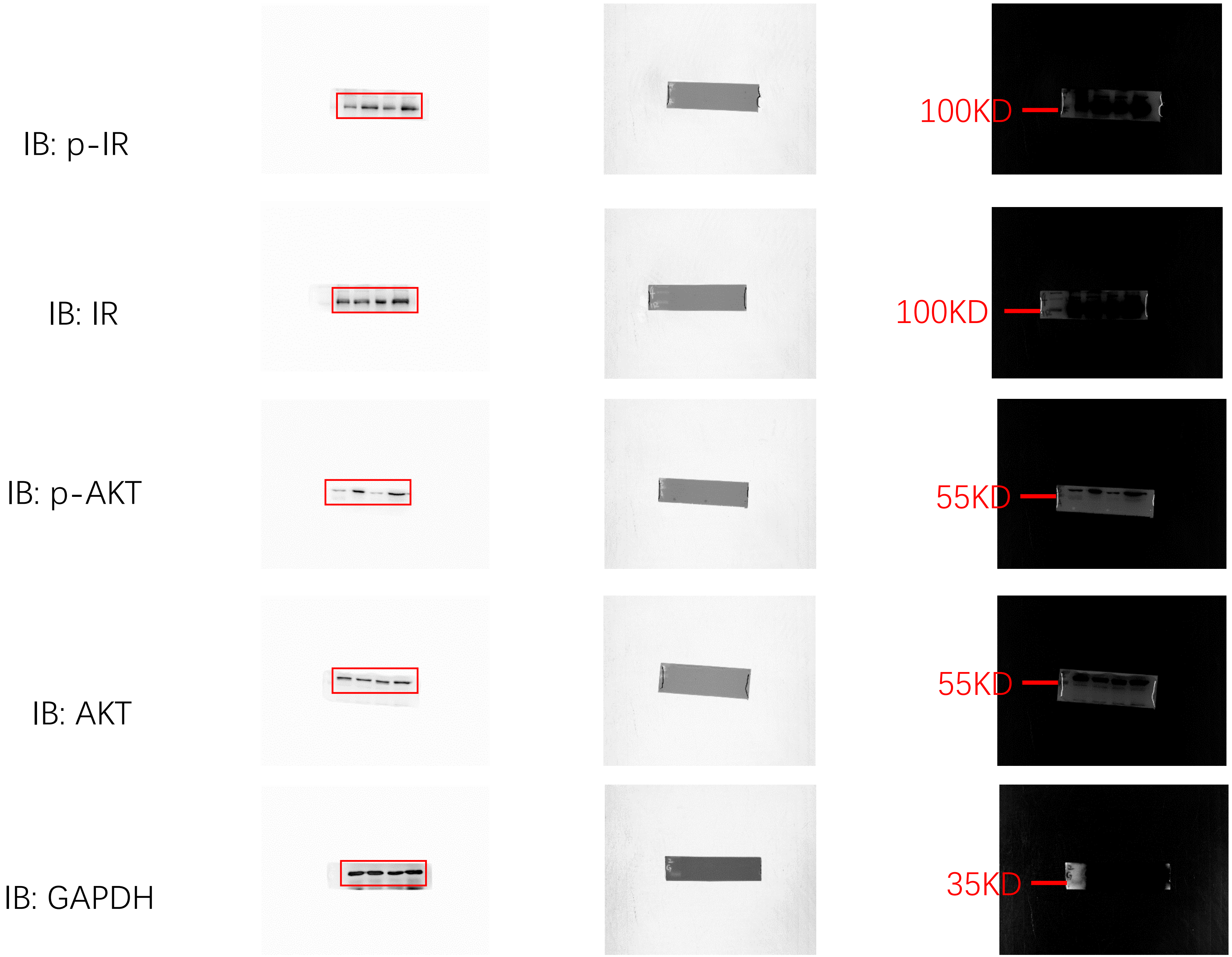

Supplementary Fig. 6A

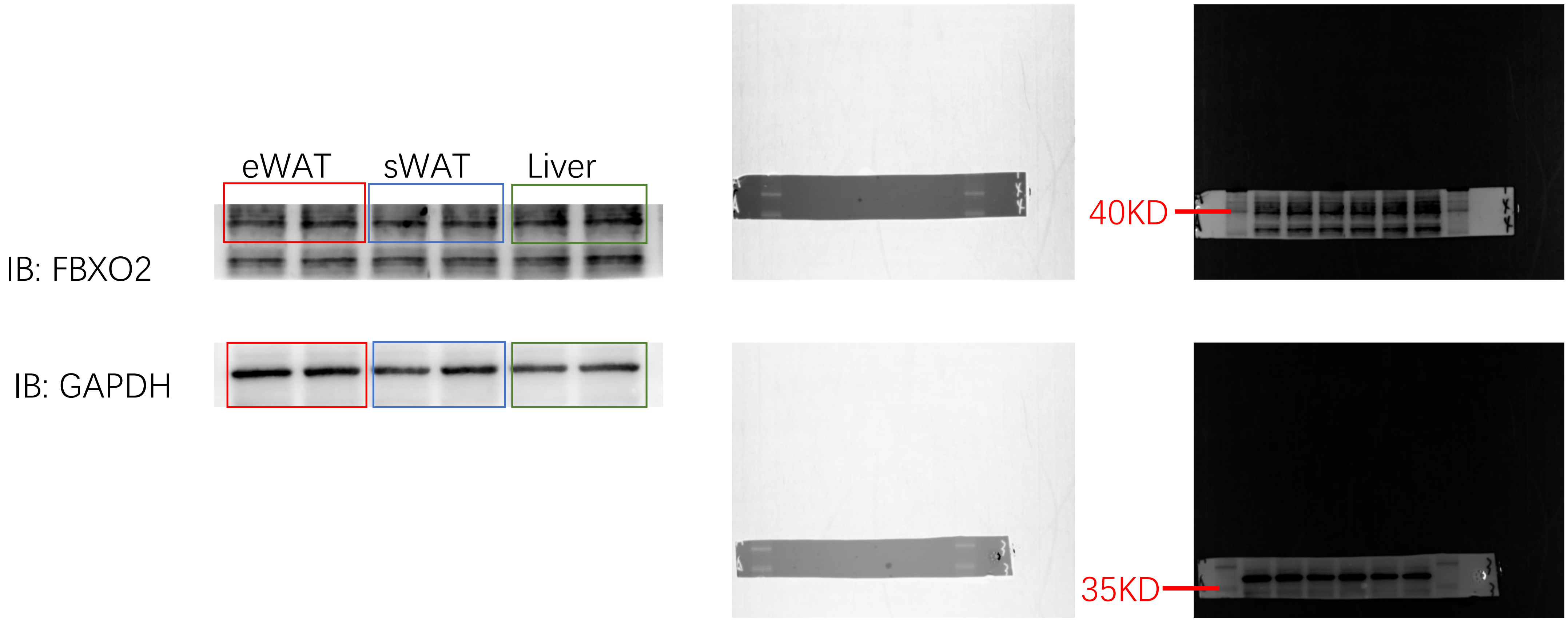

Supplementary Fig. 10A

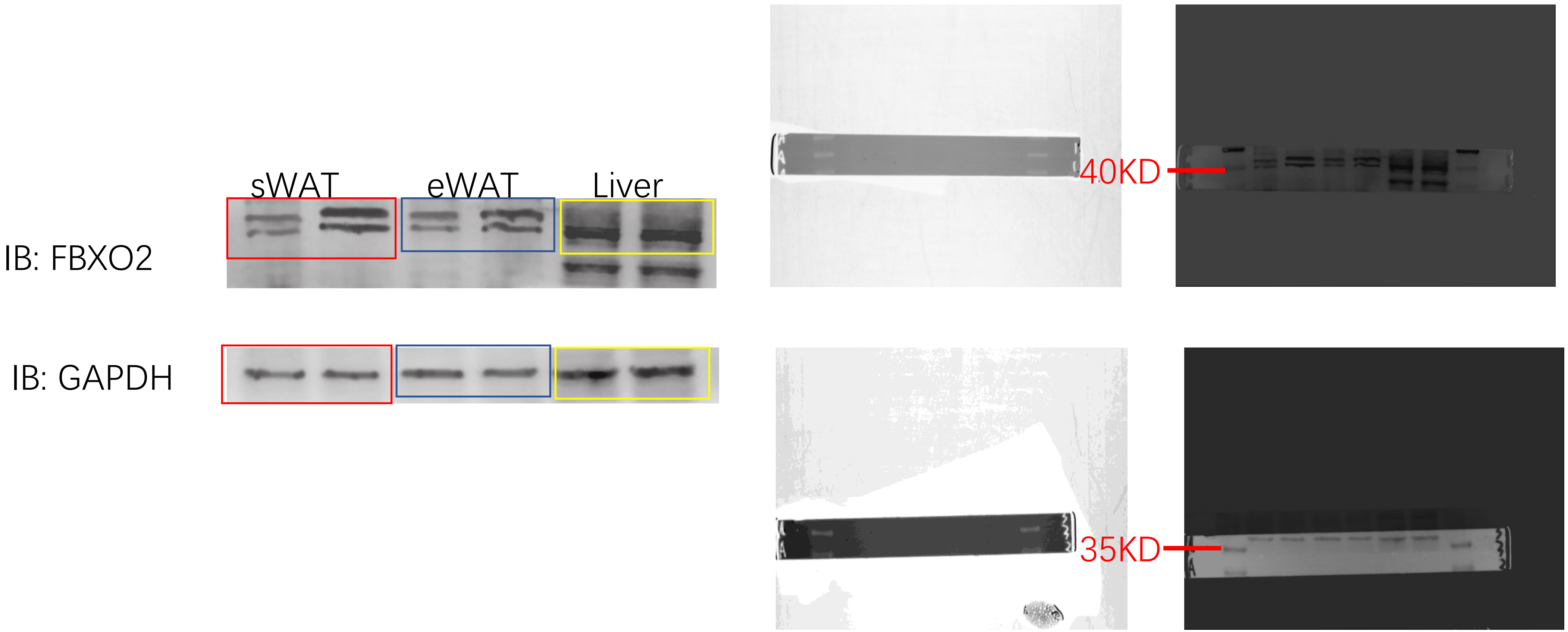

Supplement: Supplementary file 1 [file 41419_2026_9096_MOESM1_ESM.pdf]
