# Supplementary material for: E3 ligase FBXO2-mediated protein stability of insulin receptor regulates adipogenesis and metabolic health in obesity
Source: Cell Death Dis. 2026 Jul 8;17(1):848. doi: 10.1038/s41419-026-09096-z (PMC13631264; doi:10.1038/s41419-026-09096-z)
Supplement: Supplementary file 3 [file 41419_2026_9096_MOESM17_ESM.docx]

Fig. S1 The reduction of IR protein is closely associated with the ubiquitin/proteasome-dependent degradation pathway during early adipogenic differentiation.

A Insulin receptor (IR) protein levels during adipogenic differentiation of SVF cells at day 0, 2, 4, and 8. Protein densitometry analysis was conducted (right panel). B Insulin receptor (IR) mRNA levels during adipogenic differentiation of SVF cells at day 0, 2, 4, and 8. C Insulin receptor (IR) protein levels during adipogenic differentiation of SVF cells at 0, 12, 24, and 48 hours. Protein densitometry analysis was conducted (right panel). D Insulin receptor (IR) mRNA levels during adipogenic differentiation of SVF cells at 0, 12, 24, and 48 hours. E Insulin receptor (IR) mRNA levels during adipogenic differentiation of 3T3-L1 cells at day 0, 12, 24, and 48. F Protein densitometry analysis was conducted for Fig 1D. G p-IR/IR protein levels during adipogenic differentiation of SVF cells were treated with 20 μM MG132 for 2 hours before harvest. Protein densitometry analysis was conducted (right panel). Statistical data are presented as mean ± SEM of three independent experiments, and statistical analysis was performed using unpaired *t*-test and two-way ANOVA. **P<0.05, **P<0.01, ***P<0.001*, n=3.

Fig. S2 Ubiquitination and degradation of IR is catalysed by E3 ligase FBXO2.

A Column-bound proteins were confirmed by western blotting from the mass spectrometry analysis of IR. B Co-immunoprecipitation of IR with FBXO2 in HEK293T cells transfected with IR-Flag and FBXO2-Myc. C Column-bound proteins were confirmed by western blotting from the mass spectrometry analysis of FBXO2. D Validation of FBXO2 overexpression in 3T3-L1 cells by qRT-PCR. E *Insr* mRNA levels in 3T3-L1 cells with FBXO2 knockdown or overexpression. F Validation of FBXO2 knockdown in 3T3-L1 cells. G p-IR/IR, p-IRS1/IRS1, p-AKT(308)/AKT protein levels in 3T3-L1 cells with FBXO2 knockdown. H Schematic diagram of plasmid construction depicting the deletion of different domains of FBXO2. Statistical data are presented as mean ± SEM of three independent experiments, and statistical analysis was performed using unpaired *t*-test. ***P<0.01, ***P<0.001*, n=3.

Fig. S3 FBXO2 inhibits proliferation of preadipocytes.

A *Fbxo2* mRNA levels during adipogenic differentiation of SVF cells at day 0, 2, 4, 6, and 8. B Fbxo2 mRNA levels during adipogenic differentiation of 3T3-L1 cells at day 0, 2, 4, and 8. C FBXO2 protein levels during adipogenic differentiation of SVF at day 0, 0.5, 1, 2. D Protein densitometry analysis for (C). E Validation of FBXO2 knockdown in SVF cells from subcutaneous white adipose tissue (sWAT) and epididymal white adipose tissue (eWAT) by qRT-PCR. F,G mRNA levels of proliferative markers in SVF cells from sWAT (F) and eWAT (G) with FBXO2 knockdown. H,I mRNA levels of apoptosis genes in SVF cells from sWAT (H) and eWAT (I) with FBXO2 knockdown. Statistical data are presented as mean ± SEM of three independent experiments, and statistical analysis was performed using unpaired *t*-test. **P<0.05, **P<0.01,* n=3.

Fig. S4 FBXO2 inhibits adipogenesis by negatively regulating insulin signaling pathway *in vitro*.

A mRNA levels of adipogenic genes during differentiation of SVF from sWAT with FBXO2 knockdown. B Oil red O staining of SVF cells from sWAT with FBXO2 knockdown, scale bars, 20 μm. C Spectrophotometric measurement to determine the absorbance values of Oil red O staining solution. D mRNA levels of adipogenic genes during adipocyte differentiation of SVF cells from eWAT with FBXO2 knockdown. E Oil red O staining of SVF cells from eWAT with FBXO2 knockdown, scale bars, 20 μm. F Spectrophotometric measurement to determine the absorbance values of Oil red O staining solution. Statistical data are presented as mean ± SEM of three independent experiments, and statistical analysis was performed using unpaired *t*-test. **P<0.05, **P<0.01*, n=3.

Fig. S5 FBXO2 deficiency in adipocyte progenitor triggers hyperplastic expansion of sWAT by enhancing the insulin signaling pathway.

A Validation of FBXO2 knockdown in eWAT, sWAT and liver from control and *Fbxo2*^Pdgfrα-/-^ mice. B Body weight of control and *Fbxo2*^Pdgfrα-/-^ mice fed with NCD. C Percentage of tissue weight to body weight in control and *Fbxo2*^Pdgfrα-/-^ mice fed with NCD. D mRNA levels of proliferative and apoptosis genes in sWAT of control and *Fbxo2*^Pdgfrα-/-^ mice fed with NCD. E Protein densitometry analysis was conducted for Fig 5E. F mRNA levels of proliferation, apoptosis, and adipogenic genes in eWAT of control and *Fbxo2*^Pdgfrα-/-^ mice fed with NCD. G Validation of FBXO2 knockdown in eWAT, sWAT and liver from control and *Fbxo2*^Fabp4-/-^ mice fed with NCD. H Body weight of control and *Fbxo2*^Fabp4-/-^ mice fed with NCD. I Percentage of tissue weight to body weight in control and *Fbxo2*^Fabp4-/-^ mice fed with NCD. J Protein levels of p-IR/IR and p-AKT(308)/AKT in sWAT of control and *Fbxo2*^Fabp4-/-^ mice fed with NCD following intraperitoneal insulin injection. Protein densitometry of p-IR/IR/GADPH and p-AKT(308)/AKT/GAPDH analysis was conducted (right panel). Statistical data are presented as mean ± SEM of three independent experiments, and statistical analysis was performed using unpaired *t*-test and two-way ANOVA. **P<0.05, **P<0.01,***P<0.001*, n=6.

Fig. S6 Analysis of adipogenesis in *Fbxo2*^Adipoq-/-^ mice under NCD.

A Validation of FBXO2 knockdown in eWAT, sWAT and liver from control and *Fbxo2*^Adipoq-/-^ mice. B HE staining of sWAT in control and *Fbxo2*^Adipoq-/-^ mice fed with NCD, scale bar, 50 μm. Statistical analysis of adipocyte number and size observed in the under panel. C EdU staining of SVF from sWAT of NCD-fed control and *Fbxo2*^Adipoq-/-^ mice, scale bars, 50 μm. Statistical analysis of the EdU-positive cells in the right panel. D mRNA levels of proliferation, apoptosis, and adipogenic genes in sWAT of control and *Fbxo2*^Adipoq-/-^ mice fed with NCD. Statistical data are presented as mean ± SEM of three independent experiments, and statistical analysis was performed using unpaired t-test. No significance is indicated, *P>0.05*, n=6.

Fig. S7 FBXO2 deficiency in preadipocytes improved systemic metabolic health.

A-F Total cholesterol (TC) (A), free fatty acids (FFA) (B), insulin (C), leptin (D), alanine aminotransferase (ALT) (E), and aspartate aminotransferase (AST) (F) in serum of control and *Fbxo2*^Pdgfrα-/-^ mice fed with HFD. G Masson staining, IHC staining for F4/80, α-SMA of sWAT in control and *Fbxo2*^Pdgfrα-/-^ mice fed with HFD, scale bar, 50 μm. H,I mRNA levels of fibrosis (H) and inflammation (I) genes in sWAT of control and *Fbxo2*^Pdgfrα-/-^ mice fed with HFD. J mRNA levels of ATPsynβ, Cox7a1, Cox8b, Hif1α, and F4/80 in livers of control and *Fbxo2*^Pdgfrα-/-^ mice fed with HFD. K-P Total cholesterol (TC) (K), free fatty acids (FFA) (L), insulin (M), leptin (N), alanine aminotransferase (ALT) (O), and aspartate aminotransferase (AST) (P) in serum of control and *Fbxo2*^Fabp4-/-^ mice fed with HFD. Q Masson staining, IHC staining for F4/80, α-SMA of sWAT in control and *Fbxo2*^Fabp4-/-^ mice fed with HFD. R,S mRNA levels of fibrosis (R) and inflammation-related genes (S) in sWAT of control and *Fbxo2*^Fabp4-/-^ mice fed with HFD. T mRNA levels of ATPsynβ, Cox7a1, Cox8b, Hif1α, and F4/80 in livers of control and *Fbxo2*^Fabp4-/-^ mice. Statistical data are presented as mean ± SEM of three independent experiments, and statistical analysis was performed using unpaired *t*-test. **P<0.05, **P<0.01*, n=6.

Fig. S8 FBXO2 deficiency in mature adipocytes does not improve systemic metabolic health.

A,B Glucose tolerance test (A) and insulin tolerance test (B) of control and *Fbxo2*^Adipoq-/-^ mice fed with NCD or HFD for 10 weeks. C-I Metabolic parameters analysis of serum from control and *Fbxo2*^Adipoq-/-^ mice fed with HFD, including triglycerides (TG) (C), total cholesterol (TC) (D), free fatty acids (FFA) (E), insulin (F), adiponectin (G), alanine aminotransferase (ALT) (H), and aspartate aminotransferase (AST) (I). J HE staining, Masson staining, and IHC staining for F4/80, α-SMA in livers of control and *Fbxo2*^Adipoq-/-^ mice fed with HFD, scale bar, 50 μm. K Masson staining, IHC staining for F4/80, α-SMA in sWAT and eWAT in control and *Fbxo2*^Adipoq-/-^ mice fed with HFD, scale bar, 50 μm. Statistical data are presented as mean ± SEM of three independent experiments, and statistical analysis was performed using unpaired t-test. No significance is indicated, *P>0.05*, n=6.

Fig. S9 FBXO2 deficiency in preadipocytes does not regulate thermogenesis of brown and beige fat.

A,B Average food intake (A) and water drink (B) of control and *Fbxo2*^Pdgfrα-/-^ mice fed with NCD for 10 weeks. C,D mRNA levels of thermogenic genes in BAT (C) and sWAT (D) of control and *Fbxo2*^Pdgfrα-/-^ mice. E-K Oxygen consumption (E, F), carbon dioxide exhalation (G, H), energy expenditure (I, J) and activity (K) of control and *Fbxo2*^Pdgfrα-/-^ mice. L-M Food intake (L) and water drink (M) of control and *Fbxo2*^Pdgfrα-/-^ mice with CL316,243 stimulation (1mg/kg i.p.). N Rectal temperature of control and *Fbxo2*^Pdgfrα-/-^ mice with CL316,243 stimulation. O,P mRNA levels of thermogenic genes in sWAT (O) and BAT (P) of control and *Fbxo2*^Pdgfrα-/-^ mice with CL316,243 stimulation. Q-T Oxygen consumption (Q), carbon dioxide exhalation (R), energy expenditure (S) and activity (T) of control and *Fbxo2*^Pdgfrα-/-^ mice with CL316,243 stimulation. Statistical data are presented as mean ± SEM of three independent experiments, and statistical analysis was performed using unpaired t-test. No significance is indicated, *P>0.05*, n=4.

Fig. S10 FBXO2 overexpression inhibited adipogenesis and impaired glucose metabolism of mice. A Validation of FBXO2 overexpressed in eWAT, sWAT and liver from control and FBXO2 overexpressed mice. B Body weight of control and FBXO2 overexpressed mice with a short-term HFD feeding for 4 weeks, n=7. C Percentage of fat mass to body weight in control and FBXO2 overexpressed mice in (B). D Body weight of control and FBXO2 overexpressed mice with a long-term HFD feeding for 16 weeks. E Percentage of fat mass to body weight in control and FBXO2 overexpressed mice in (D). F Representative images of sWAT sections of mice with short term HFD feeding analysed by HE staining and IHC for PDGFRα. Statistical analysis of adipocyte number and size in the lower panel. G qRT-PCR analysis of proliferative, apoptosis, and adipogenic genes expression in sWAT of mice with short term HFD feeding. H,I GTT and ITT analysis of control and FBXO2 overexpressed mice under short term HFD feeding. Statistical data are presented as mean ± SEM of three independent experiments, and statistical analysis was performed using unpaired t-test. **P<0.05*, n=8.
